# Long-lived azobispyrazole stapled peptides for light-activated inhibition of protein–protein interactions

**DOI:** 10.1039/d6sc06137k

**Published:** 2026-11-02

**Authors:** Sinead McCann, Davia Prischich, Kira Scobel, Dennis J. Worm, Aditya R. Thawani, Marie Mori, Yaw Sing Tan, Anna Barnard, Matthew J. Fuchter

**Affiliations:** a Department of Chemistry, Molecular Sciences Research Hub, Imperial College London 82 Wood Lane London W12 0BZ UK a.barnard@imperial.ac.uk; b Department of Chemistry, University of Oxford 12 Mansfield Road Oxford OX1 3TA UK matthew.fuchter@chem.ox.ac.uk; c Bioinformatics Institute, Agency for Science, Technology and Research (A*STAR) 30 Biopolis Street, #07-01 Matrix Singapore 138671 Singapore

## Abstract

Photoswitches can be used to optically control the bioactive conformation of molecules through *E*–*Z* photoisomerisation, thereby enabling precise regulation of biological interactions with light. Herein, we describe the first use of a photoswitchable azobispyrazole cross-linker to generate light-responsive (*i*, *i* + 7)-stapled Bak^72–87^ and p53^17–28^ peptides. Photoisomerisation (*E* → *Z*) of the stapled peptides enhanced helical content and led to an increase in binding affinity for target proteins Bcl-x_L_ and MDM2, an effect that was reversible upon back-irradiation to the *E* isomer. The novel azobispyrazole cross-linker demonstrated excellent photochemical properties including near-quantitative *E* → *Z* and *Z* → *E* photoisomerisation and long thermal half-life of 65 days at 21 °C. These properties were retained in the stapled peptide form of AzBisPy-Bak^72–87^ (0.5-year half-life) and were tolerant to biological assay conditions. Our work expands the repertoire of photoswitches designed for peptide stapling and demonstrates the ability of azobispyrazole-stapled peptides to modulate protein–protein interactions in a system where binding is controlled exclusively by light, independent of thermal relaxation, over a timescale of several weeks.

## Abstract 2

Light-controlled, reversible binding of the AzBisPy – stapled peptide to the target protein.

## Graphical abstract

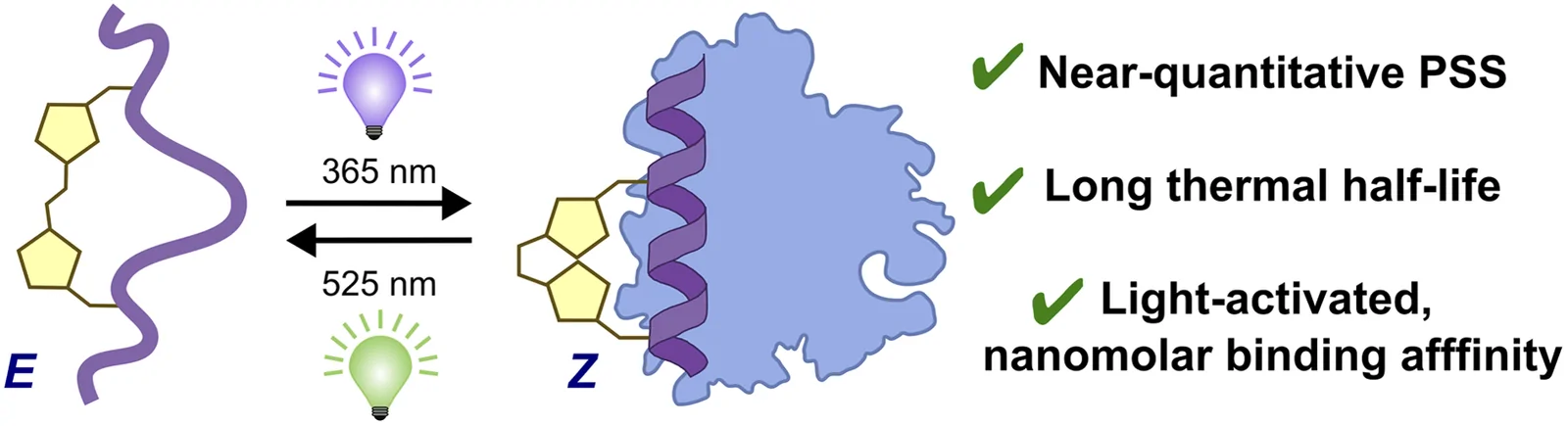

## Introduction

Protein–protein interactions (PPIs) are vital for nearly all cellular processes and typically occur between large protein contact surfaces (1500–3000 Å^2^).^1^ The α-helix is the most abundant protein secondary structure, with 62% of all protein–protein interactions involving an α-helical binding motif.^2,3^ As a result, significant effort has been devoted to the development of small-molecules^4^ and peptides^5–8^ to modulate helically-bound PPIs. However, short, isolated peptides rarely form α-helices in solution and there is an entropic barrier to folding into the correct secondary structure that can impair binding. Unstructured peptides are also more sensitive to proteolysis due to their accessible peptide backbone, further limiting their operational use.

The introduction of an intramolecular cross-linker, or ‘staple’, at suitably spaced *i* and *i* + *n* residues on the peptide backbone is a technique used to constrain the peptide into a helical confirmation and thus is an effective way to overcome these limitations.^5^ Two-component peptide stapling requires the installation of two natural or unnatural amino acids into the peptide sequence that have a functionality suitable for linkage *via* a chemical cross-linker. The residues can be covalently linked across one helical turn (*i*, *i* + 3 and *i*, *i* + 4) or across two or more turns (*i*, *i* + 7 and *i*, *i* + 11).^5^ The incorporation of a photoswitchable motif into the peptide staple enables optical control over the degree of peptide helicity through *E*/*Z* photoisomerisation. Therefore, through irradiation with a specific wavelength of light, modulation of the targeted PPI with tight spatial and temporal control can be achieved.^9^ From a photopharmacology^10–18^ perspective, the ability to site-selectively switch between a ‘low’ and ‘high’ state of bioactivity heralds the possibility of greater precision and fewer downstream side-effects in their application as therapeutics or chemical tools.

Woolley and co-workers pioneered the use of azobenzene as a photoswitchable cross-linker for stapling of peptides containing a pair of *i* and *i* + *n* cysteine residues.^19–29^ The modification of the azobenzene core with *ortho*-amino,^22^*ortho*-methoxy^19–21,30^ or *ortho*-chloro^30^ groups was shown to red-shift the absorption spectra of the staple, enabling visible and later red and near-infrared light photoswitching. This provides significant advantages *in vivo* as it bypasses the toxicity and poor tissue penetration associated with UV light. A notable design is the azobenzene cross-linker, AzB, bearing solubilising sulfonate groups and reactive haloacetamide ends for cysteine-based stapling (Fig. 1a).^27,30–36^ Allemann and co-workers^35^ generated a series of AzB-stapled Bak^72–87^ peptides that were designed to target Bcl-x_L_ and enabled reversible switching between a ‘low’ and ‘high’ activity state.^35^ The interaction between Bak and Bcl-x_L_ is a well-validated oncological target, with the overexpression of anti-apoptotic Bcl-x_L_ protein linked to tumorigenesis and chemoresistance.^37,38^

**Fig. 1:**
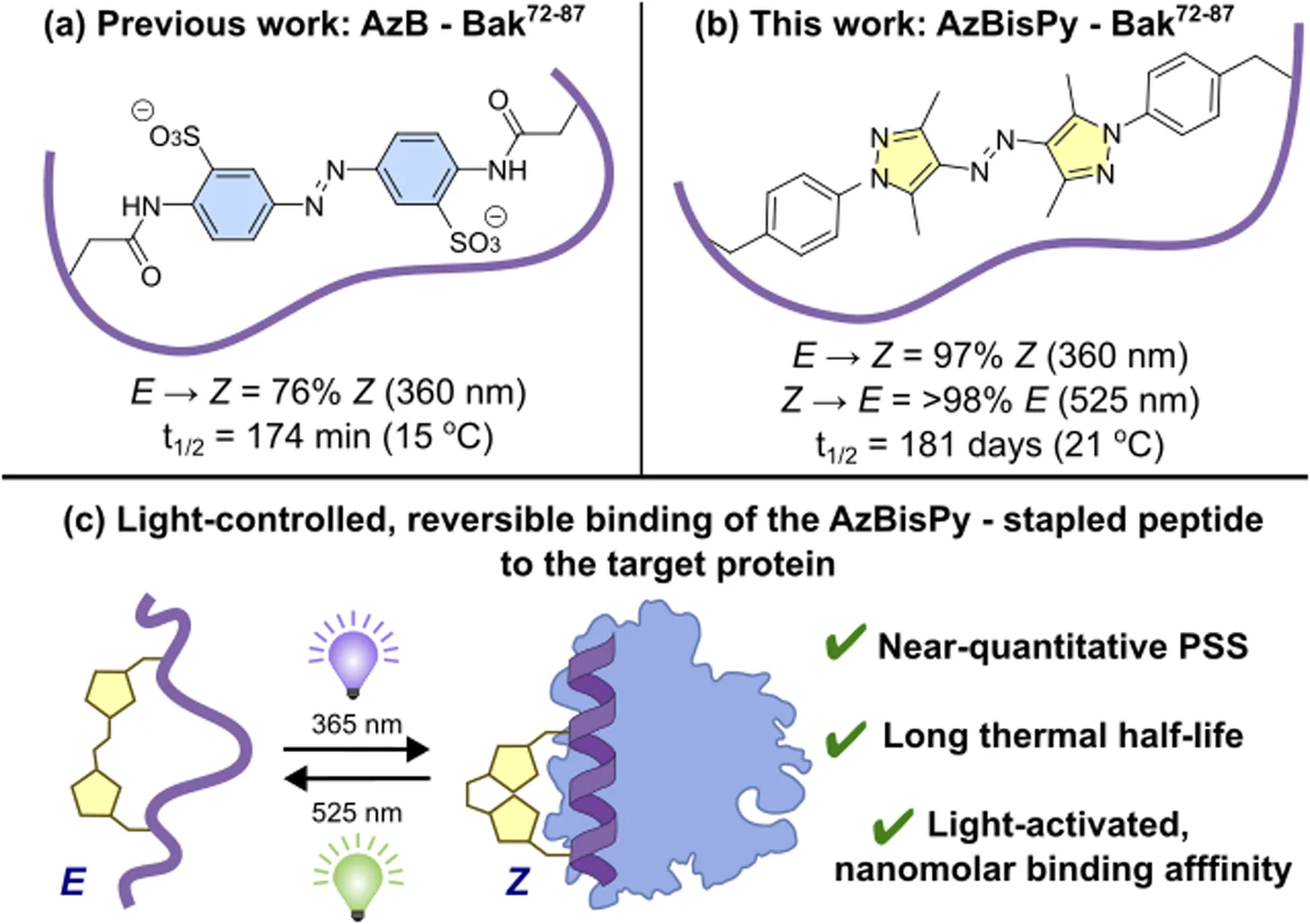
Comparison of the photochemical properties of (a) AzB-stapled Bak^72–87^ and (b) AzBisPy-stapled Bak^72–87^; (c) a graphical summary depicting the activation of the azobispyrazole photoswitchable stapled peptides through *E* to *Z* photoisomerisation resulting in enhanced peptide helicity and binding affinity to the target protein.

The replacement of one or both phenyl groups with heteroaromatic rings is a further modification that can be used to tune the photophysical properties in photoswitch design,^10,39–51^ and whilst these structures have been incorporated into peptide backbones,^52,53^ they have yet to be employed in peptide stapling. One particularly exciting example for a symmetrical switch design is azobispyrazole, which has emerged as a new class of photoswitch with near-quantitative, bidirectional photoconversion due to the distinct separation of the π–π* and n–π* absorbance bands.^39,40,54^ The azobispyrazole thermal half-life ranges from days to years, with the longest half-life of 681 days observed when linked *via* a 4-pyrazole ring.

Herein we present the first photoswitchable stapled peptides linked *via* a heteroaromatic switch cross-linker, specifically an azobispyrazole (AzBisPy) (Fig. 1b). A novel synthesis route and stapling conditions were developed to attach the photoswitch to the *i*, *i* + 7 cysteine residues of modified Bak and p53 peptides. Photoisomerisation (*E* → *Z*) of the AzBisPy-stapled peptides enhanced the helicity and switched the stapled peptide from a low to high-affinity binding state in the presence of their respective target proteins, Bcl-x_L_ and MDM2. The AzBisPy-Bak stapled peptide features excellent photochemical properties including near-quantitative *E* ↔ *Z* photoisomerisation, a highly stable *Z*-isomer and stability to photofatigue. This improvement on previous photoswitchable cross-linkers creates further opportunities for biological applications utilising the on/off mechanism of photoswitchable stapled peptides (Fig. 1c).

## Results and discussion

### Photoswitchable cross-linker design

Several criteria were considered for the design of a photoswitchable staple for cysteines in *i* and *i* + 7 positions. A photoswitchable element based on an azobispyrazole scaffold was selected given its capability for quantitative, bidirectional photoswitching and greatly enhanced half-life in comparison to azobenzene. The overall improvement in photochemical properties was hypothesised to translate into greater induction and control of secondary structure once the covalent attachment to the peptide had been achieved. The rigidity of the cross-linker was another key design factor as flexibility of the staple may result in a weak conformational change upon irradiation to the *Z* helical state. Therefore, a rigid phenyl spacer was designed adjacent to the azobispyrazole core, with alkyl halides for covalent attachment to the cysteine residues. Finally, the proposed AzBisPy was designed to approximately match the length of previously reported azobenzene cross-linkers for stapling *i*, *i* + 7 cysteines.

Synthesis of the AzBisPy cross-linker (Scheme 1) proceeded *via* hydrogenation of the pyrazole nitro group (1) to an amine (2). Diazotisation and *in situ* diazonium coupling with acetylacetone yielded (3), followed by a Knorr reaction with hydrazine, to give the azobispyrazole photoswitch core (4). The pyrazole *N*-arylation step proved challenging and various copper and palladium-catalysed coupling conditions were unsuccessful at yielding the desired product.^55,56^ In literature examples, this was attributed to the steric hindrance of the methylated pyrazole substrate, and thus bis-*N*-arylation of the photoswitch core was predicted to be difficult.^55,56^ Despite not being under catalytic conditions, a Chan–Lam coupling successfully yielded the coupled product (6), which following a TBS deprotection step, gave the alcohol (7) in 46% yield over two steps. Bromination of the alcohol with hydrobromic acid gave cross-linker AzBisPy (8), ready for stapling.

**Scheme 1:**
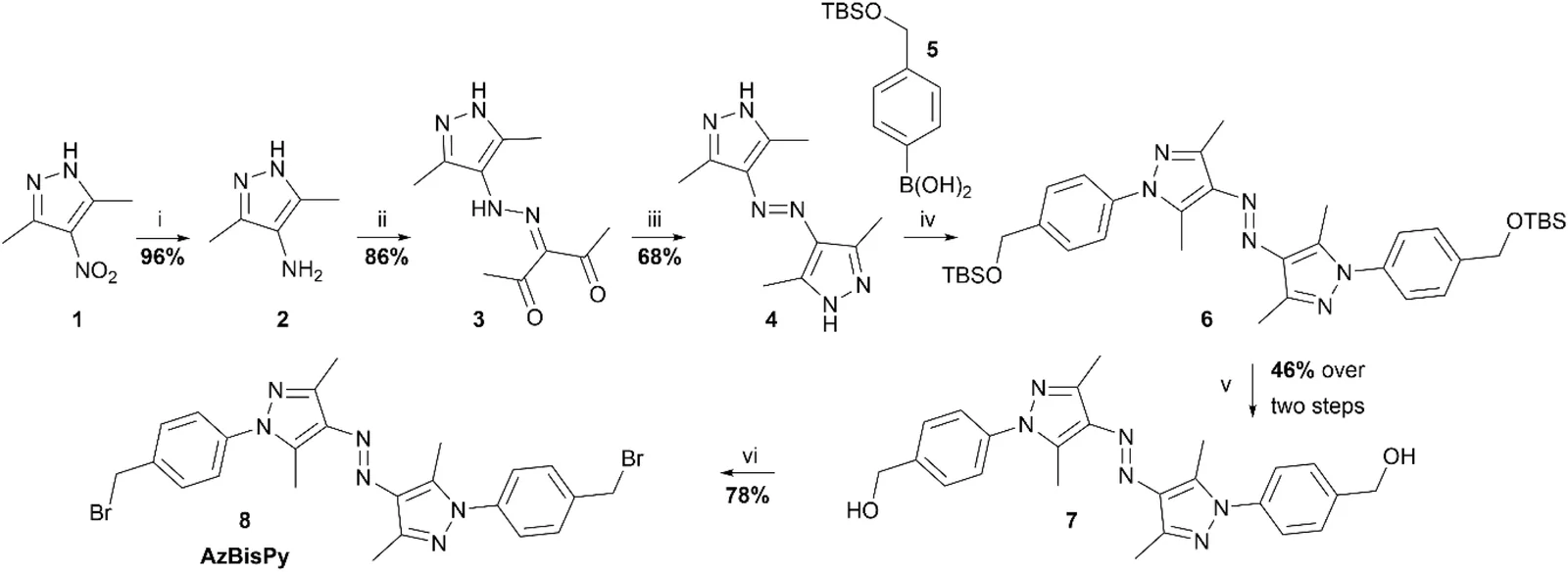
Reaction conditions: (i) H_2_, Pd/C, rt, 74 h; (ii) conc. HCl, NaNO_2_, H_2_O/MeOH, 0 °C, 45 min then acetylacetone, KOAc, 0 °C to rt, 2 h; (iii) NH_2_NH_2_·H_2_O, glacial AcOH, 90 °C, 16 h; (iv) 4-(TBSoxymethyl)phenylboronic acid 5, Cu(OAc)_2_, pyridine, DMF, 4 Å molecular sieves, rt, 22 h; (v) TBAF, THF, rt, 1 h; (vi) 48% aq. HBr, 50 °C, 22 h.

### Design and synthesis of photoswitchable stapled-Bak^72–87^ peptides

To develop a new class of AzBisPy-stapled Bak^72–87^ peptides, the wildtype Bak^72–87^ peptide 9 was synthesised and the previously described Bak^72–87^ peptide 10 was used as an initial design template for stapling (Table 1).^35^ In the modified peptide 10, the Gln73 and Ile81 residues from wildtype Bak^72–87^9 are substituted with alanine and phenylalanine, respectively, with the latter modification reported to increase the binding affinity to Bcl-x_L_.^57^ To enable stapling across the *i*, *i* + 7 positions, the Gln77 and Asp84 residues positioned on the non-binding face were substituted to cysteines.^58^ Peptide 10 was further modified to peptide 13, which was ultimately used for stapling, through the incorporation of a solubilising C-terminal poly-Arg tag, comprising of two aminohexanoic acid (Ahx) spacers and four arginine residues.^59^

**Table 1:** Sequences of synthesised Bak peptides. Ac: acetyl; FAM: 5(6)-carboxyfluorescein; * denotes stapling at *i*, *i* + 7 cysteine residues with an azobispyrazole cross-linker (AzBisPy); ° denotes stapling at *i*, *i* + 7 cysteine residues with an azobenzene cross-linker (AzB); Arg tag signifies the addition of –Ahx–Ahx–RRRR– to the peptide C-terminus, where Ahx = aminohexanoic acid

	Peptide	Sequence
9	Wildtype Bak^72–87^	Ac-GQVGRQLAIIGDDINR-NH_2_
FAM-9	FAM-Bak^72–87^	FAM-GQVGRQLAIIGDDINR-NH_2_
10	[A^73^,F^81^,C^77,84^]-Bak^72–87^	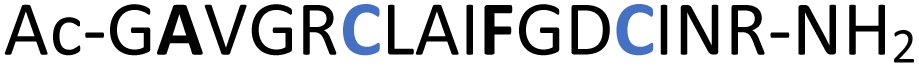
11	[A^73^,F^81^,C^77,84^]-Bak^72–87^ – AzB	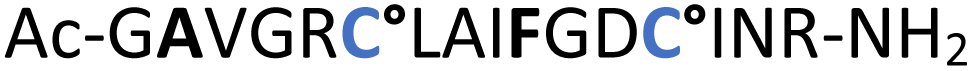
12	[A^73^,F^81^,C^77,84^]-Bak^72–87^ – AzBisPy	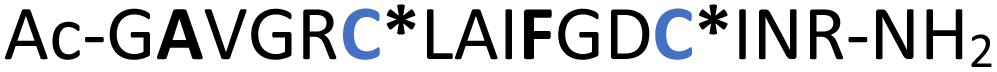
13	[A^73^,F^81^,C^77,84^]-Bak^72–87^ – Arg tag	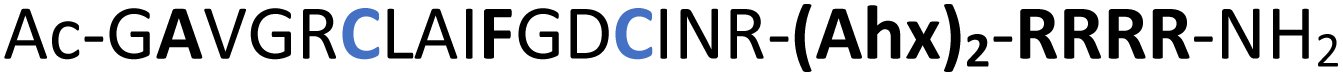
14	[A^73^,F^81^,C^77,84^]-Bak^72–87^ – Arg tag – AzBisPy	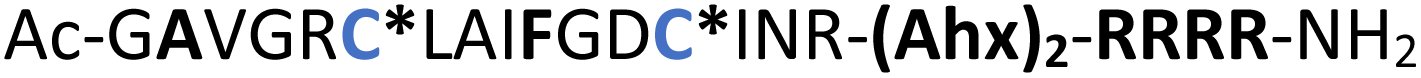
15	[A^73–74,78,81^,C^77,84^]-Bak^72–87^ – Arg tag	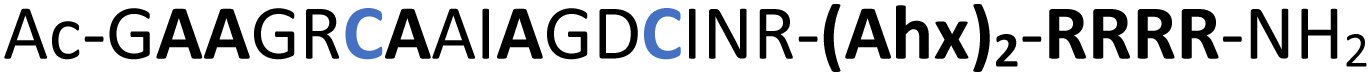
16	[A^73–74,78,81^,C^77,84^]-Bak^72–87^ – Arg tag – AzBisPy	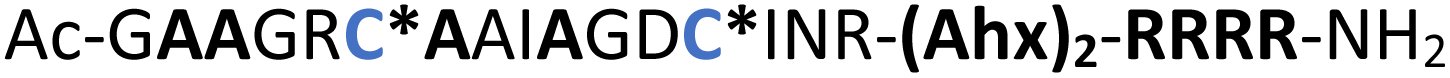

The reactivity of the azobispyrazole cross-linker 8 was initially tested with a model thiol nucleophile to determine the optimal conditions for peptide stapling. The limited aqueous solubility and sensitivity to hydrolysis of cross-linker 8 necessitated the use of anhydrous dimethylformamide (DMF) as the reaction solvent. The reaction of 8 with 2-phenylethanethiol in a mixture of DIPEA in DMF proceeded rapidly at room temperature, with significant conversion observed after 30 minutes (SI, Fig. S1). Applying the cross-linker to the peptide stapling reaction was hindered, however, by the choice of cysteine reducing agent, as commonly used reagents (TCEP, DODT) reacted competitively with 8 to exclusively form side products (SI Fig. S2).^60^ Sodium borohydride was therefore chosen as an alternative reducing agent, enabling successful stapling of peptides 10 and 13 with AzBisPy under anhydrous DMF conditions to obtain peptides 12 and 14, respectively. Peptide 14 was obtained in high purity with 12% yield following preparative HPLC purification and was selected for further characterisation and binding studies, as the poor solubility of peptide 12 limited its application. The dominant Bcl-x_L_ binding residues present in peptide 13 (Val74, Leu78 and Phe81) were mutated to alanine in peptide 15 which was subsequently stapled to AzBisPy to yield peptide 16 as a non-binding control peptide. To compare against our AzBisPy stapled peptides, stapled peptide 11 containing AzB was synthesised using the previously reported azobenzene cross-linker.

### Photochemical characterisation of AzBisPy and Bak^72–87^ stapled peptides

The photochemical properties of the novel azobispyrazole switch 7 were studied in DMSO. Irradiation with 365 nm and 525 nm light displayed excellent *E* → *Z* and *Z* → *E* photoisomerisation with photostationary states (PSSs) featuring >98% *Z* and 96% *E*, respectively (Fig. 2a). The thermal half-life of 7 was determined as 65 days in DMSO at 21 °C. AzBisPy-stapled peptide 14 maintained similar photoswitching, with 97% *Z* and >98% *E* content following 365 nm and 525 nm irradiation in DMSO (Fig. 2b). In the photoswitchable peptide form, the half-life was measured as approximately 181 days (0.5 years) at 21 °C. As *Z* → *E* thermal isomerisation may be impacted by the presence of water,^41,61^ the stability of peptide 14 in its *Z* isomeric state was measured in 10% DMSO in H_2_O at 37 °C to mimic biological conditions (Fig. 2c). Minimal *Z* to *E* thermal isomerisation was observed over 3 days, demonstrating that the stapled peptide could be used to optically modulate PPIs reversibly *in vitro* without competing thermal *Z* → *E* isomerisation. In contrast to the excellent performance of AzBisPy-Bak, AzB-Bak peptide 11 has a previously reported incomplete *E* → *Z* photoisomerisation (76% *Z* at 360 nm PSS) and possesses a greatly reduced half-life of 174 min at just 15 °C.^35^ Finally, the photostability of peptide 14 was measured through repeated 365 nm and 525 nm irradiation between the *E* and *Z* isomers with no significant photodegradation observed (Fig. 2d).

**Fig. 2:**
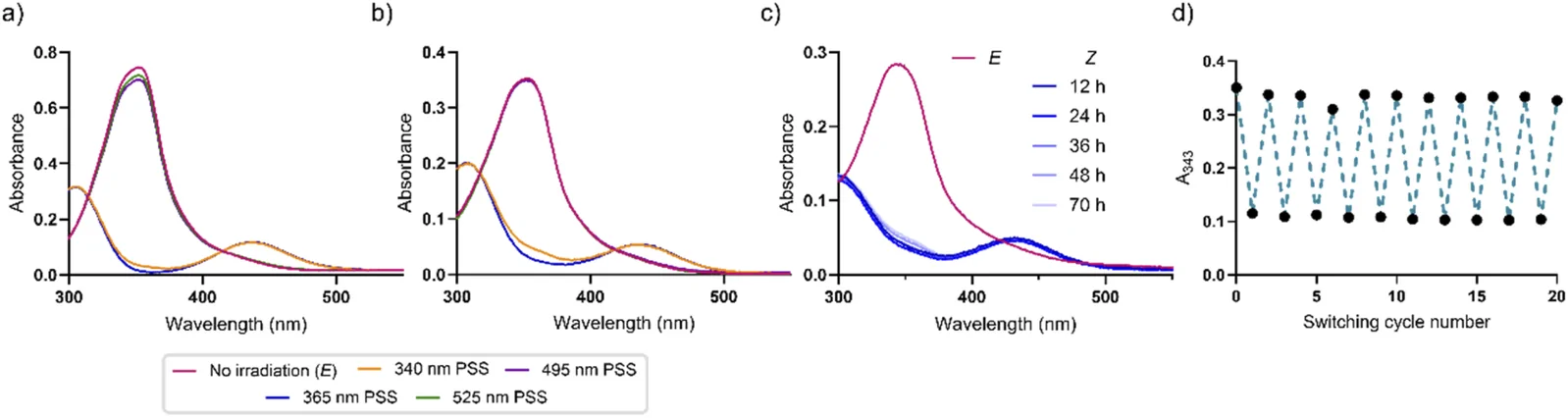
Absorption spectra at concentrations of 15–30 µM of 7 (a) and AzBisPy-stapled peptide 14 (b) in DMSO at 25 °C. The absorption spectra are displayed before irradiation (pink), followed by 365 nm (blue), 525 nm (green), 340 nm (orange) and 495 nm (purple) irradiation to reach the photostationary states (PSS); (c) the thermal half-life of peptide 14 was measured in 10% DMSO in H_2_O at 37 °C with minimal thermal isomerisation observed over 70 hours; (d) photoswitching stability of peptide 14 in 10% DMSO in H_2_O was demonstrated through 10 *E* ↔ *Z* irradiation cycles.

### Conformational change of AzBisPy-Bak^72–87^ upon *E*–*Z* photoisomerisation

Circular dichroism (CD) spectroscopy was used to investigate the effects of photoisomerisation on the secondary structure of the Bak^72–87^ peptides. α-Helical conformations are characterised by distinctive negative ellipticity at 208 nm and 222 nm.^62^ To ensure full peptide solubility, spectra were recorded at a peptide concentration of 25–30 µM in aqueous solutions containing acetonitrile as a cosolvent. The wildtype Bak^72–87^ peptide (9) together with the unstapled peptide analogues (10 and 13) displayed CD spectra characteristic of predominantly disordered conformations, with only weak evidence of α-helical structure (Fig. 3a). Peptide 13 exhibited increased helicity in the presence of TCEP, consistent with reduction of a disulfide bridge formed between the *i*, *i* + 7 cysteine residues in the absence of reducing agent (SI, Fig. S8).

**Fig. 3:**
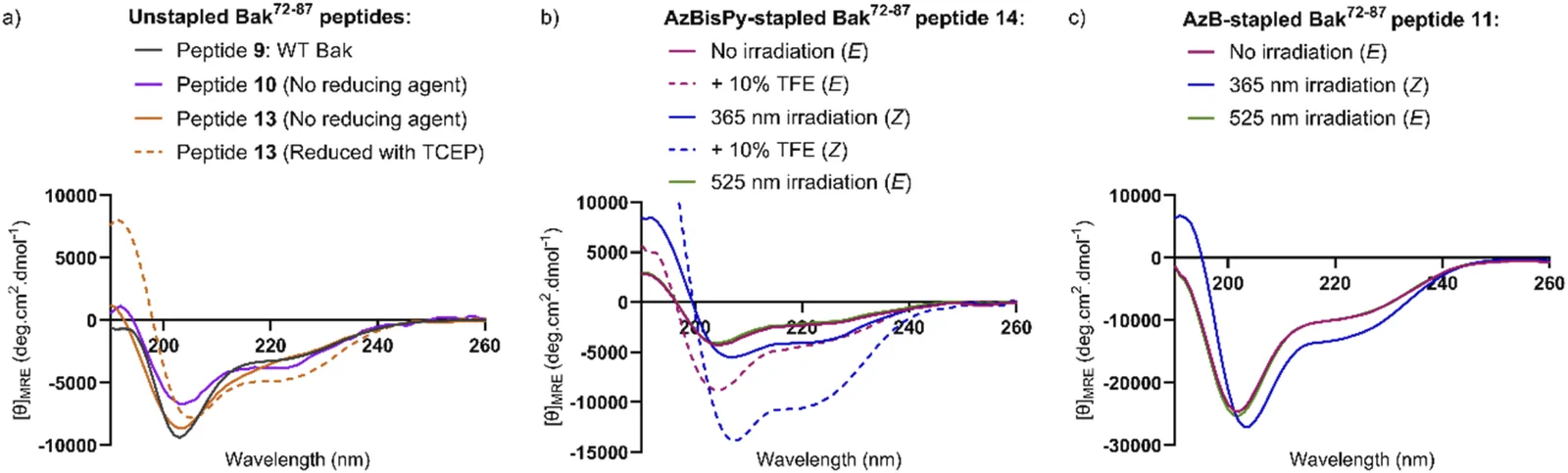
CD spectra of Bak^72–87^ peptides in 25–50% acetonitrile in water: (a) wildtype peptide 9, unstapled peptide 10 (no reducing agent) and unstapled peptide 13 (with and without TCEP reducing agent); (b) AzBisPy-stapled peptide 14 in the pre-irradiated (*E*) state and following 365 nm (*Z*) and subsequent 525 nm irradiation (*E*) (dashed lines display the *E* and *Z* CD spectra in the presence of 10% TFE); (c) AzB-stapled peptide 11 in the pre-irradiated (*E*) state and following 365 nm (*Z*) and subsequent 525 nm irradiation (*E*).

The *E* conformation of AzBisPy-stapled Bak^72–87^14 displayed a spectrum typical of a largely unstructured peptide, with a shallow minimum at 204 nm and minimal negative ellipticity at 222 nm. Following *E* → *Z* photoisomerisation with 365 nm light, the CD spectrum developed a substantially deeper minimum at 207 nm together with increased negative ellipticity at 222 nm, consistent with an increase in α-helical character. Subsequent irradiation at 525 nm regenerated the original spectrum, demonstrating that the conformational change is reversible and directly coupled to the staple photoisomerisation (Fig. 3b). In comparison, the AzB-stapled peptide 11 displayed limited helicity in the *E* state, with a distinct minimum at 202 nm and little ellipticity at 222 nm, confirming the results from Alleman and co-workers (Fig. 3c).^35^ Irradiation with 365 nm light induced a reversible change in the CD spectrum, with the first minimum shifting to 204 nm and the local minimum at 222 nm getting more pronounced. Alleman and coworkers previously proposed that the addition of the helicity-inducing cosolvent 2,2,2-trifluoroethanol (TFE) better approximated the conformation adopted by the AzB-stapled Bak^72–87^ peptide when bound to the Bcl-x_L_ protein.^35^ Consistent with this hypothesis, CD measurements of AzBisPy-stapled Bak^72–87^14 in the presence of 10% TFE showed a significant increase in the *Z*-isomer helicity (Fig. 3b). These results indicate that the *Z* isomer possesses an intrinsically greater propensity to adopt an α-helical conformation, particularly under conditions that favour helix formation.

To understand the structural basis for the distinct helical propensities of the *E* and *Z* isomers of AzBisPy-stapled Bak peptide 14, molecular dynamics (MD) simulations of the two isomers were performed. For comparison, the unstapled Bak peptide 13 was also simulated. Root-mean-square deviation (RMSD)-based clustering of the MD trajectory structures revealed that peptide 13 was largely unstructured, with curled-up and extended structures dominating the conformational ensemble (Fig. 4a). The *E* isomer of peptide 14 was similarly highly unstructured, with the rigid conjugated azobispyrazole linker hindering the formation of folded structures (Fig. 4b). By contrast, the azobispyrazole linker in the *Z* isomer of peptide 14 is more compact and nonplanar, allowing it to adopt mostly helical conformations, with only a small population of partially unfolded states (16%). The partial unfolding was localised to the N-terminal region of the peptide, and this is reflected in the α-helix propensity plot (SI, Fig. S9), which shows high helicities of at least 60% for residues 77–86 and decreased helicities of 20–40% for residues 72–76. Comparatively, the unstapled reduced Bak peptide 13 had substantially lower helicity, while the *E* isomer of peptide 14 showed minimal helical character. These findings are in good agreement with the experimental CD results.

**Fig. 4:**
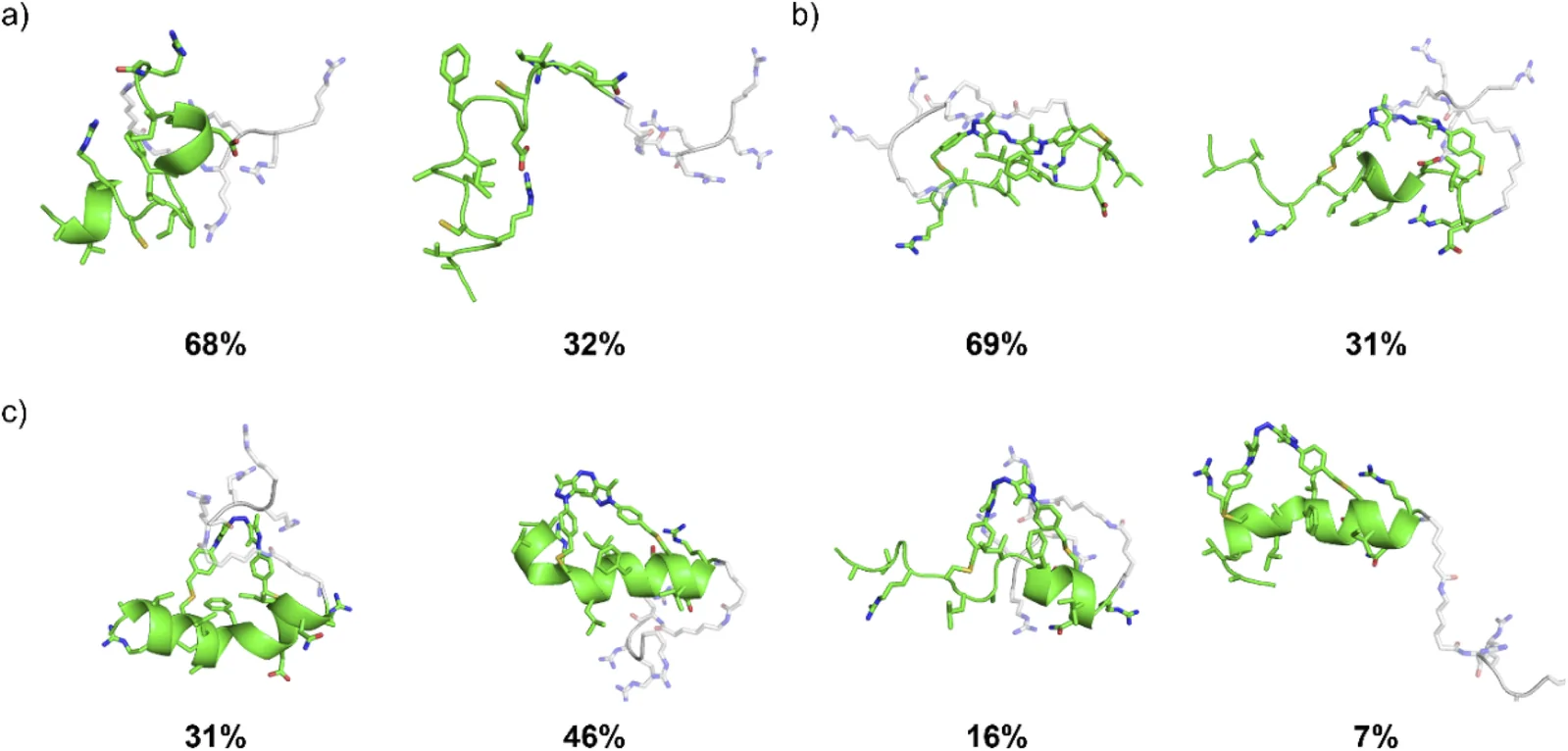
Representative cluster conformations of (a) peptide 13, (b) *E* isomer of peptide 14, and (c) *Z* isomer of peptide 14. The C-terminal poly-Arg tag is shown in white. The percentage populations of the respective conformational clusters are indicated.

### Light-induced binding of AzBisPy-stapled peptides to Bcl-x_L_

To investigate the biological activity of the photoswitch-stapled Bak^72–87^ peptides, fluorescence anisotropy binding experiments with Bcl-x_L_ protein were performed. In initial saturation binding experiments, recombinantly produced Bcl-x_L_^1–209^ (Δ45–84) was titrated against a fixed concentration of N-terminally carboxyfluorescein-labelled Bak^72–87^ wildtype peptide (FAM-9). A *K*_d_ of 1.8 ± 0.4 nM could be determined for the complex (SI, Fig. S10). Based on these results, competition binding experiments with fixed concentrations of fluorescently labelled FAM-9 and Bcl-x_L_ protein and increasing concentrations of Bak^72–87^ peptides were performed to determine the relative peptide binding affinities (SI, Table S1 for full details). For the AzBisPy-stapled Bak peptide 14, binding was measured before irradiation and following 365 nm and then 525 nm irradiation of the assay plate. In addition, the plate was measured up to 48 h post-365 nm irradiation. For the AzB-Bak stapled peptide 11, the plate was measure before irradiation, after 365 nm irradiation, and 13 hours post-irradiation.

The unstapled Bak^72–87^ peptide 10 was unable to fully compete off tracer peptide FAM-9 up to a concentration of 30 µM and displayed a lower binding affinity than the wildtype peptide 9 (Fig. 5a), for which an IC_50_ of 247 ± 78 nM could be determined. The arginine-modified, unstapled control peptide 13, exhibited no binding to Bcl-x_L_ (SI, Fig. S11), unless present in the cysteine-reduced state (IC_50_ = 263 ± 160 nM, Fig. 5b). Prior to irradiation, AzBisPy-stapled peptide 14 displayed no Bcl-x_L_ binding affinity in its *E* isomeric state. Instead, anisotropy increased at high peptide concentrations, indicating potential aggregation of the *E* isomer under the assay conditions (Fig. 5b). However, *E* → *Z* photoisomerisation with 365 nm light switched peptide 14 to a competitor with high binding affinity to Bcl-x_L_ (IC_50_ = 232 ± 84 nM). Subsequent irradiation at 525 nm restored the less helical and weakly binding *E* state, in agreement with what was observed with CD spectroscopy, and demonstrating reversible optical control over Bcl-x_L_ recognition. Importantly, the enhanced affinity of the photoactivated *Z* isomer was maintained for at least 48 h post irradiation (Fig. 5b and SI, S13) indicating that the long thermal lifetime of the AzBisPy*Z* isomer preserves the biologically active state under the assay conditions. The alanine-mutated negative control AzBisPy-stapled peptide 16 displayed no binding to Bcl-x_L_ in the *E* and *Z* isomer (SI, Fig. S14), confirming that the cross-linker is not responsible for the binding affinity of peptide 14.

**Fig. 5:**
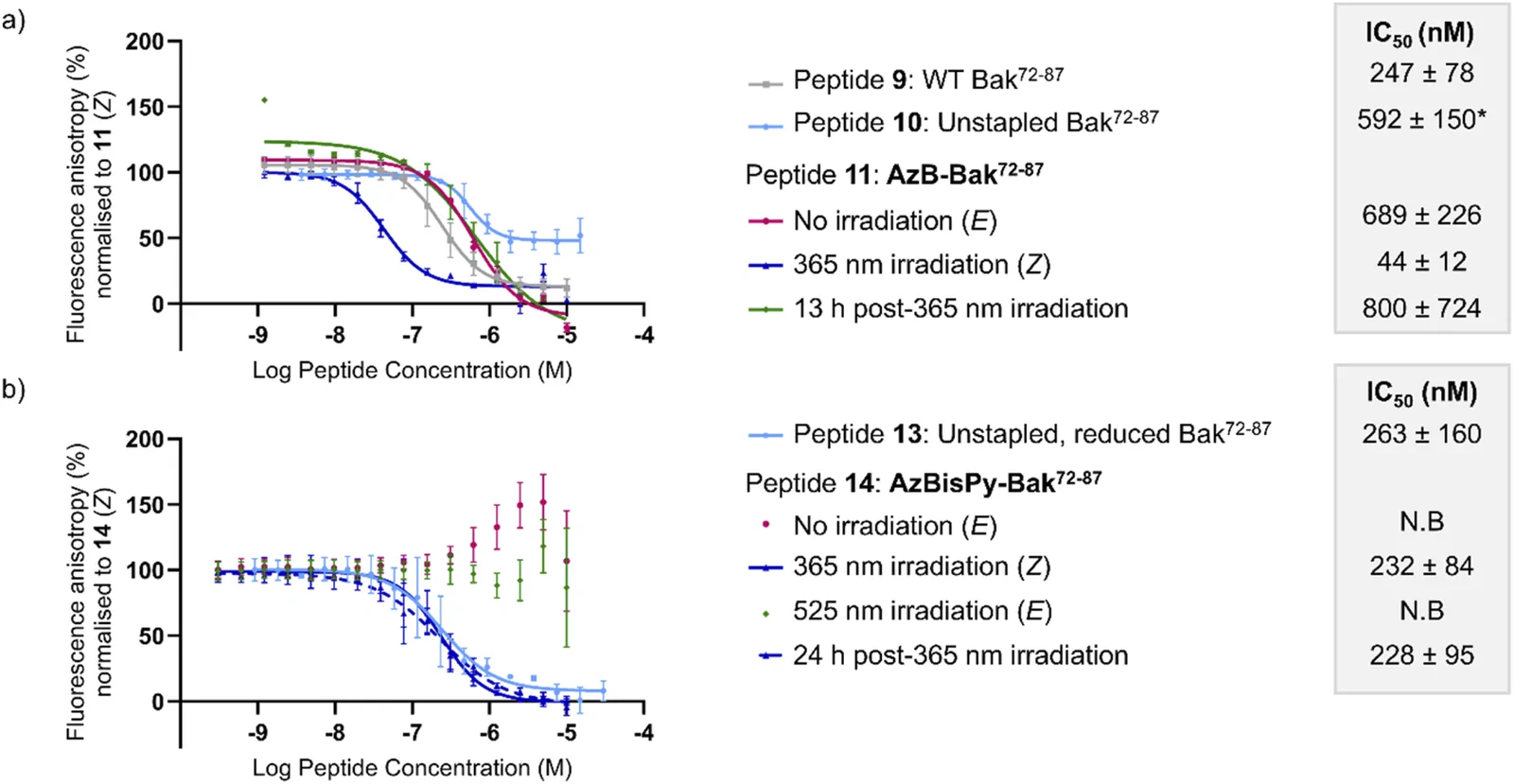
Fluorescence anisotropy competition binding curves for the binding of (a) WT Bak^72–87^ peptide 9, unstapled Bak^72–87^ peptide 10 and AzB-stapled Bak peptide 11 and (b) WT Bak^72–87^ peptide 9, unstapled cysteine-reduced Bak^72–87^ peptide 13 and AzBisPy-stapled Bak peptide 14 to Bcl-x_L_^1–209^ (Δ45–84) protein. The stapled peptide binding affinities to Bcl-x_L_ was determined in the *E*-state before irradiation (pink), after 365 nm plate irradiation (*Z*) (dark blue) and following subsequent 525 nm plate irradiation (*E*) (green) *or* 13–24 h post-365 nm irradiation. All binding curves were normalised to the peptide 11*or*14*Z* isomer. Data points are the mean ± standard error of at least two independent experiments. N.B = no binding observed, * = incomplete tracer displacement observed.

AzB-stapled peptide 11 displayed full competition but low binding affinity (IC_50_ = 689 ± 226 nM) prior to irradiation (Fig. 5a) and could likewise be switched to a high affinity binder (IC_50_ = 44 ± 12 nM) following 365 nm irradiation as previously reported by Alleman and co-workers.^35^ Key differences in the photoswitchable peptide designs could be observed when the binding affinity of 11 was re-determined 13 hours post-365 nm irradiation. The AzB-stapled peptide 11 reverted to its low-affinity state, indicating rapid *Z* → *E* thermal relaxation of the AzB staple and corresponding return to a peptide with low helical content. In contrast, the AzBisPy-staple analogue 14 remained in its high-affinity *Z* state over 24 hours, highlighting the greatly extended *Z*-isomer lifetime of the AzBisPy staple.

### Application of the AzBisPy-stapling to another PPI

To demonstrate that this cross-linker could be broadly applied to modulate PPIs with light, AzBisPy was stapled to a modified p53 peptide designed to target MDM2. The p53/MDM2 interaction is another helically mediated PPI implicated in cancer and MDM2 has been targeted previously with azobenzene-PMI stapled peptides.^63,64^ The transcription factor p53 functions as a tumour suppressor and its negative regulator, MDM2, displays increased expression in a variety of cancers.^65^ The WT p53^17–28^ peptide 17 was modified to peptide 19 to incorporate *i*, *i* + 7 cysteines through the replacement of Asp21 and Glu28 (Table 2). In addition, the Pro27 residue, which has been shown to disrupt helical structure and impair binding, was replaced with a serine.^66^

**Table 2:** Sequences of synthesised p53 peptides. Ac: acetyl; Flu: fluorescein-5-maleimide; * denotes stapling at *i*, *i* + 7 cysteine residues with an azobispyrazole cross-linker (AzBisPy)

	Peptide	Sequence
17	WT p53^17–28^	Ac-ETFSDLWKLLPE-NH_2_
Flu-18	Flu-[C^32^]-p53^15–32^	Ac-SQETFSDLWKLLPENNVC^Flu^-NH_2_
19	[C^21,28^,S^27^]-p53^17–28^	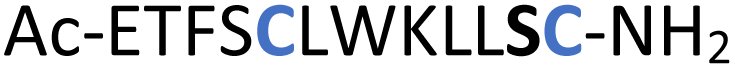
20	[C^21,28^,S^27^]-p53^17–28^ – AzBisPy	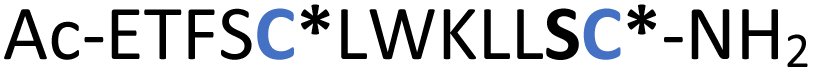

The stapling conditions developed for Bak were applied to p53^17–28^ peptide 19. Once stapled to AzBisPy, the binding of AzBisPy-p53^17–28^20 to MDM2 was measured through displacement of a fluorescently-labelled p53^15–32^ peptide 18. Wild-type p53^17–28^ peptide 17 displayed binding to MDM2 with an IC_50_ of 1180 ± 40, whereas only partial binding was observed with the *i*, *i* + 7 cysteine-modified unstapled peptide 19. The irradiation of the photoswitchable AzBisPy-p53^17–28^ peptide 20 was carried out prior to addition to the assay plate, as MDM2 degradation was observed upon *in situ* irradiation. Peptide 20 in the *E* conformation exhibited low-affinity binding (Fig. 6). Consistent with our previous results, the *Z* isomer of 20 displayed improved binding affinity to MDM2 with an IC_50_ of 234 ± 42 nM, demonstrating the ability of the AzBisPy cross-linker to modulate the conformation and binding of other peptides with light.

**Fig. 6:**
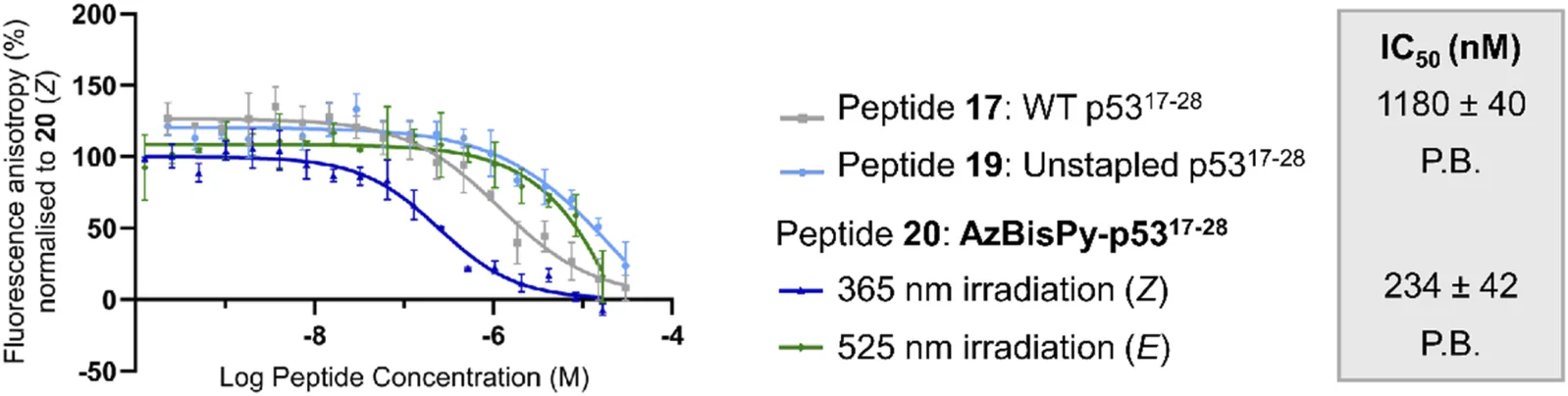
Fluorescence anisotropy competition binding curves for the binding of WT p53^17–28^ peptide 17, unstapled p53^17–28^ peptide 19 and AzBisPy-stapled p53 peptide 20 to MDM2^17–125^ protein. Peptide 20 was measured in the 365 nm photostationary state and 525 nm photostationary state, with irradiation of the sample carried out prior to the addition to the protein. All binding curves were normalised to the peptide 20*Z* isomer. Data points are the mean ± standard error. P.B = partial binding observed.

## Conclusion

AzBisPy represents the first heteroaromatic, photoswitchable peptide staple. In this work, a synthesis route was developed to functionalise an azobispyrazole switch to enable peptide stapling of cysteines at *i*, *i* + 7 positions. The azobispyrazole cross-linker exhibited robust photochemical properties with excellent *E* ↔ *Z* photoconversion and long thermal half-life. A stapling strategy was developed to generate AzBisPy-Bak and p53 stapled peptides that inhibit Bcl-x_L_ and MDM2, respectively, upon photoisomerisation to the more helical, bioactive *Z* conformation. The near-quantitative, bidirectional switching and exceptional *Z*-isomer stability represent a major improvement over previous designs, enabling long-lived Bcl-x_L_ binding with highly precise photocontrol. This azobispyrazole cross-linker can therefore be applied to develop future peptide-based PPI modulators in which a high degree of spatiotemporal control is required. Whilst further work is required to achieve red light switching, the expansion on previous photoswitchable stapled peptide designs creates further opportunities to use light as a tool to probe PPIs *in cellulo* and as a potential photoresponsive peptide therapeutic.

## Author contributions

D. P., A. B. and M. F. conceived and supervised the project. S. M., K. S., D. P., A. T., D. W., Y. T. and M. M. performed the experiments. S. M., K. S., D. P., A. T., D. W., Y. T., A. B. and M. F. contributed to data analysis. S. M., D. P., K. S., A. T., D. W., Y. T., A. B. and M. F. wrote the manuscript and contributed to manuscript editing.

## Conflicts of interest

There are no conflicts to declare.

## Supplementary Material

SC-OLF-D6SC06137K-s001

## Data Availability

The synthesis and characterisation of AzBisPy and stapled peptides is described in the accompanying supplementary information (SI). A previously reported protocol for the synthesis of AzB was utilised.^31^ Raw experimental data, NMR, UV-Vis, HPLC and mass spectrometry data, where relevant, have been uploaded to a data repository hosted by Imperial College London and can be accessed *via* the following URL: https://doi.org/10.82186/vph6w-x9b57. Supplementary information is available. See DOI: https://doi.org/10.1039/d6sc06137k.
