# Supplementary material for: Long-lived azobispyrazole stapled peptides for light-activated inhibition of protein–protein interactions
Source: Chem Sci. 2026 Nov 2. Online ahead of print. doi: 10.1039/d6sc06137k (PMC13733666; doi:10.1039/d6sc06137k)
Supplement: Supplementary file 1 [file SC-OLF-D6SC06137K-s001.pdf]

Supplementary Information (SI) for Chemical Science.
This journal is © The Royal Society of Chemistry

Supporting Information

Long-Lived Azobispyrazole Stapled Peptides for Light-Activated Inhibition of Protein-Protein Interactions

Sinead McCann,[a] Davia Prischich,[b]† Kira Scobel,[a]† Dennis J. Worm,[a] Aditya R. Thawani,[a] Marie Mori,[a] Yaw Sing Tan,[c] Anna Barnard*[a] and Matthew J. Fuchter*[b]

a. Department of Chemistry, Molecular Sciences Research Hub, Imperial College London, 82 Wood Lane, London W12 0BZ, United Kingdom
b. Department of Chemistry, University of Oxford, 12 Mansfield Road, Oxford, OX1 3TA, United Kingdom
c. Bioinformatics Institute, Agency for Science, Technology and Research (A*STAR), 30 Biopolis Street, #07-01 Matrix, Singapore 138671, Singapore

† These authors contributed equally.

Correspondence to: Anna Barnard (a.barnard@imperial.ac.uk) and Matthew J. Fuchter (matthew.fuchter@chem.ox.ac.uk)

Table of Contents

Supplementary Figures and Data 3

Experimental Procedures 11

Materials and general methods 11

Circular dichroism (CD) spectroscopy 12

UV-Vis spectroscopy 12

Fluorescence anisotropy 12

Peptide Synthesis 14

Solid-phase peptide synthesis (SPPS) 14

Peptide N-acetylation and cleavage 14

Peptide purification 14

Peptide stapling 14

Peptide characterisation 20

Protein expression 27

Compound preparation and characterisation 29

3,5-dimethyl-1*H*-pyrazol-4-amine (2) 29

3-(2-(3,5-dimethyl-1*H*-pyrazol-4-yl)hydrazineylidene)pentane-2,4-dione (3) 29

(*E*)-1,2-bis(3,5-dimethyl-1*H*-pyrazol-4-yl)diazene (4) 30

(4-(((*tert*-butyldimethylsilyl)oxy)methyl)phenyl)boronic acid (5) 30

(*E*)-1,2-bis(1-(4-(((tert-butyldimethylsilyl)oxy)methyl)phenyl)-3,5-dimethyl-1*H*-pyrazol-4-yl)diazene (6) 30

(*E*)-((diazene-1,2-diylbis(3,5-dimethyl-1*H*-pyrazole-4,1-diyl))bis(4,1-phenylene))dimethanol (7) 31

(*E*)-1,2-bis(1-(4-(bromomethyl)phenyl)-3,5-dimethyl-1*H*-pyrazol-4-yl)diazene (8) 31

Molecular Dynamics 31

NMR Spectra 33

References 39

Supplementary Figures and Data

AzBisPy 8 Reactivity Experiments

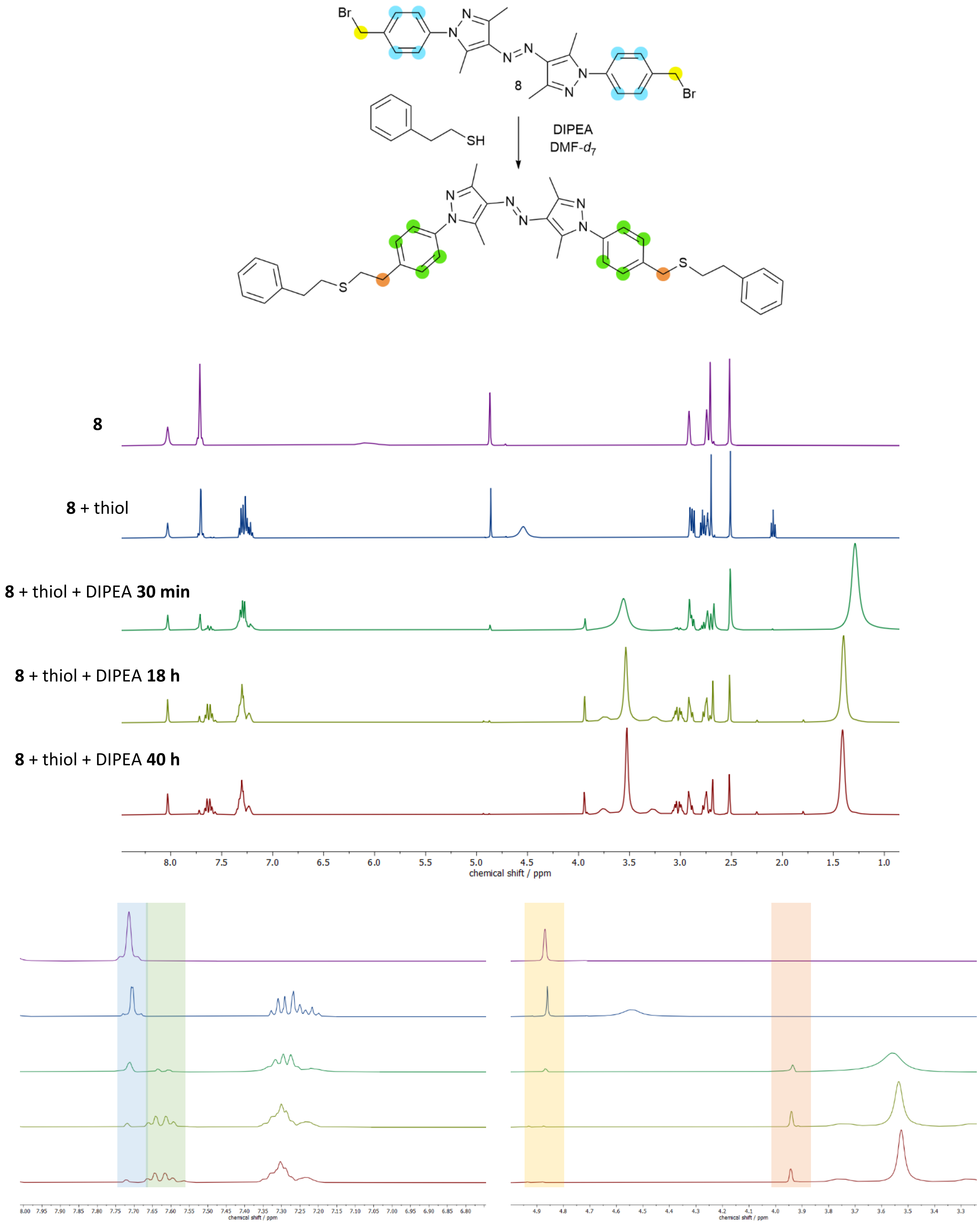

Figure S1: Reaction of **8** with 2-phenylethanethiol, DIPEA in DMF-d_7 monitored by ^{1}H NMR after 30 minutes, 18 h and 40 h with zoomed ^{1}H NMR sections with significant peaks highlighted.

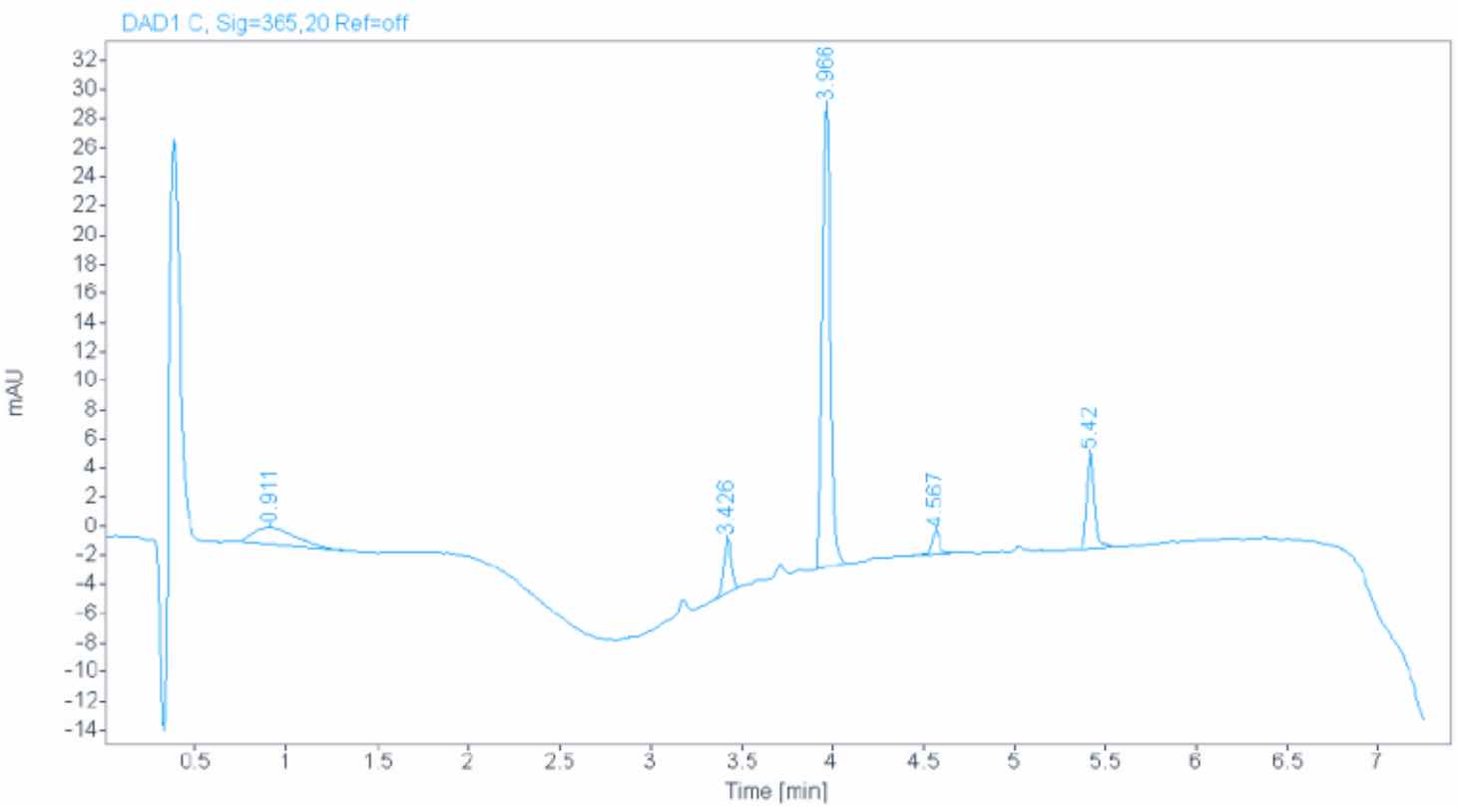

Peak RT 3.470

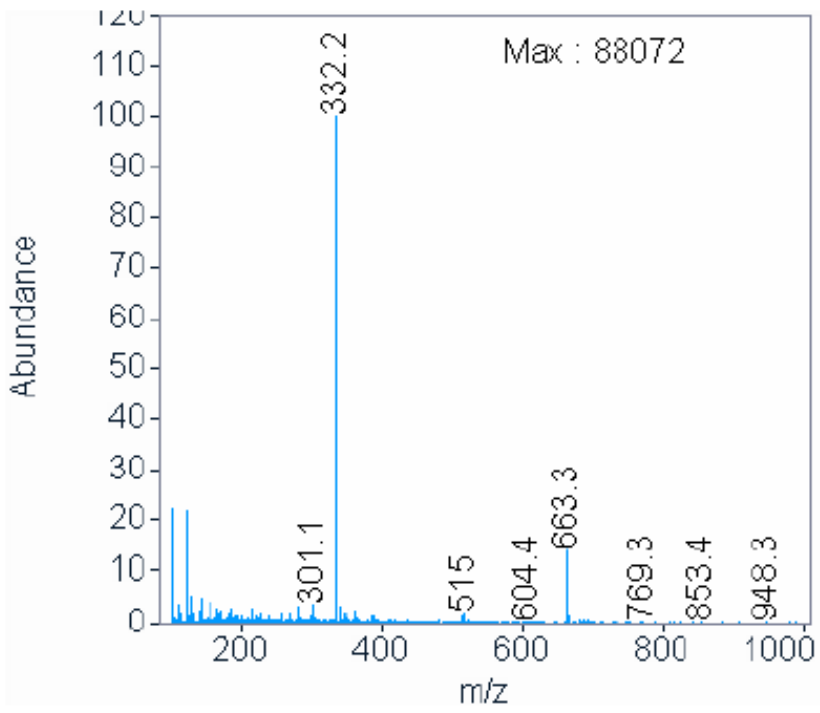

Peak RT 4.011

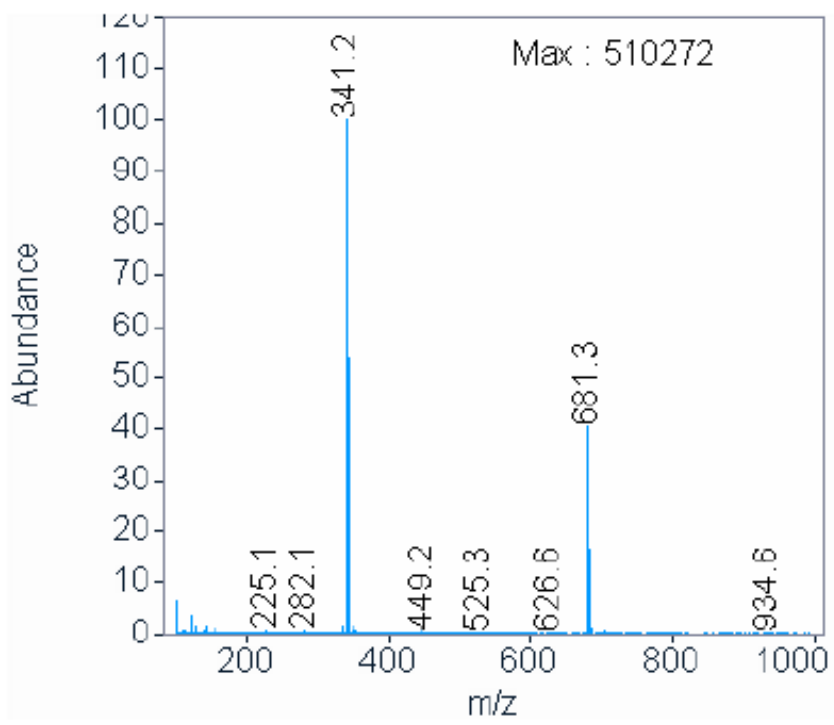

Peak RT 5.439

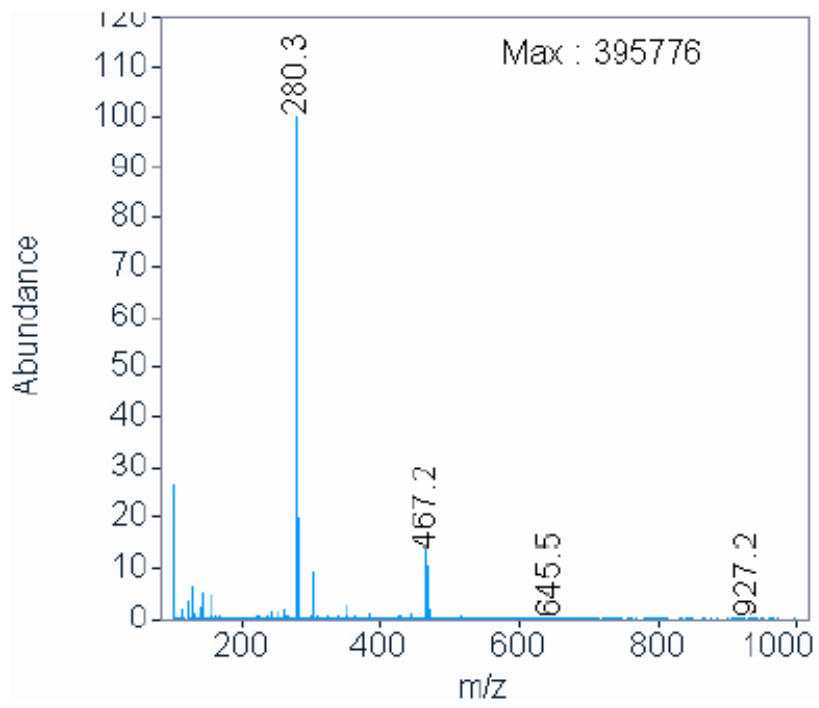

Exact Mass: 662.26

Exact Mass: 680.23

Exact Mass: 466.14

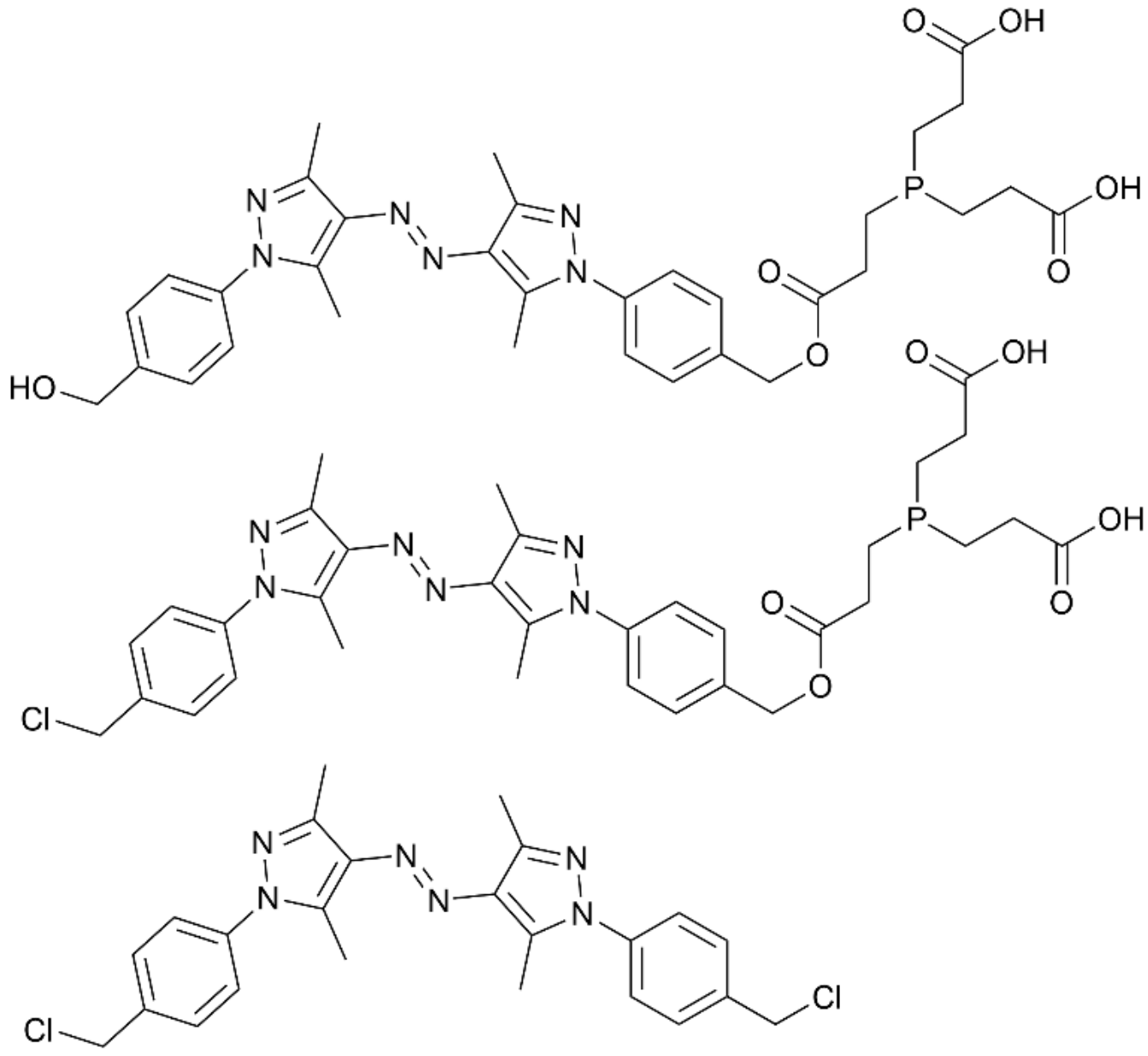

Figure S2: LCMS of **8** in stapling conditions containing TCEP.HCl, DIPEA in DMF. Complete consumption of **8** to the side products depicted was observed.[1]

Photostationary State (PSS)

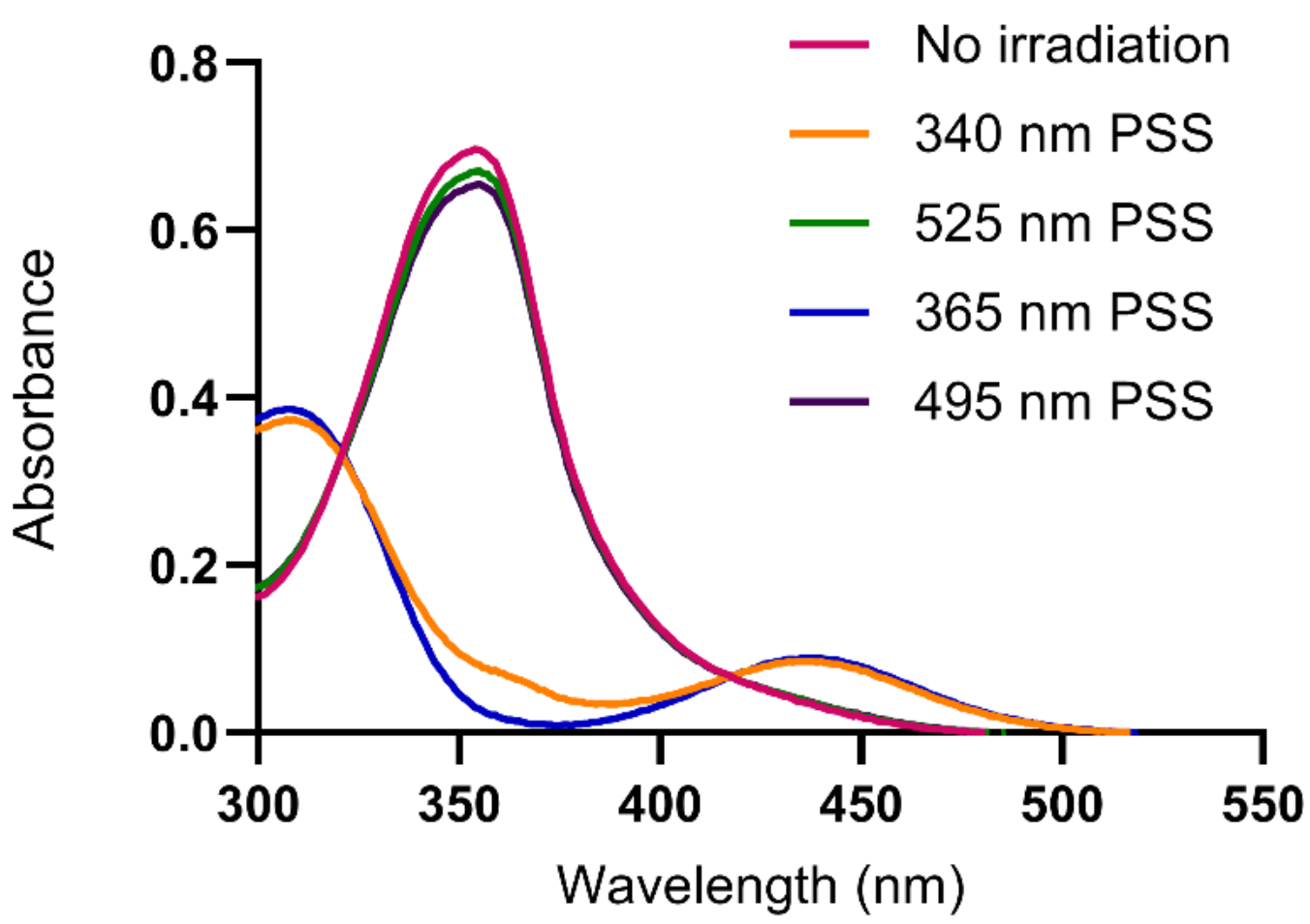

Figure S3: Absorption spectra of AzBisPy **8** in the non-irradiated state and the photostationary states (PSS) after irradiation with 340 nm (92% *Z*), 365 nm (>98% *Z*), 495 nm (94% *E*) and 525 nm (96% *E*) light at 25 °C in DMSO.

Thermal isomerisation

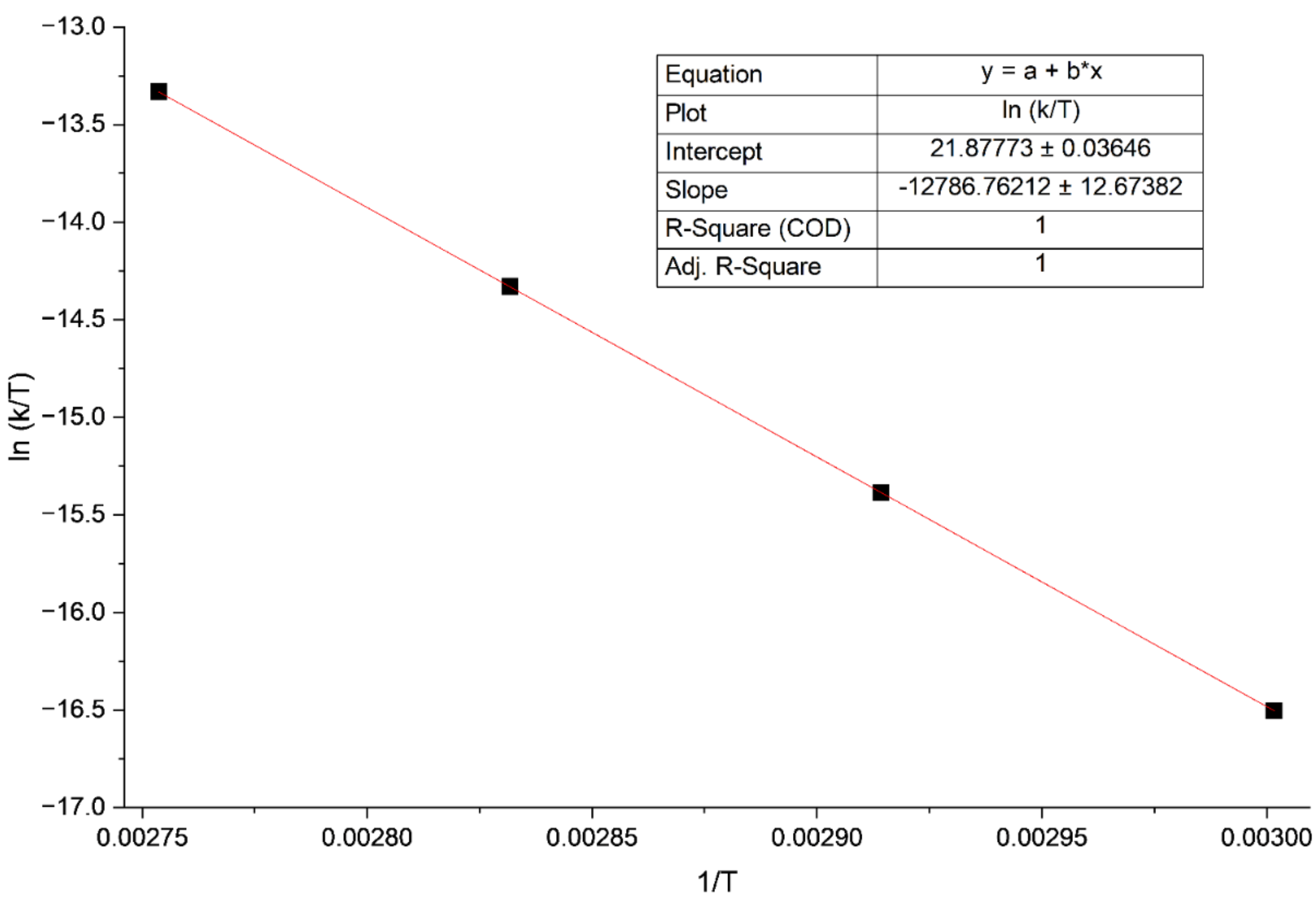

Figure S4: Eyring kinetic plot for **7** as determined in DMSO obtained through thermal isomerisation measurements at four different temperatures (60, 70, 80 and 90 °C). At 294.15 K (21 °C) a half-life of 65 days could be extrapolated.

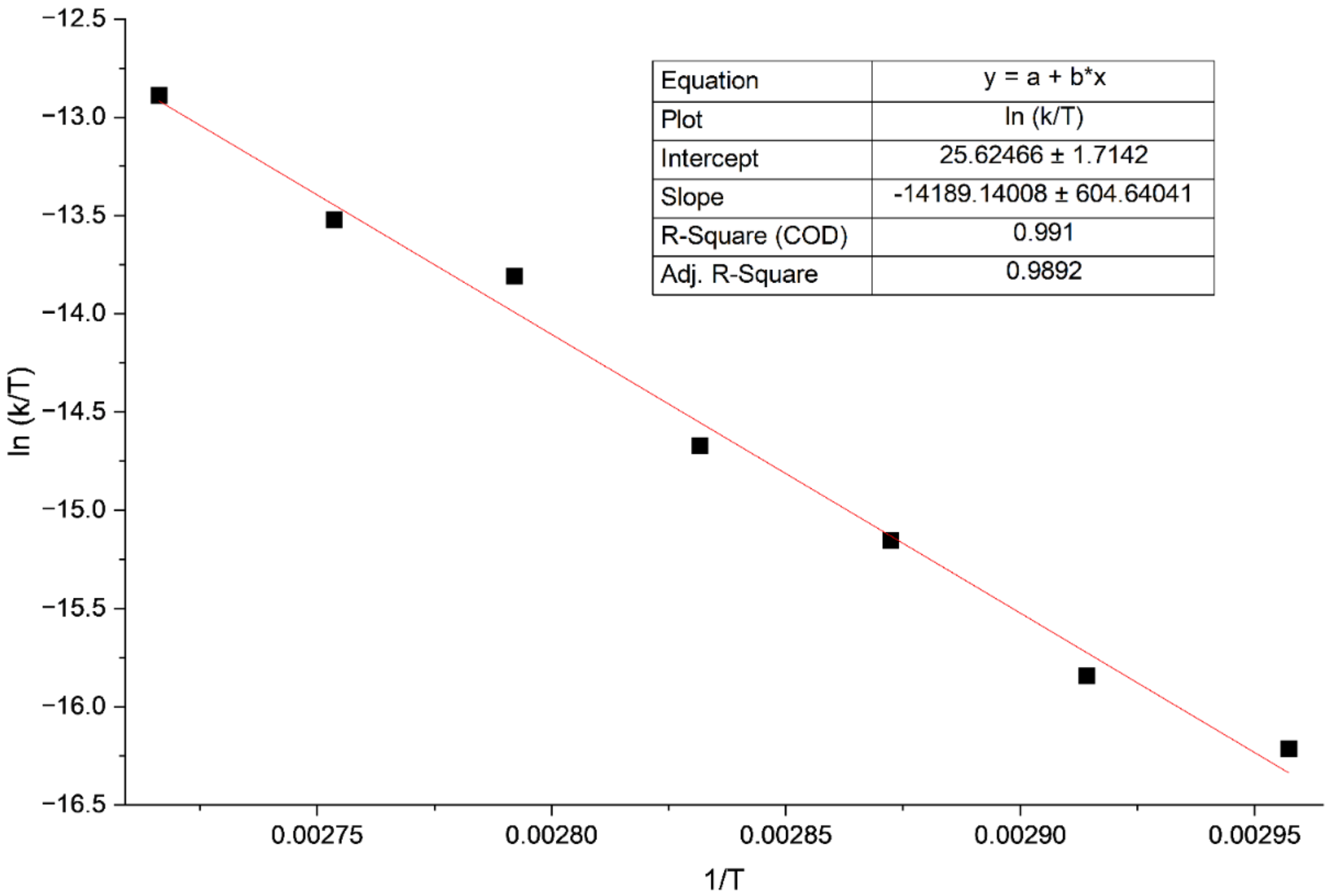

Figure S5: Eyring kinetic plot for peptide **14** as determined in DMSO obtained through thermal isomerisation measurements at seven different temperatures (65, 70, 75, 80, 85, 90 and 95 ˚C). At 294.15 K (21 °C) and 310.15 K (37 °C), a half-life of 181 and 14 days respectively could be extrapolated.

Molar extinction coefficient

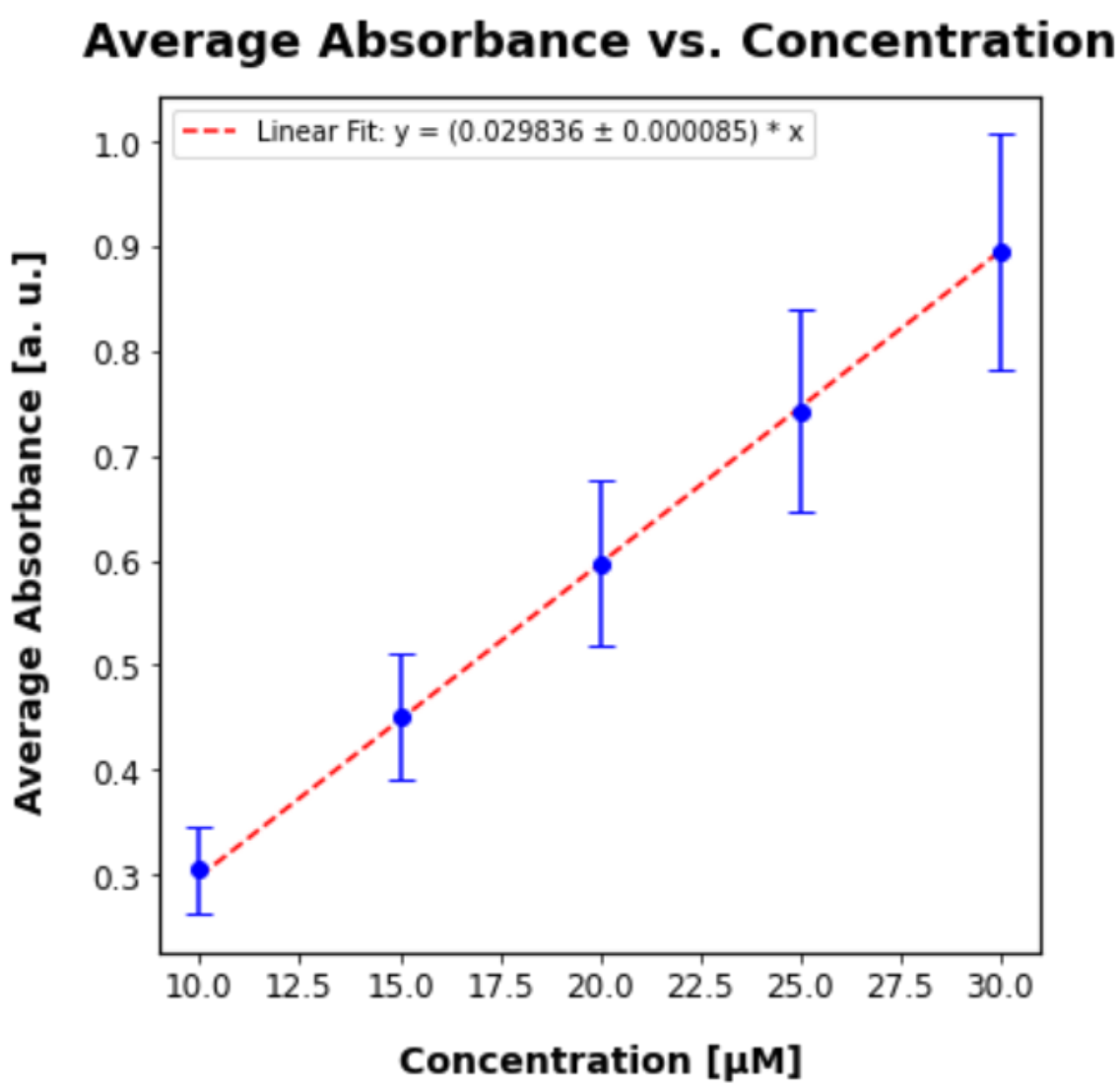

Figure S6: Plot of the maximum absorbance of AzBisPy **8** at concentrations ranging from 10 μM to 30 μM. Three repeats were averaged for each concentration. Molar extinction determined as 29836 mol L^{-1} cm^{-1} at 354 nm.

Circular Dichroism Spectroscopy

a)

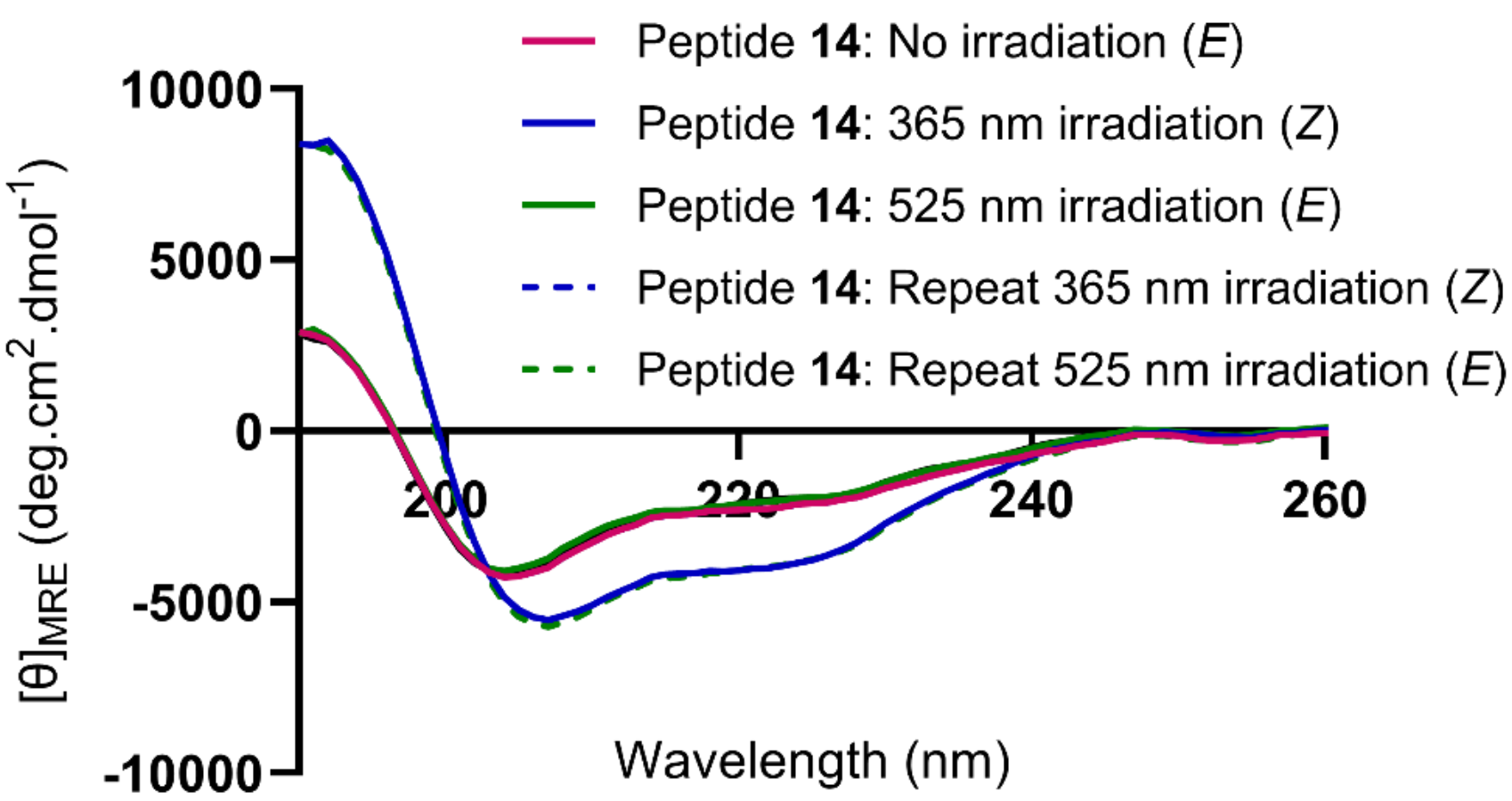

b)

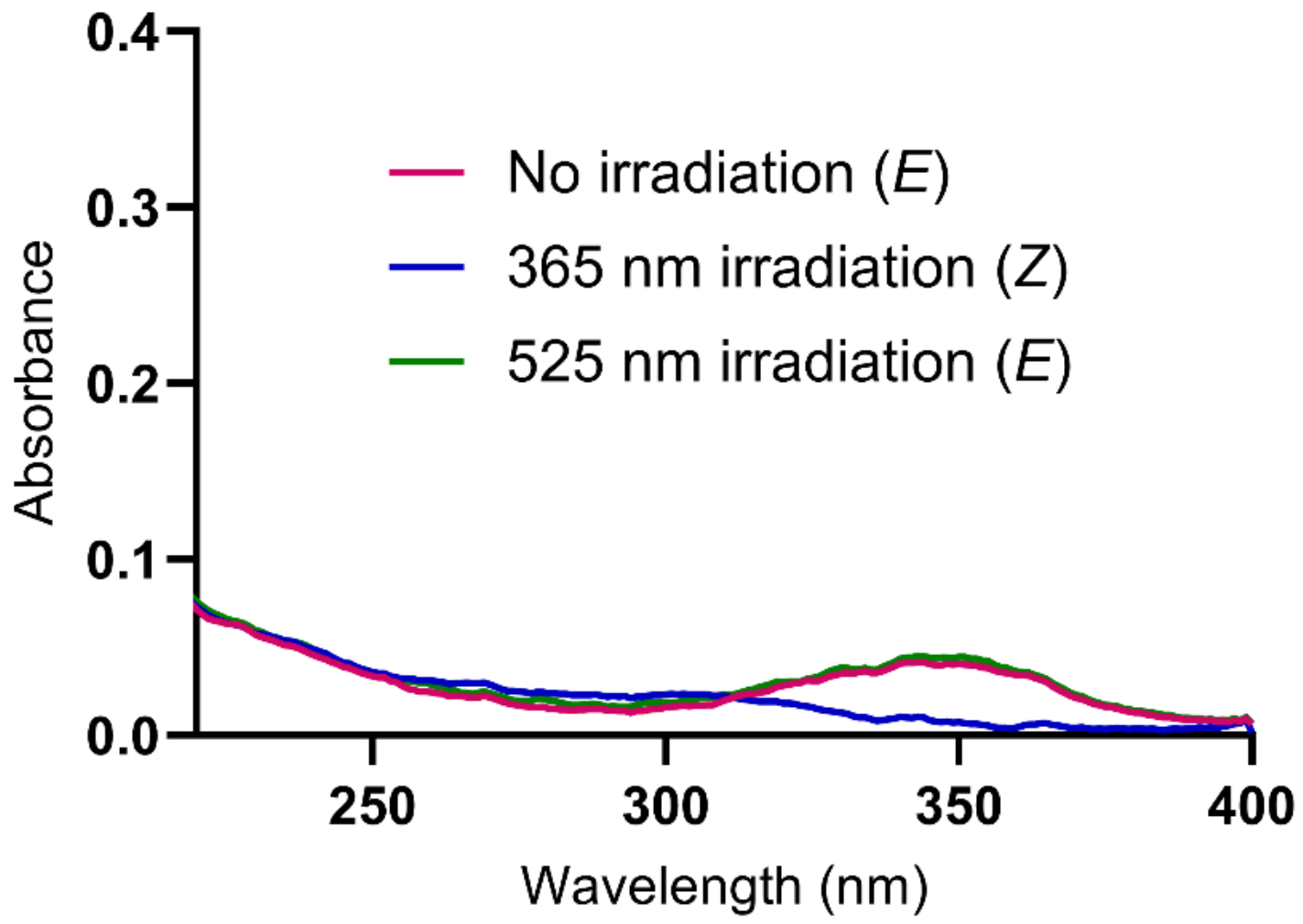

Figure S7: a) CD spectra of peptide **14** in 50% MeCN in H_2O in the pre-irradiated (*E*) state (pink), after 365 nm irradiation (*Z*) (blue) and following 525 nm irradiation (*E*) (green). The second irradiation cycle is depicted in dashed lines. **b)** Absorbance plot measured on the Chirascan V100 CD spectrometer during the CD scan of peptide 14 in 50% MeCN in H_2O in the pre-irradiated (*E*) state (pink), after 365 nm irradiation (*Z*) (blue) and following 525 nm irradiation (*E*) (green) confirming the CD change corresponds to the *E*-*Z* photoisomerisation.

Peptide 13 HPLC characterisation with and without TCEP

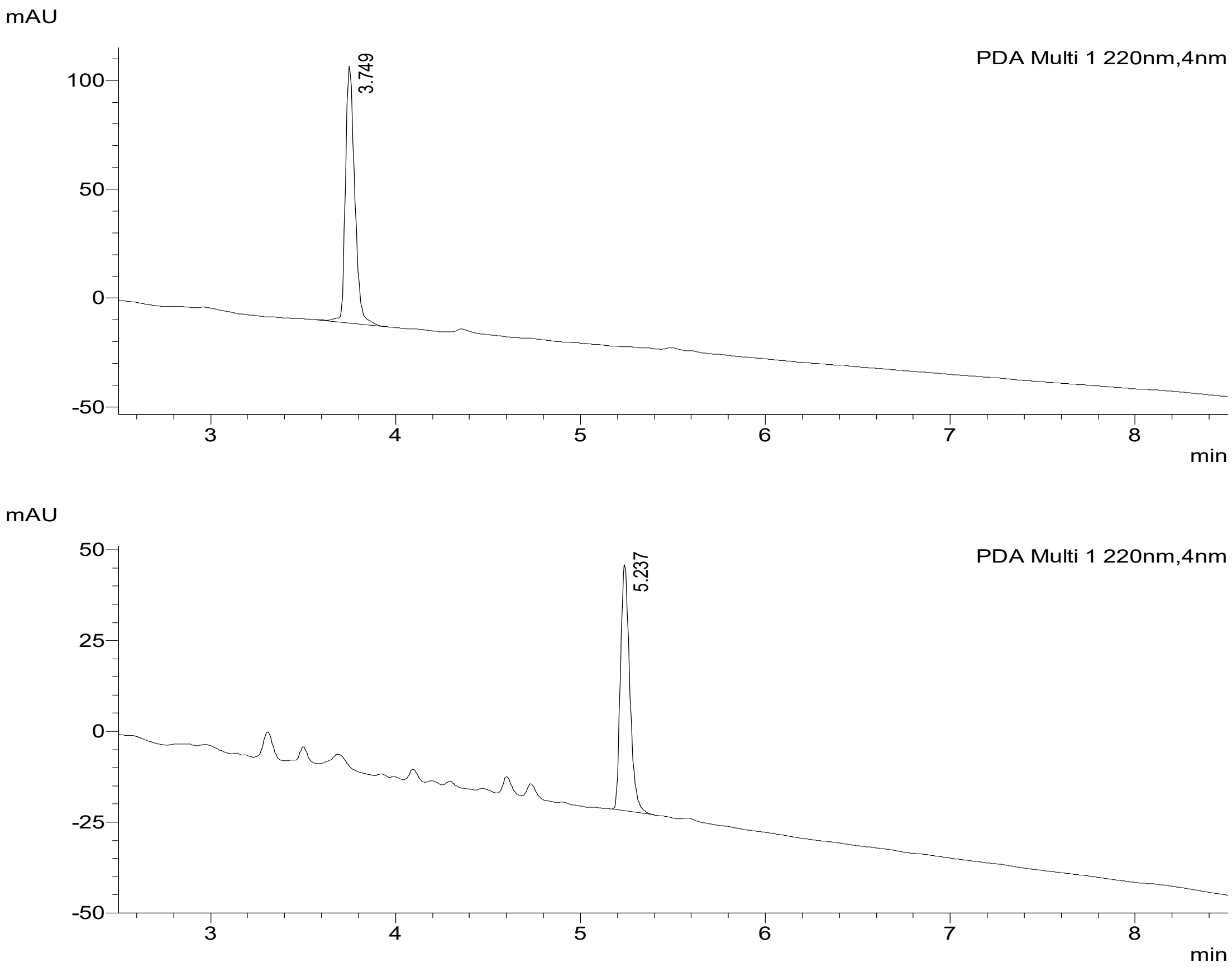

Figure S8: Peptide **13** ([A^{73},F^{81},C^{77,84}]-Bak$^{72\text{-}87}$-Arg tag) – 3.749 min retention time without reducing agent, 5.237 min retention time of the same solution when reduced with TCEP

Alpha-Helix Propensity Plot

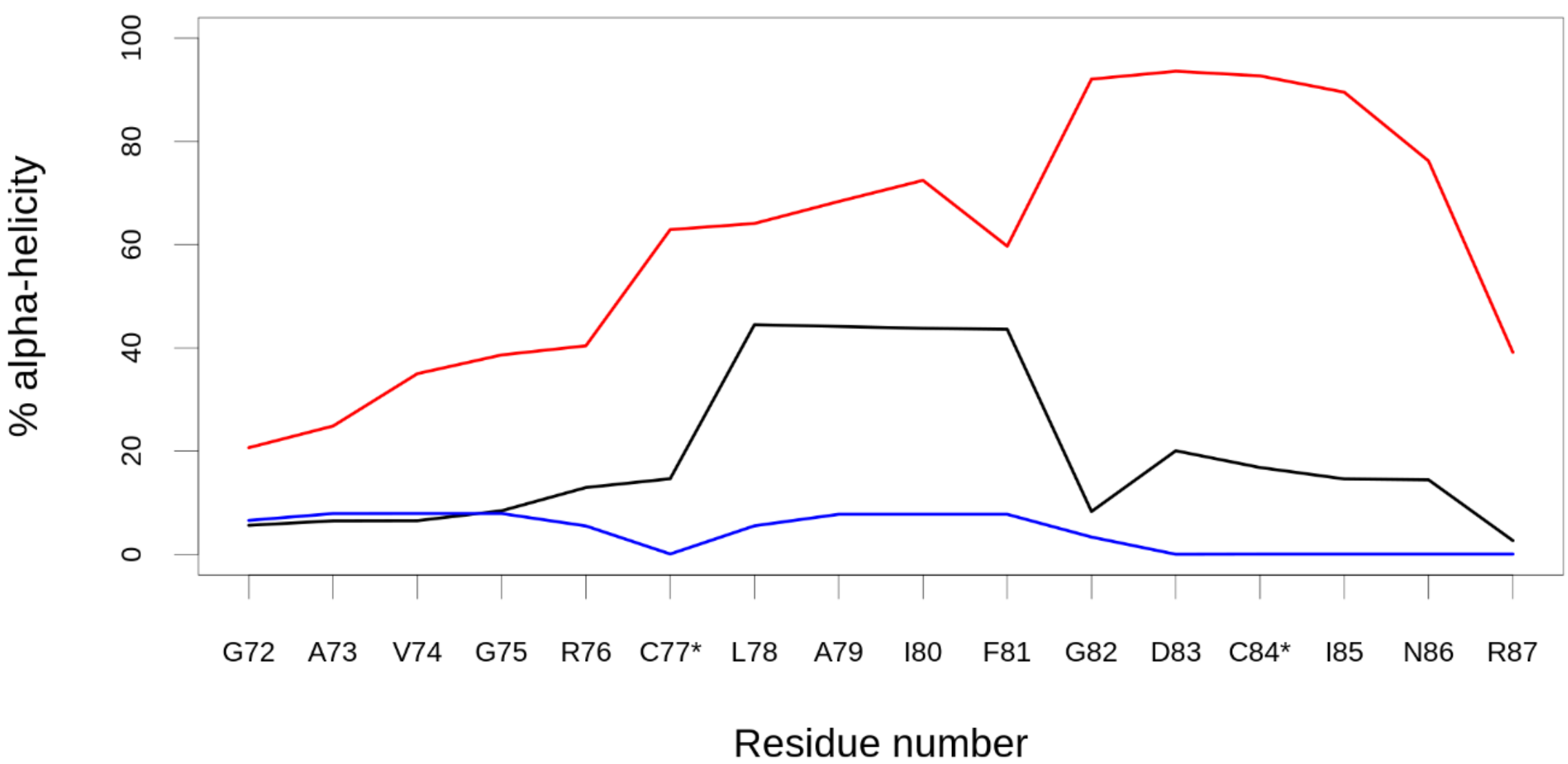

Figure S9: α-helix propensity of residues in peptide **13** (black), *E* isomer of peptide **14** (blue), and *Z* isomer of peptide **14** (red) during the final 300 ns of MD simulations. Cysteine residues 77 and 84 are stapled in peptide **14** and indicated with *.

Fluorescence anisotropy

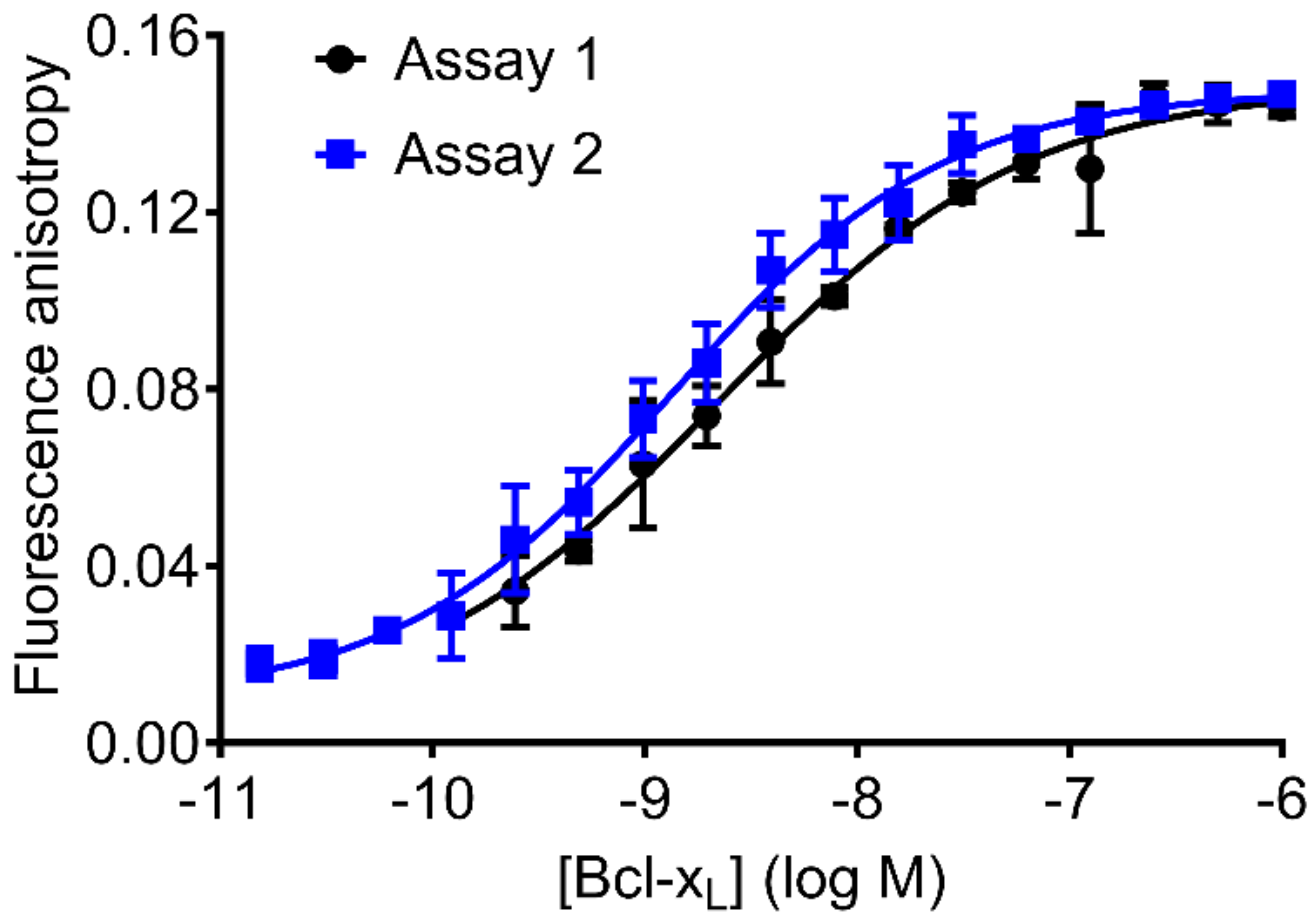

Figure S10: Fluorescence anisotropy saturation binding curves for the binding of FAM-Bak$^{72\text{-}87}$ (peptide **FAM-9**) to Bcl-$x_L^{1\text{-}209}$(Δ45-84) protein. Error bars represent the standard deviation from triplicate values for each tested concentration. An average K_d of 1.8 ± 0.4 nM for the complex was determined from the two shown independent experiments.

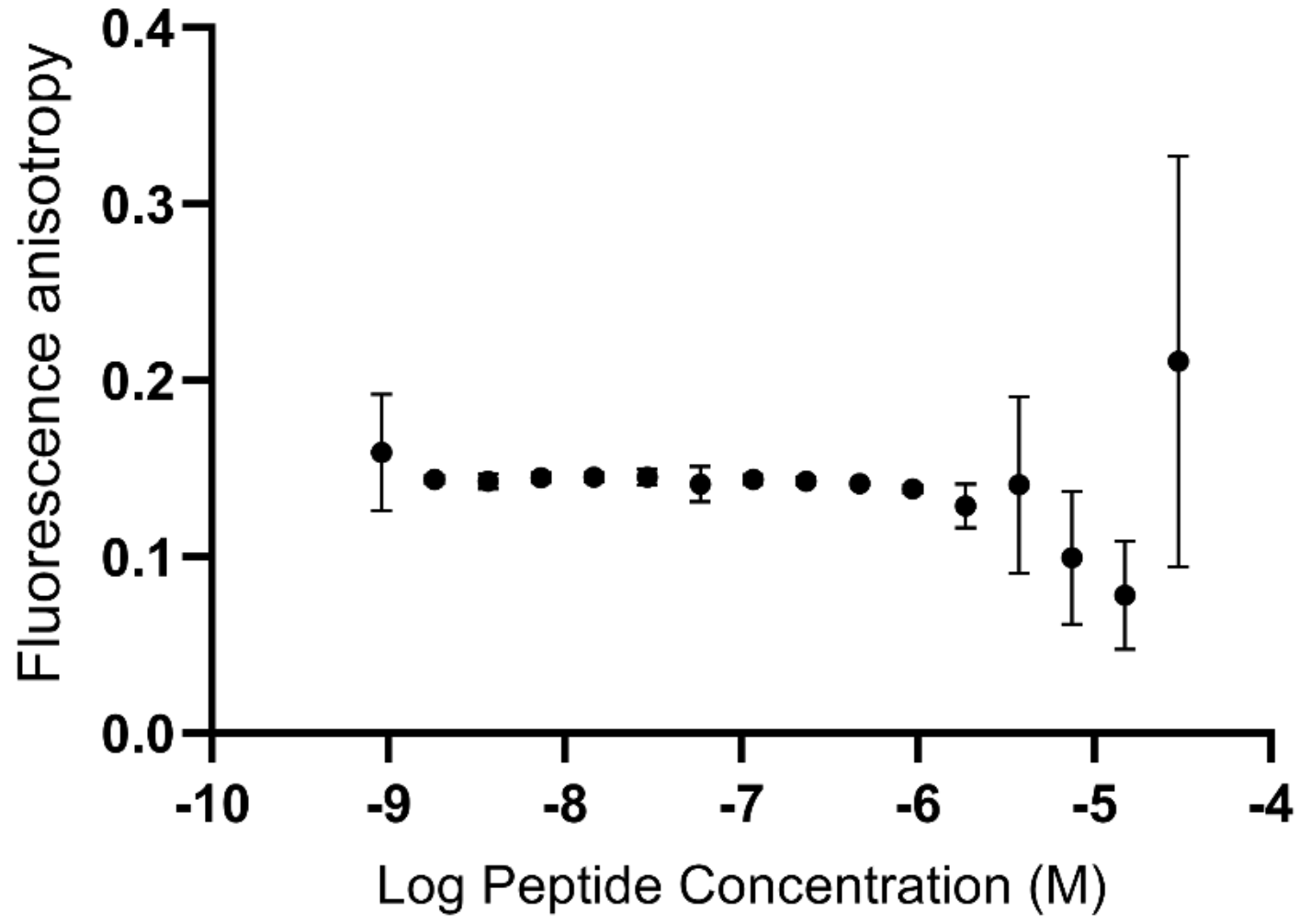

Figure S11: Fluorescence anisotropy competition binding curve for the binding of peptide **13** (without pre-reduction with TCEP) to Bcl $x_L^{1\text{-}209}$(Δ45 84) protein.

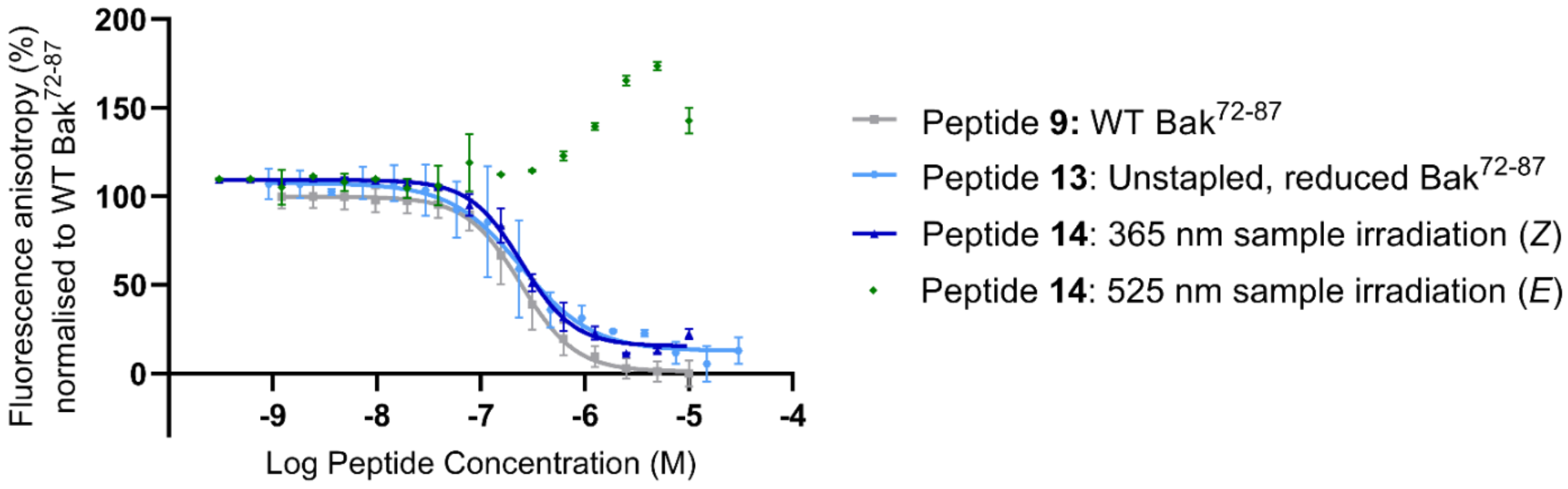

Figure S12: Fluorescence anisotropy competition binding curve for the binding of peptide **9, 13, 14 (365 nm PSS)** and **14 (525 nm PSS)** to Bcl-x$_L$ protein$^{1-209}$(Δ45 84) with samples of peptide **14** irradiated prior to addition to the plate.

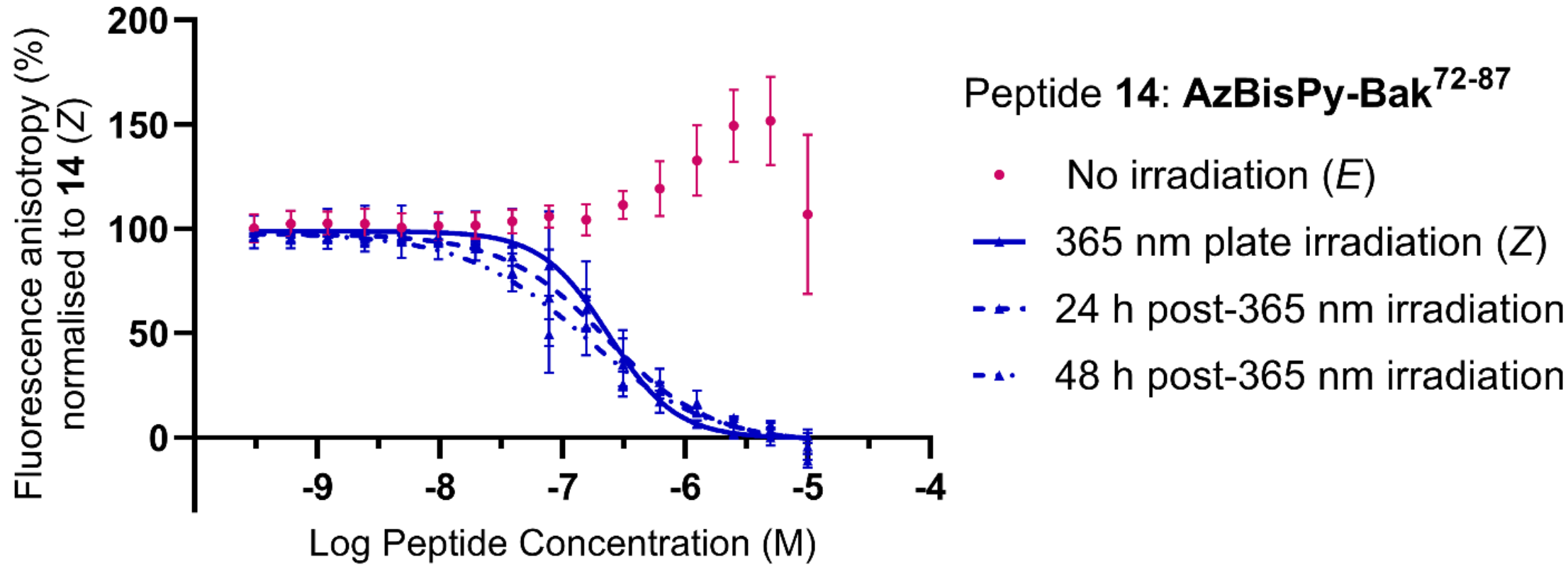

Figure S13: Fluorescence anisotropy competition binding curve for the binding of peptide **14** to Bcl-x$_L$ protein$^{1-209}$(Δ45 84). The plate was measured before irradiation, after 365 nm plate irradiation, 24 h and 48 h-post 365 nm irradiation.

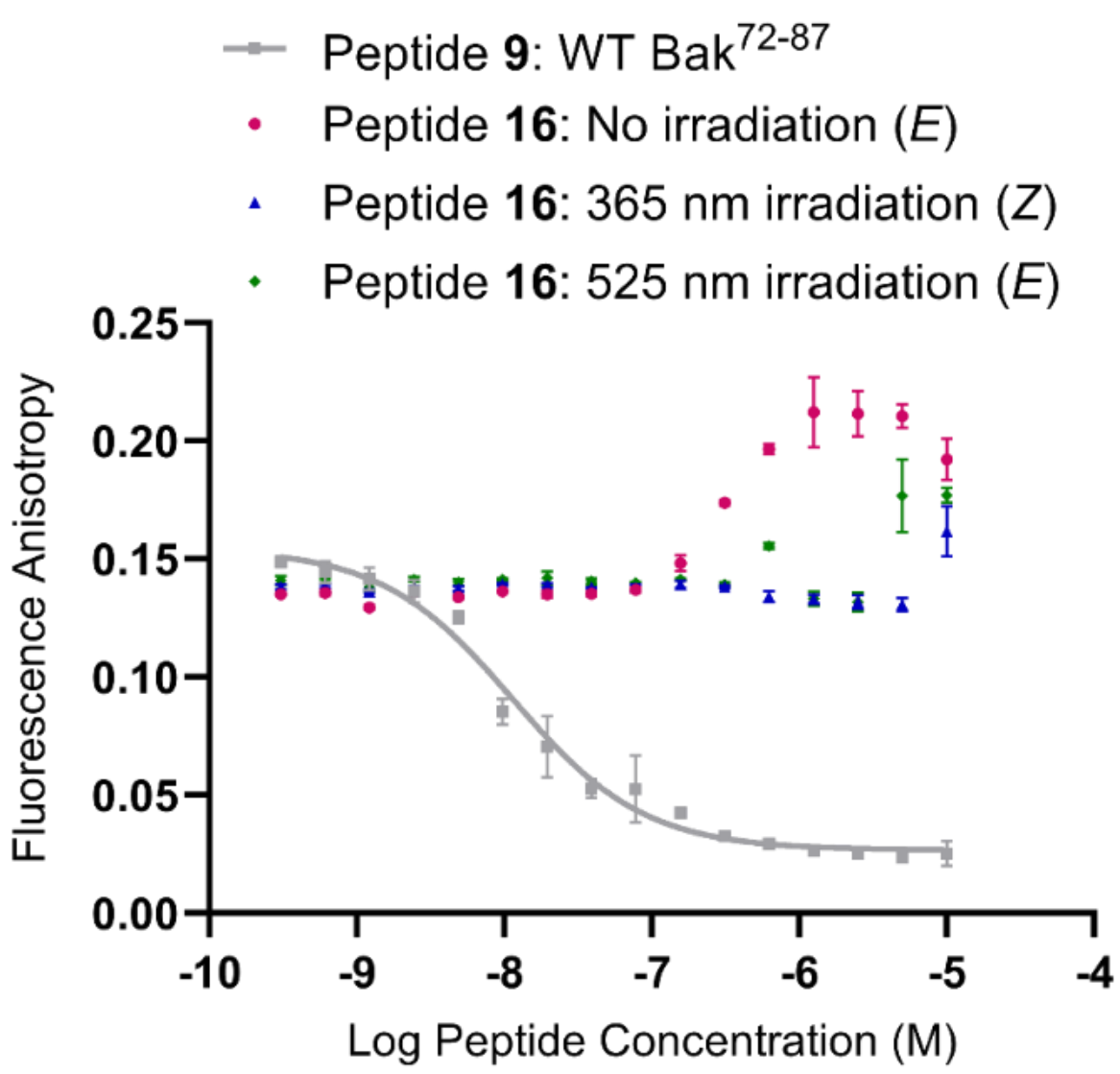

Figure S14: Fluorescence anisotropy competition binding curve for the binding of peptide **16** to Bcl-x$_L$ protein$^{1-209}$(Δ45 84). The plate was measured before irradiation, after 365 nm then 525 nm plate irradiation.

Table S1: Binding affinities (IC_{50}) of peptides to Bcl-x_L (Δ45-84) (peptide **9-14, 16**) or MDM2 (peptide **17**, **19** and **20**) as determined by fluorescence anisotropy competition binding experiments. N.B = No binding observed; P.B or * = partial binding observed.

Compound	IC_{50} (nM)		pIC_{50} ± SEM
**9** (Wildtype Bak^{72-87})	247 ± 78		6.61 ± 0.03
**10** ([A^{73},F^{81},$C^{77,84}$]-Bak^{72-87})	592 ± 150*		6.25 ± 0.05
**11** ([A^{73},F^{81},$C^{77,84}$]-Bak^{72-87}(*i+7*) **AzB**)	**No irradiation**	689 ± 226	6.21 ± 0.04
	**365 nm irradiation**	44 ± 12	7.4 ± 0.05
	**13 h post-365 nm irradiation**	800 ± 724	6.17 ± 0.12
**13** ([A^{73},F^{81},$C^{77,84}$]-Bak^{72-87}- Arg tag)	263 ± 160 (Cysteines reduced with TCEP)<br>N.B (Not pre-reduced)		6.61 ± 0.09
**14** ([A^{73},F^{81},$C^{77,84}$]-Bak^{72-87}- Arg tag - **AzBisPy**)	**No irradiation**	N.B	N.B
	**365 nm plate irradiation**	232 ± 84	6.62 ± 0.05
	**525 nm plate back-irradiation**	N.B	N.B
	**24 h post-365 nm plate irradiation**	228 ± 95	6.64 ± 0.06
	**48 h post-365 nm irradiation**	155 ± 23	6.80 ± 0.08
	**Sample irradiation – 365 nm PSS**	252 ± 38	6.61 ± 0.02
	**Sample irradiation – 525 nm PSS**	N.B	N.B
**16** [$A^{73-74,78,81}$,$C^{77,84}$]-Bak^{72-87}- Arg tag - **AzBisPy**	**No irradiation**	N.B	N.B
	**365 nm plate irradiation**	N.B	N.B
	**525 nm plate back-irradiation**	N.B	N.B
**17** (Wildtype $p53^{17-28}$)	1180 ± 40*		5.93 ± 0.12
**19** ([$C^{21,28}$,S^{27}]-$p53^{17-28}$)	P.B		P.B
**20** ([$C^{21,28}$,S^{27}]-$p53^{17-28}$-**AzBisPy**)	**Sample irradiation – 365 nm PSS**	234 ± 42	6.61 ± 0.07
	**Sample irradiation – 525 nm PSS**	P.B	P.B

Experimental Procedures

Materials and general methods

Reagents and solvents were purchased from commercial suppliers and used as supplied unless otherwise stated. 9-fluorenylmethoxycarbonyl (Fmoc)-protected amino acids were purchased from CEM (Buckingham, United Kingdom) and Fluorochem (Hadfield, United Kingdom). TentaGel™ S-RAM resin, piperidine, acetic anhydride, 2,2'-(ethylenedioxy)diethanethiol (DODT), triisopropylsilane (TIPS), 5(6)-carboxyfluorescein (FAM), acetonitrile (MeCN) and formic acid were purchased from Sigma-Aldrich (Gillingham, United Kingdom). *N,N'*-Diisopropylcarbodiimide (DIC), Ethyl 2-cyano-2-(hydroxyimino)acetate (Oxyma Pure) and *N*,*N*-Diisopropylethylamine (DIPEA) were from Fluorochem. Diethyl ether was purchased from VWR (Lutterworth, United Kingdom). Dimethylformamide (DMF), dichloromethane (DCM) and trifluoroacetic acid (TFA) were obtained from Fisher Scientific UK (Loughborough, United Kingdom).

Reactions were monitored using thin-layer chromatography (TLC) on Merck silica gel 60 F254 plates and visualized with UV light (254 nm) and/or an appropriate TLC stain. Flash column chromatography was performed using silica gel (Fluorochem, 60 Å). Nuclear Magnetic Resonance (NMR) spectra were recorded using Bruker *Avance* III 400 MHz spectrometer. Spectra were analysed using MestReNova x64 and calibrated according to the $CDCl_3$, DMSO-d_6 or D_2O residual solvent peaks. Chemical shifts (σ) are reported in parts per million (ppm) and coupling constants (*J*) are reported in Hertz (Hz). High-resolution mass spectrometry (HRMS) was performed by the Imperial Mass Spectrometry service, using an electrospray ionization (ESI) mode. Liquid chromatography-mass spectrometry (LC-MS) was measured on an Agilent 1260 Infinity II LC-MS using a Poroshell 120 EC-C18 column (2.1 x 50 mm, 1.9 µm) or Aeris™

PEPTIDE XB-C18 column (100 x 2.1 mm, 3.6 µm). A gradient of 5-95% MeCN and water, both containing 0.1% formic acid over 7.5 min was used. Or a Waters LC-MS was used with an analytical reversed-phase Waters XBridge C18 column (100 mm × 4.6 mm, 5 µm, 100 Å, 1.2 ml/min) and a linear gradient of 20% to 98% MeCN in H_2O (0.1% formic acid) over 10 min.

Circular dichroism (CD) spectroscopy

Circular dichroism was measured using a Chirascan V100 CD spectrometer (Applied photophysics) in the 190-400 nm range. Measurements were performed at 25 °C with a 1 nm band width, 1 nm step size and 0.5 s per step. Peptides were dissolved in 25% or 50% acetonitrile in water at 25-30 µM (300 µL) (10% 2,2,2-trifluoroethanol (TFE) added in the measurements indicated) and measured in a 1 mm path length QS Quartz cuvette. The photoswitchable stapled peptides were measured before irradiation, after 365 nm irradiation (3 minutes) and subsequent 525 nm irradiation (5 minutes). Three replicates for each measurement were averaged and smoothed using Savitsky-Golay smoothing (window size 5). The background spectra of 25-50% acetonitrile in water was subtracted from the averaged peptide spectra. The measured CD values in mdeg and were converted to mean residue ellipticity [θ] ($deg.cm^2.dmol^{-1}$). The mean residue weight was calculated based on the molecular weight of the stapled peptide, where N is the number of amino acid residues (including *N*-terminal acylation).

$$[\theta] = \frac{mdeg \ \times mean\ residue\ weight\ in\ g/mol}{path\ length\ in\ mm\ \times concentration\ in\ mg/mL}$$

$$Mean\ residue\ weight\ (MRW) = \frac{Molecular\ weight}{N-1}$$

UV-Vis spectroscopy

PSS compositions were determined via UV-Vis spectroscopy using an Agilent Cary 60 spectrophotometer and a quartz cuvette (10 mm path length). To calculate PSS, irradiation with Sahlmann Photochemical Solutions LEDs were used with wavelengths of 340 nm (3 x 55 mW Seoul CUD4AF1B), 365 nm (3 x 1050 mW Nichia NCSU276CT), 495 nm (3 x 750 mW Nichia NCSE219B-V1) and 525 nm (3 x 450 mW Nichia NCSG219B-V1). In order to determine *Z-E* thermal isomerisation kinetics by UV-Vis spectroscopy, the relevant compound was dissolved in the necessary solvent, placed into a quartz cuvette and irradiated to the 365 nm PSS. Absorption spectra were collected over time whilst the sample was held at a fixed temperature. Plots of the absorbance at a fixed wavelength vs. time were thus obtained and fitted using a mono-exponential function utilising a Levenberg-Marquardt iteration algorithm. The thermal isomerisation kinetics were determined at a series of temperatures for a given compound to obtain an Eyring plot. From this plot, the thermal isomerisation kinetics at the desired temperature could be extrapolated. For photostability measurements, the photoswitch was switched to *Z* by irradiating with 365 nm and back to *E* by irradiating with 525 nm and this was repeated 10 times. Concentration dependence measurements to determine the molar extinction coefficient were repeated three times and measured at 10 µM, 15µM, 20 µM, 25 µM and 30 µM.

Fluorescence anisotropy

For saturation binding assays, Bcl-x_L^{1-209}(Δ45-84) protein (2 µM, 7.5 µL) was pipetted into a black 384-well plate and serially diluted (2-fold) in assay buffer (50 mM TRIS (pH 8), 150 mM NaCl, 5% glycerol) across four rows. FAM-labelled Bak^{72-87} peptide **FAM-9** (2 nM, 7.5 µL) was added to the first three rows, while buffer was added in the fourth row to

measure the background fluorescence from the protein. The final well volumes were 15 µL with concentrations of Bcl-x_L^{1-209}(Δ45-84) from 1000 nM to 0.02 nM and peptide **FAM-9** at 1 nM.

For competition binding experiments, a buffer containing 50 mM TRIS, 150 mM NaCl, 5% glycerol, 10% DMSO at pH 8 was used with a 10% DMSO concentration maintained consistently across the plate. The non-fluorescent Bak or p53 peptides (90 µM or 30 µM, 5 µL) were pipetted into a black 384-well plate and serially diluted (twofold) in assay buffer across four rows each. Bcl-x_L^{1-209}(Δ45-84) (300 nM, 5 µL) or MDM2 (450 nM) were added to all wells and fluorescently-labelled peptide (**FAM-9** (3 nM, 5 µL) for Bcl-x_L or **Flu-18** (75 nM, 5 µL) for MDM2) were added to the first three rows, while buffer was added in the fourth row. Final well volumes of 15 µL with concentrations of non-fluorescent peptides ranging from 30-10 µM to 3-0.31 nM, Bcl-x_L^{1-209}(Δ45-84) at 100 nM and FAM-labelled Bak^{72-87} peptide at 1 nM <u>or</u> MDM2 at 150 nM and Flu-labelled $p53^{15-32}$ at 25 nM. Plates were incubated in darkness for 20 min at room temperature on an orbital shaker before the fluorescent polarisation measurement. For the azobenzene-stapled peptide, fluorescence polarisation was measured using a Perkin Elmer Envision 2104 Multilabel Reader (excitation filter: FITC FP 480 nm, Emission filters: FITC FP P-pol 535 nm and FITC FP S-pol 535 nm). Fluorescence polarisation of the azobispyrazole-stapled peptides were measured using a CLARIOstar^{PLUS} plate reader (BMG LABTECH) with an excitation wavelength of 482 (16 nm bandwidth), emission wavelength of 530 nm (40 nm bandwidth) and a longpass dichroic beam split of 504 nm.

For the azobispyrazole-stapled peptides, plate and sample irradiation methods were used. For the plate irradiation method, the non-irradiated photoswitchable peptide was added to the 384-well plate and fluorescence polarisation was measured after a 20 min incubation period in the dark on the orbital shaker. The plate was then irradiated with 365 nm for 10 minutes. The LED was moved periodically across the wells to ensure uniform irradiation, and the fluorescence polarisation was measured after another 20 min incubation period. The plate was then irradiated with 525 nm for 10 min and the fluorescence polarisation measured after a final 20 min incubation period. For the sample irradiation method, two samples of photoswitchable stapled peptide were irradiated to 365 nm PSS (3 min irradiation) and 525 nm PSS (365 nm irradiation for 3 min then 525 nm irradiation for 3-5 min) prior to addition to the plate.

Rows without FAM-labelled peptide were used to correct background fluorescence in parallel and perpendicular detector planes due to protein and unlabelled peptide. Anisotropy values were calculated using the following equation

$$A = (I_{\parallel} - GI_{\perp})/(I_{\parallel} + 2GI_{\perp})$$

where $I_{\parallel}$ and $I_{\perp}$ are the fluorescence intensities in the parallel and perpendicular planes respectively, and G is the instrument correction factor.

$$G = I_{\perp}/I_{\parallel}$$

For the saturation binding experiments, the anisotropy values were plotted against log Bcl-x_L concentration, fitted with a four-parameter fit using GraphPad Prism, obtained K_d values for each experiment were averaged and the standard error of the mean calculated. For the competition binding experiments, anisotropy values for each compound were plotted against log peptide concentration, fitted with a one-site binding $logIC_{50}$ fit using GraphPad Prism and normalized to the *Z*-form peptide **11**, **14** or **20.**

Peptide Synthesis

Solid-phase peptide synthesis (SPPS)

Peptides were synthesised by solid phase peptide synthesis (SPPS) using the Liberty Blue automated microwave peptide synthesiser (CEM) and a standard 9-fluorenylmethoxycarbonyl/*tert*-Butyl (Fmoc/*t*Bu) strategy. Tentagel™ S-RAM (Fmoc protected, rink amide) resin (50 µmol scale, 0.51 mmol/g) was used to obtain peptides as C-terminal amides. Couplings were performed using the Fmoc-protected amino acid (0.2 M), N,N'-diisopropylcarbodiimide (DIC) (0.5 M), and Oxyma Pure (0.5 M) in DMF at 90 °C for 2 min (single coupling) or 2 x 2 min (double coupling). Cleavage of the N-terminal Fmoc protecting group was accomplished by using 10 % (v/v) piperidine in DMF for 1 min (single couplings) or 1.5 min (double couplings) at 90°C. Once synthesised, the resin was washed with DMF (5 x 3 mL) and DCM (5 x 3 mL).

Peptide N-acetylation and cleavage

Following completion of automated SPPS, peptides were manually acetylated at the N-terminus with 5% acetic anhydride and 5% DIPEA in DMF (3 mL) for 3 x 10 min. For peptide **FAM-9**, 3 eq. 5(6)-Carboxyfluorescein (FAM, 150 µmol) were manually coupled to the N-terminus with 5 eq. DIC (250 µmol) and 5 eq. Oxyma Pure in DMF overnight at room temperature. Cleavage from the resin and simultaneous side chain deprotection was accomplished through shaking with a mixture of TFA/TIPS/H_2O/DODT (92.5/2.5/2.5/2.5, 3 mL) for 2.5 h. The peptide was precipitated with ice cold diethyl ether and left in the freezer for 30 minutes, centrifuged (4400 rpm, 2 min) and the supernatant was discarded. The peptide was washed with ice-cold diethyl ether (3 x 20 mL) and centrifuged after each wash. The pellet was dissolved in a mixture of MeCN/H_2O and lyophilized.

Peptide purification

The purification of the peptides and stapled peptides was achieved on a Shimadzu LC-20AR preparative HPLC system. In the Shimadzu HPLC system, a preparative reversed-phase Phenomenex Aeris Peptide XB-C18 column (150 mm x 21 mm, 5 µm, 100 Å) with a flow rate of 20 mL/min was used with different linear gradients of eluent B (0.08 % (v/v) TFA in MeCN) in eluent A (0.1 % (v/v) TFA in water). Purity of the peptides were determined on a Shimadzu LC-2030C 3D analytical HPLC system using an analytical reversed-phase Phenomenex Aeris Peptide XB-C18 column (150 mm x 4.6 mm, 3.6 µM, 100 Å) with a flow rate of 1.5 mL/min, different linear gradients of eluent B in eluent A over 7.5-15 min and detection at λ = 220 nm and/or 365 nm. The identity of the pure peptides was additionally confirmed using an Agilent 6546 Bio LC/Q-TOF (Imperial College London Agilent Measurement Suite). The observed masses agreed with the calculated masses and a high purity could be obtained for all compounds.

Peptide stapling

Reactivity test of azobispyrazole with 2-phenylethanethiol

8 (5.0 mg, 1.0 eq.) was dissolved in DMF-*d7* (0.7 mL) and a ^{1}H NMR spectrum was recorded. 2-phenylethane-1-thiol (2.0 eq.) then DIPEA (2.0 eq.) was added and a ^{1}H NMR spectra was recorded after each addition and until the reaction reached completion.

Peptide stapling with an azobenzene photoswitchable cross-linker

For the synthesis of peptide **11**, Bak$^{72\text{-}87}$ *i, i+7* peptide **10** was used. Peptide **10** (5.0 mg, 2.9 µmol, 1.0 eq.) was dissolved in DMF (5.0 mL). DODT (1.0 uL, 6.0 µmol, 2.0 eq.) was added and the peptide was shaken for 1 hour at room

temperature. Stapling was initiated by the addition of a 2 mM **AzB** cross-linker solution in 50 mM Tris buffer (pH 8.3) in 3 x 1.5 mL portions at 30 min intervals (overall 3.0 eq., 9.0 µmol of cross-linker). The reaction was shaken overnight at room temperature with an overall reaction time of 16 hours. The reaction mixture was concentrated to dryness *in vacuo* and the stapled peptide **11** was isolated by preparative HPLC. The crude product was dissolved in 25% MeCN in H_2O with 0.1% TFA (4.0 mL) and was purified by preparative HPLC using a linear gradient of 25-55% eluent B over 20 minutes. The solid was redissolved in 20% MeCN in H_2O with 0.1% TFA (4.0 mL) and a further purification was carried out using a 15-45% gradient of eluent B over 20 minutes to obtain pure peptide **11**.

Peptide stapling with an azobispyrazole photoswitchable cross-linker

For the synthesis of peptide **12**, Bak$^{72\text{-}87}$ *i, i+7* **10** (5.0 mg, 2.9 µmol, 1.0 eq.) and $NaBH_4$ (0.5 mg, 11.7 µmol, 4.0 eq.) were dissolved in anhydrous DMF (5.0 mL) and stirred for 1 h at room temperature under nitrogen. DIPEA (5.0 µL, 29.3 µmol, 10.0 eq.) was added in DMF (0.5 mL) and stirred for 10 min. AzBisPy **8** (4.9 mg, 8.8 µmol, 3.0 eq.) was dissolved in anhydrous DMF (4.0 mL) under nitrogen and added in three portions to the peptide solution every 20 min at room temperature. The reaction was stirred overnight at room temperature for 15 h and LCMS showed stapled peptide present. The product was precipitated with ice-cold diethyl ether (120 mL) then centrifuged (4000 rpm, 4 min). The pellet was dissolved in MeCN/H_2O and lyophilised. The crude product was dissolved in 30% MeCN in H_2O with 0.1% TFA (3.0 mL) and was purified by preparative HPLC with a linear gradient of 30-50% MeCN over 20 minutes to obtain pure peptide **12**.

For the synthesis of peptide **14**, Bak$^{72\text{-}87}$ *i, i+7* **13** (20.4 mg, 8.0 µmol, 1.0 eq.) and $NaBH_4$ (1.2 mg, 31.9 µmol, 4.0 eq.) were dissolved in anhydrous DMF (20.4 mL) and stirred for 1 h at room temperature under nitrogen. DIPEA (13.9 µL, 80.0 µmol, 10.0 eq.) was added in DMF (2.1 mL) and stirred for 10 min. AzBisPy **8** (13.3 mg, 23.9 µmol, 3.0 eq.) was dissolved in anhydrous DMF (16.4 mL) under nitrogen and added in three portions to the peptide solution every 15 min at room temperature. The reaction was stirred overnight at room temperature for 20 h and LCMS showed stapled peptide present. The reaction solvent was removed under a flow of nitrogen and the remaining solid was dissolved in MeCN/H_2O and lyophilised. The crude product was dissolved in 25% MeCN in H_2O with 0.1% TFA (15.4 mL) and was purified by preparative HPLC with a linear gradient of 25-55% MeCN over 20 minutes to obtain peptide **14** (2.75 mg, 0.93 µmol, 12%).

For the synthesis of peptide **16**, Bak$^{72\text{-}87}$ *i, i+7* **15** (5.0 mg, 1.0 eq.) and $NaBH_4$ (4.0 eq.) were dissolved in anhydrous DMF (5.0 mL) and stirred for 1 h at room temperature under nitrogen. DIPEA (10.0 eq.) in DMF (0.5 mL) was added and stirred for 10 min. AzBisPy **8** (3.0 eq.) was dissolved in anhydrous DMF (4.0 mL) under nitrogen and added in three portions to the peptide solution every 30 min at room temperature. The reaction was stirred overnight at room temperature for 22 h. The product was precipitated with ice-cold diethyl ether (120 mL) and centrifuged (4000 rpm, 3 min). The pellet was dissolved in MeCN/H_2O and lyophilised. The crude product was dissolved in 25% MeCN in H_2O with 0.1% TFA (3 mL) and was purified by preparative HPLC with a linear gradient of 25-55% MeCN over 20 minutes to obtain pure peptide **16**.

For the synthesis of peptide **20**, p53[17-28] peptide **19** (5.0 mg, 1.0 eq.) and $NaBH_4$ (4.0 eq.) were dissolved in anhydrous DMF (5.0 mL) and stirred for 1 h at room temperature under nitrogen. DIPEA (10.0 eq.) was added in DMF (0.5 mL) and stirred for 10 min. AzBisPy **8** (3.0 eq.) was dissolved in anhydrous DMF (4.0 mL) under nitrogen and added in three portions to the peptide solution every 20 min at room temperature. The reaction was stirred overnight at room temperature. The product was precipitated with ice-cold diethyl ether (120 mL) and centrifuged (4000 rpm, 3 min). The pellet was dissolved in MeCN/H_2O and lyophilised. The crude product was dissolved in 3% DMSO, 35% MeCN in H_2O with 0.1% TFA (3.0 mL) and was purified by preparative HPLC with a linear gradient of 35-65% MeCN over 20 minutes to obtain pure peptide **20**.

Preparative HPLC traces of peptide purifications:

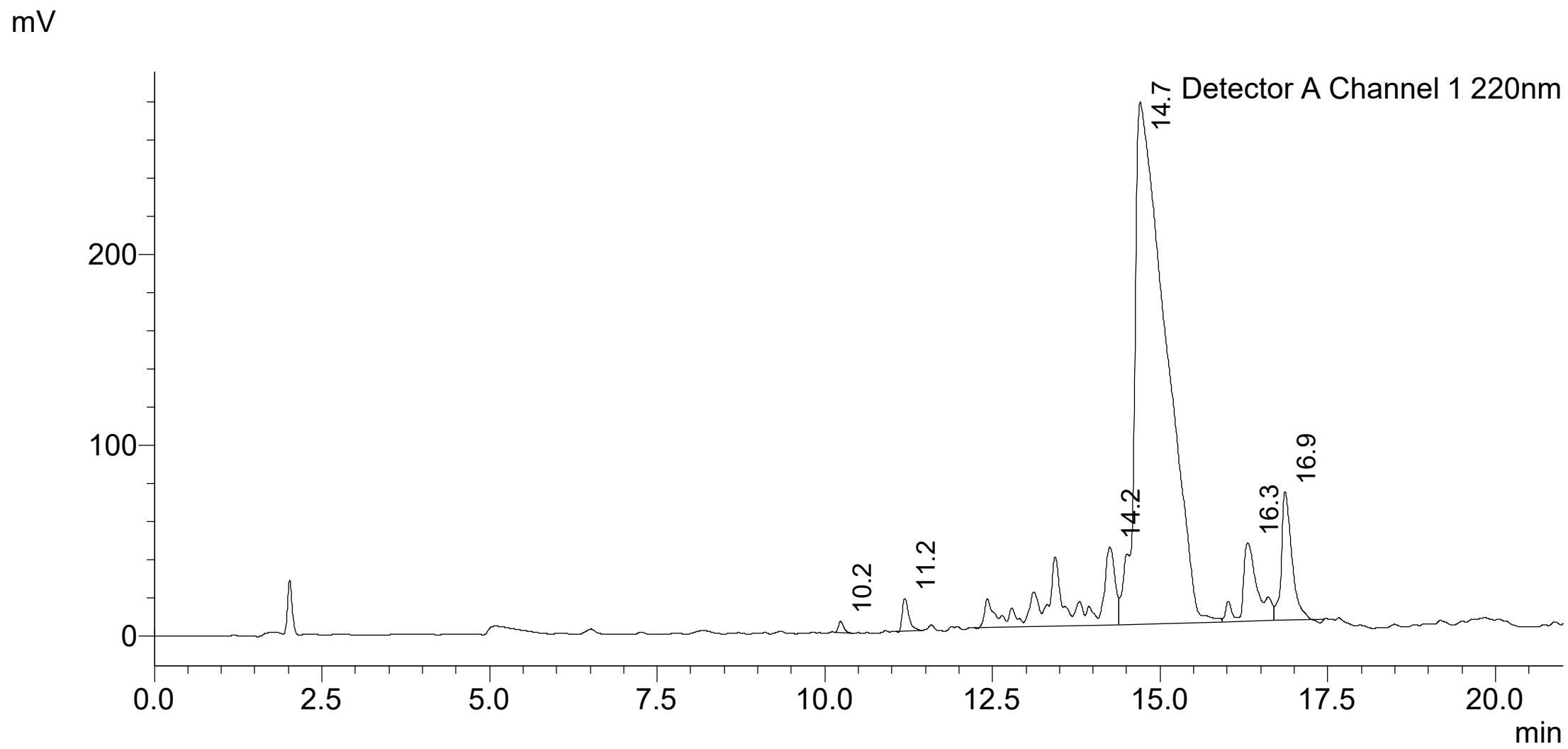

Figure S15: Peptide **9** preparative HPLC purification trace at 220 nm (14.7 min elution; 75% crude purity; 10-40% MeCN over 20 minutes).

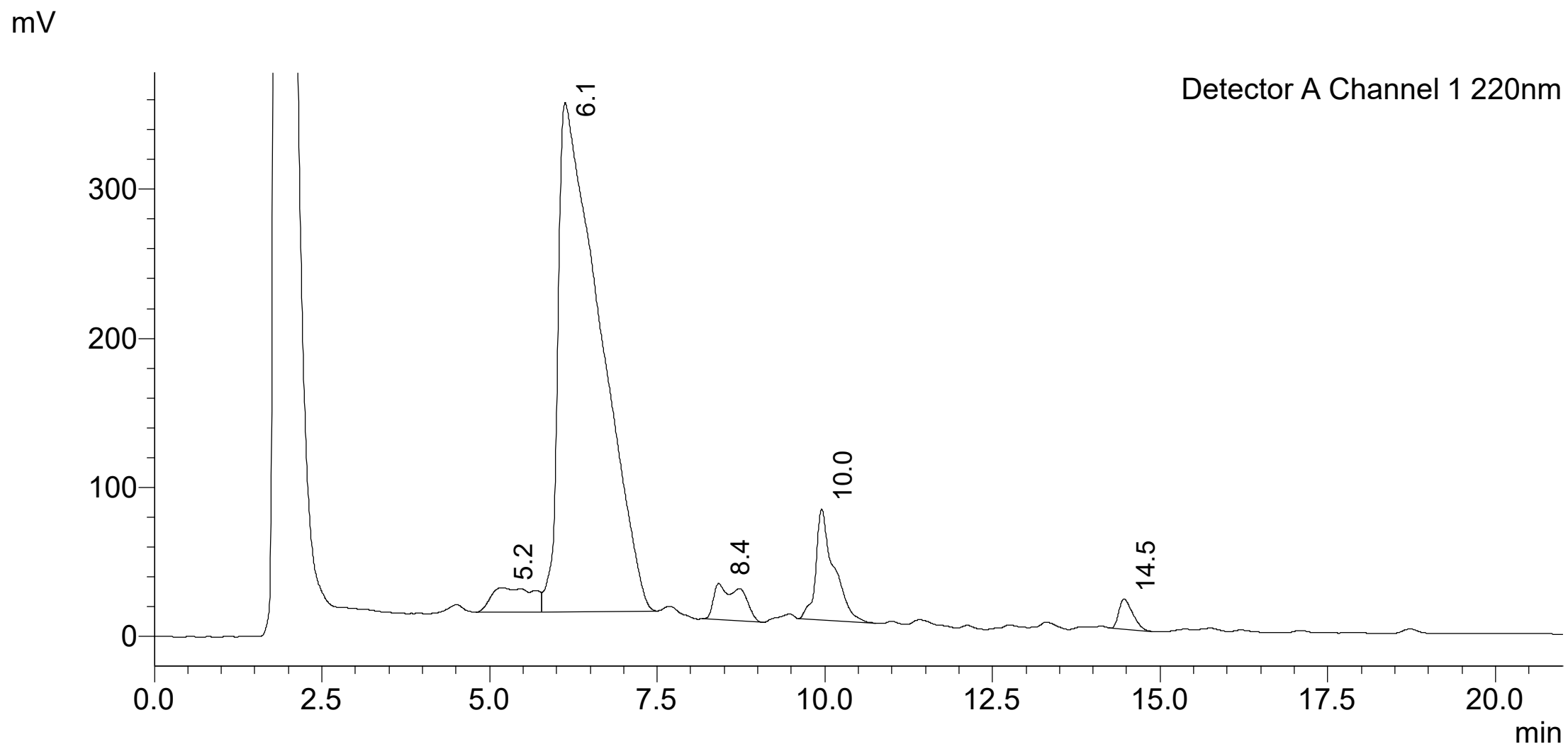

Figure S26: Peptide **10** preparative HPLC purification trace at 220 nm (6.1 min elution; 83% crude purity; 30-60% MeCN over 20 minutes).

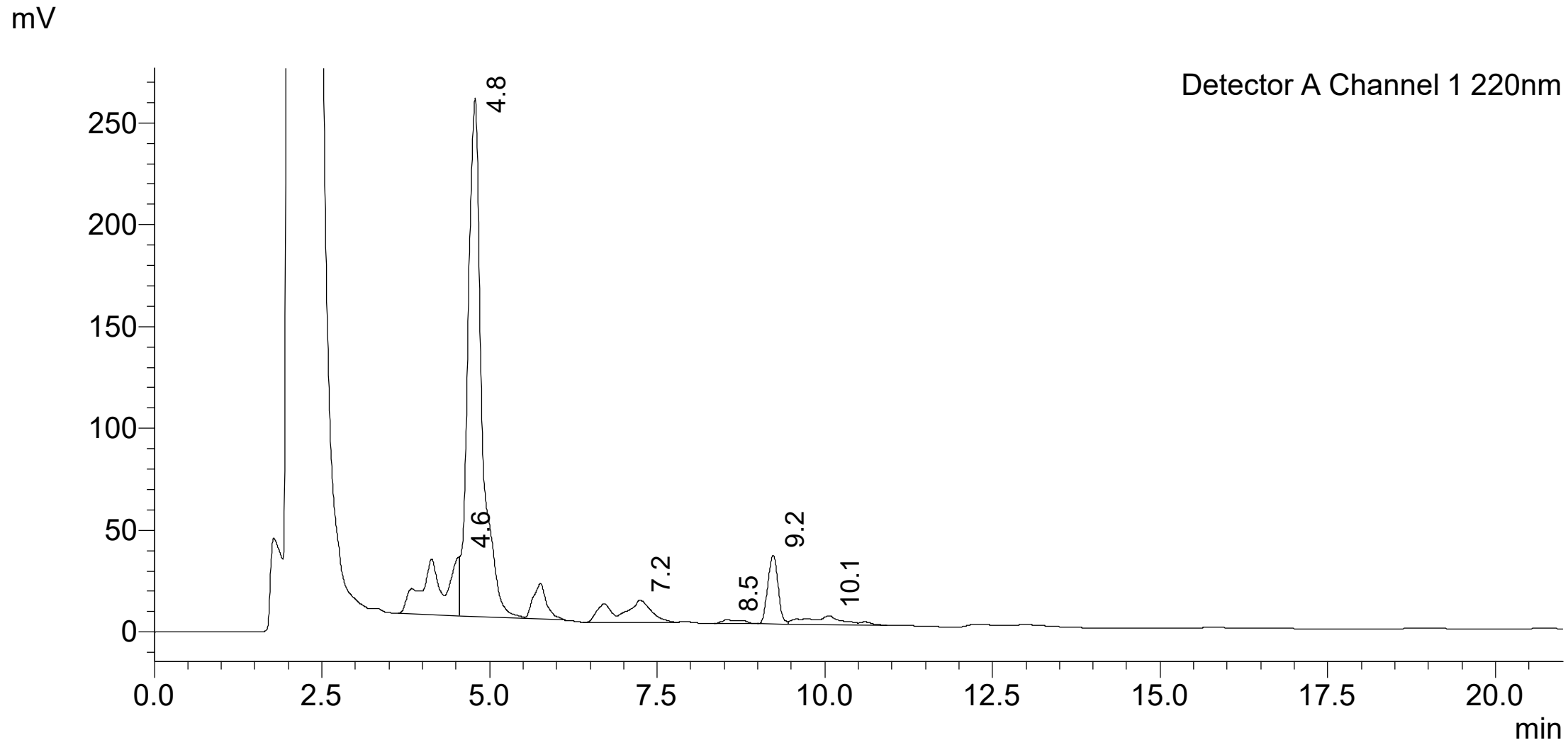

Figure S37: Peptide **11** preparative HPLC purification trace at 220 nm (4.8 min elution; 69% crude purity; 25-55% MeCN over 20 minutes).

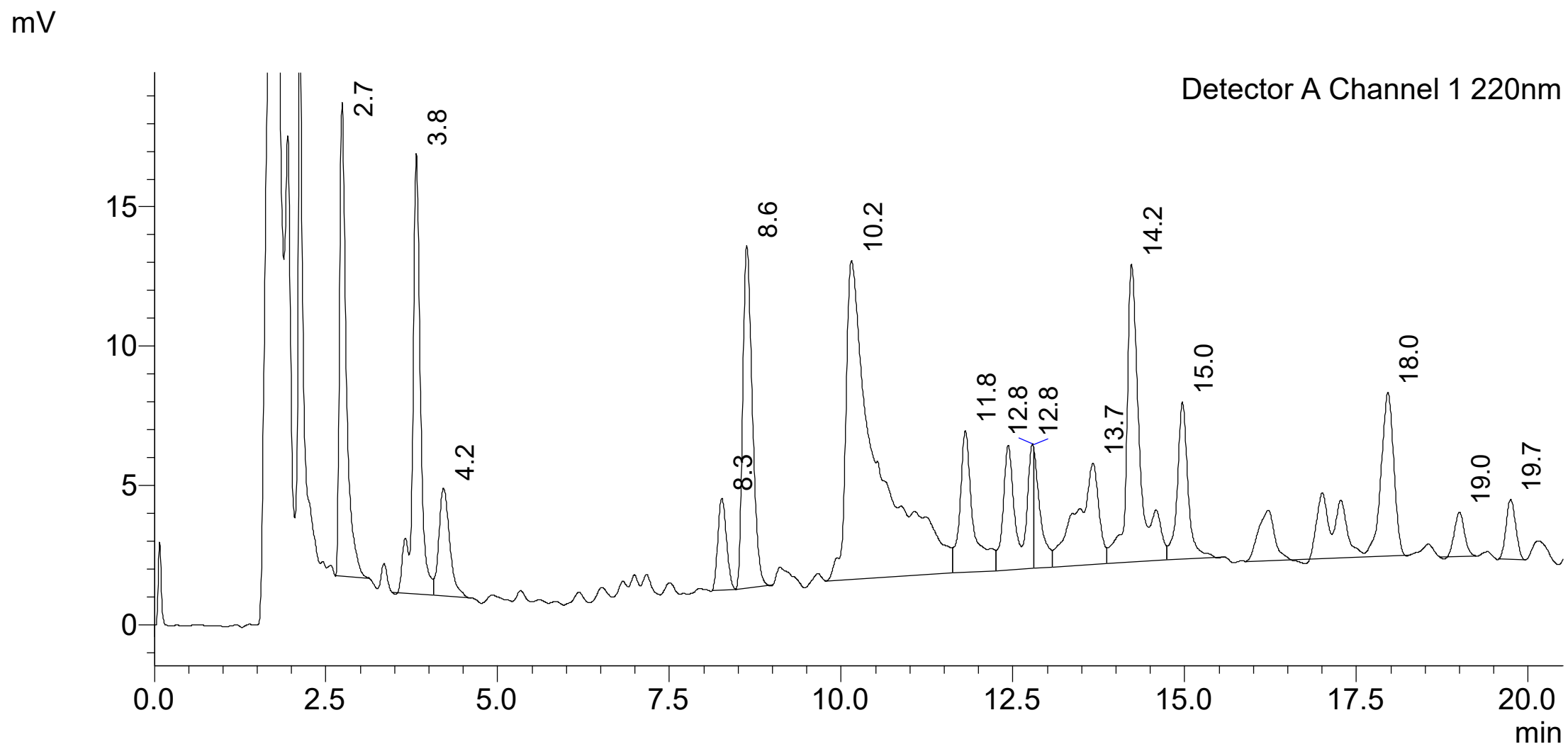

Figure S48: Peptide **12** preparative HPLC purification trace at 220 nm (10.2 min elution; 25% crude purity; 30-50% MeCN over 20 minutes).

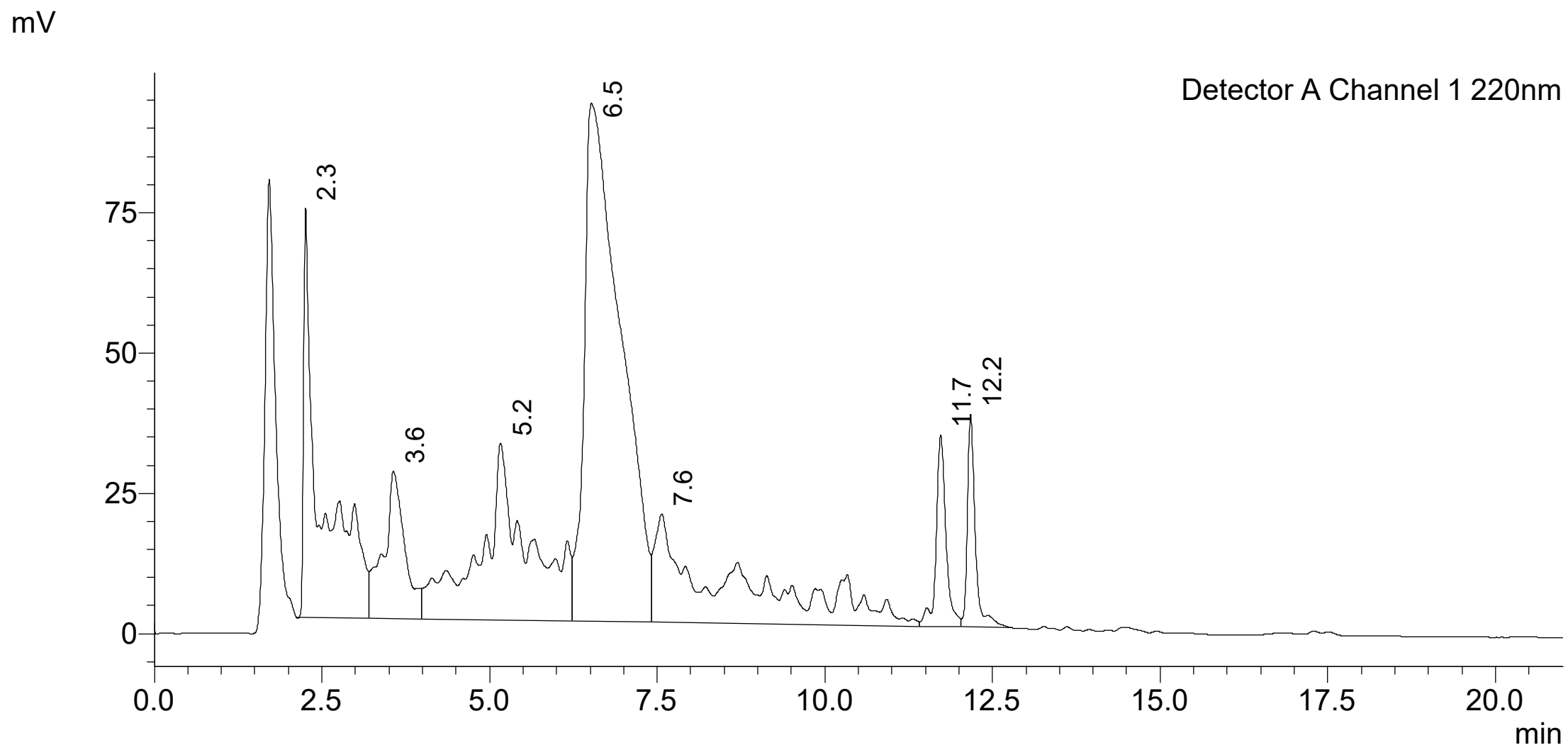

Figure S59: Peptide **13** preparative HPLC purification trace at 220 nm (6.5 min elution; 38% crude purity; 25-55% MeCN over 20 minutes).

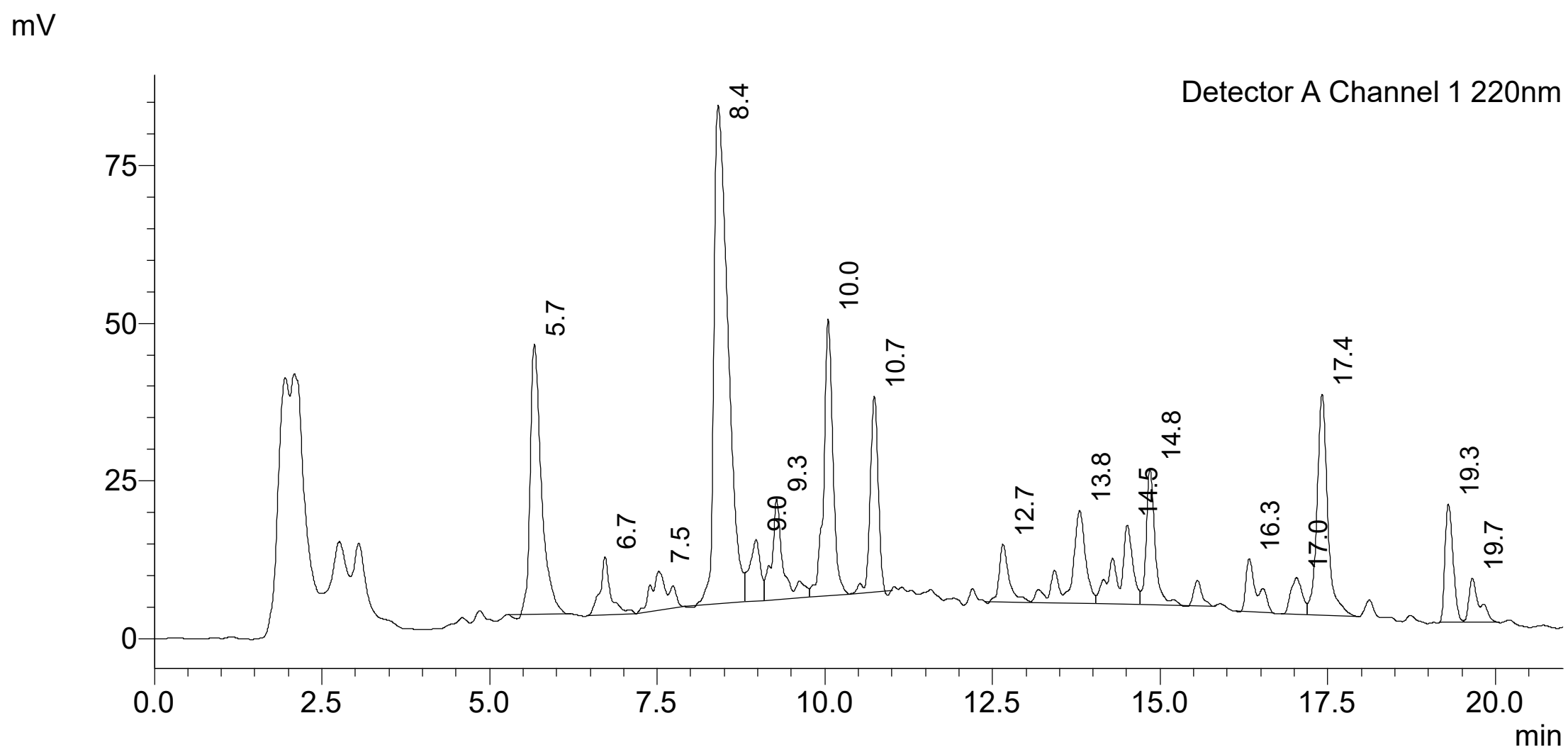

Figure S20: Peptide **14** preparative HPLC purification trace at 220 nm (8.4 min elution; 26% crude purity; 25-55% MeCN over 20 minutes).

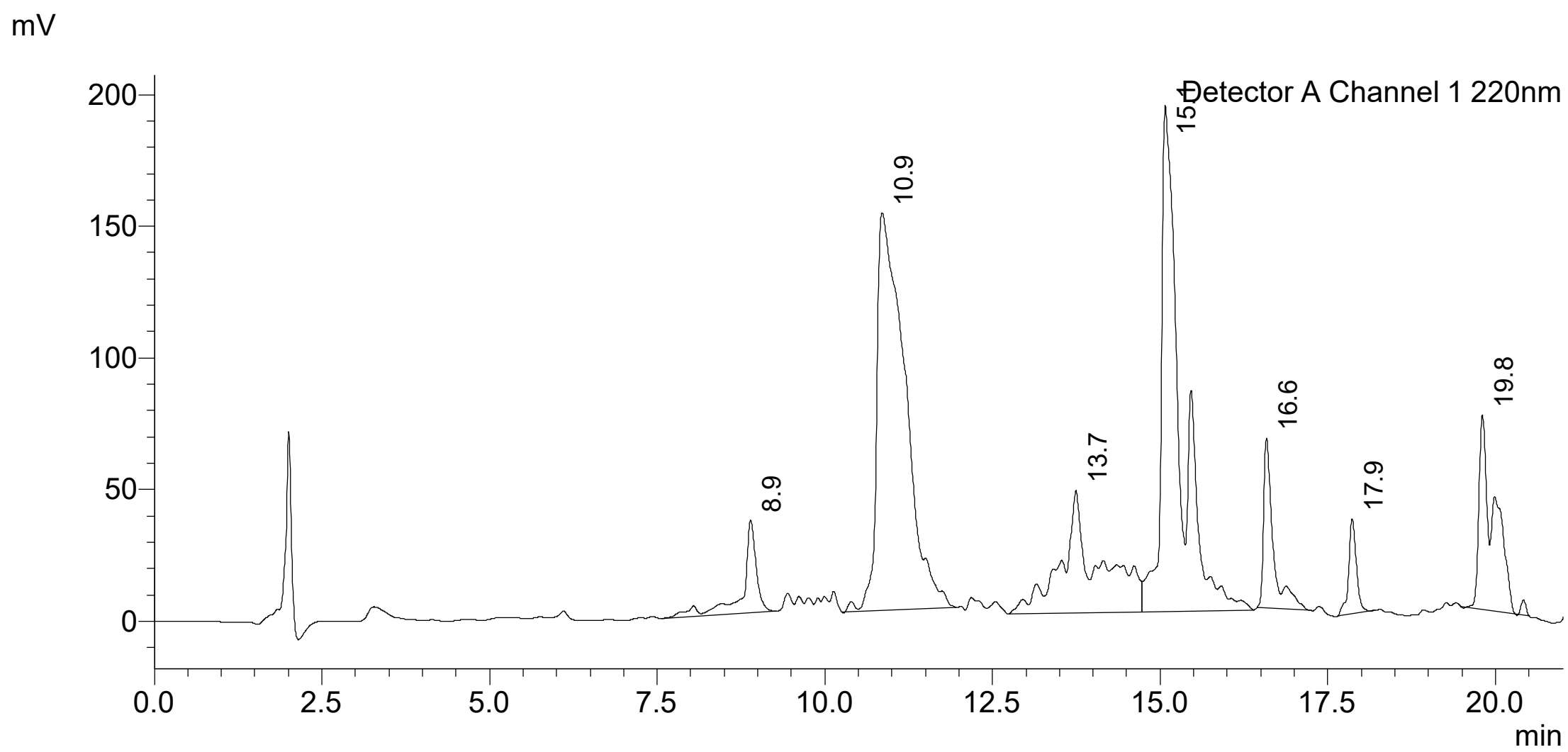

Figure S21: Peptide **15** preparative HPLC purification trace at 220 nm (10.9 min elution; 35% crude purity; 15-55% MeCN over 20 minutes).

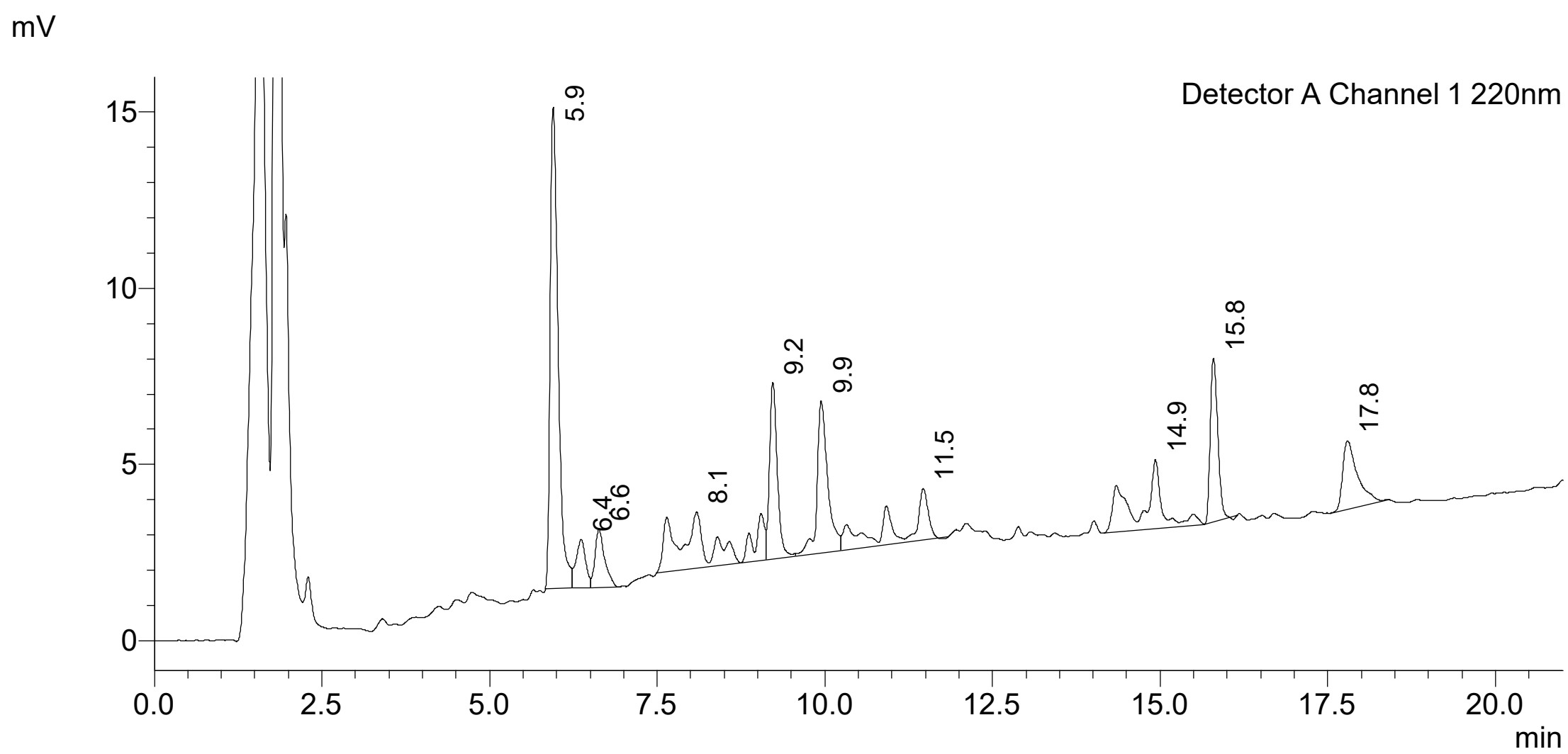

Figure S22: Peptide **16** preparative HPLC purification trace at 220 nm (5.9 min elution; 25% crude purity; 25-55% MeCN over 20 minutes).

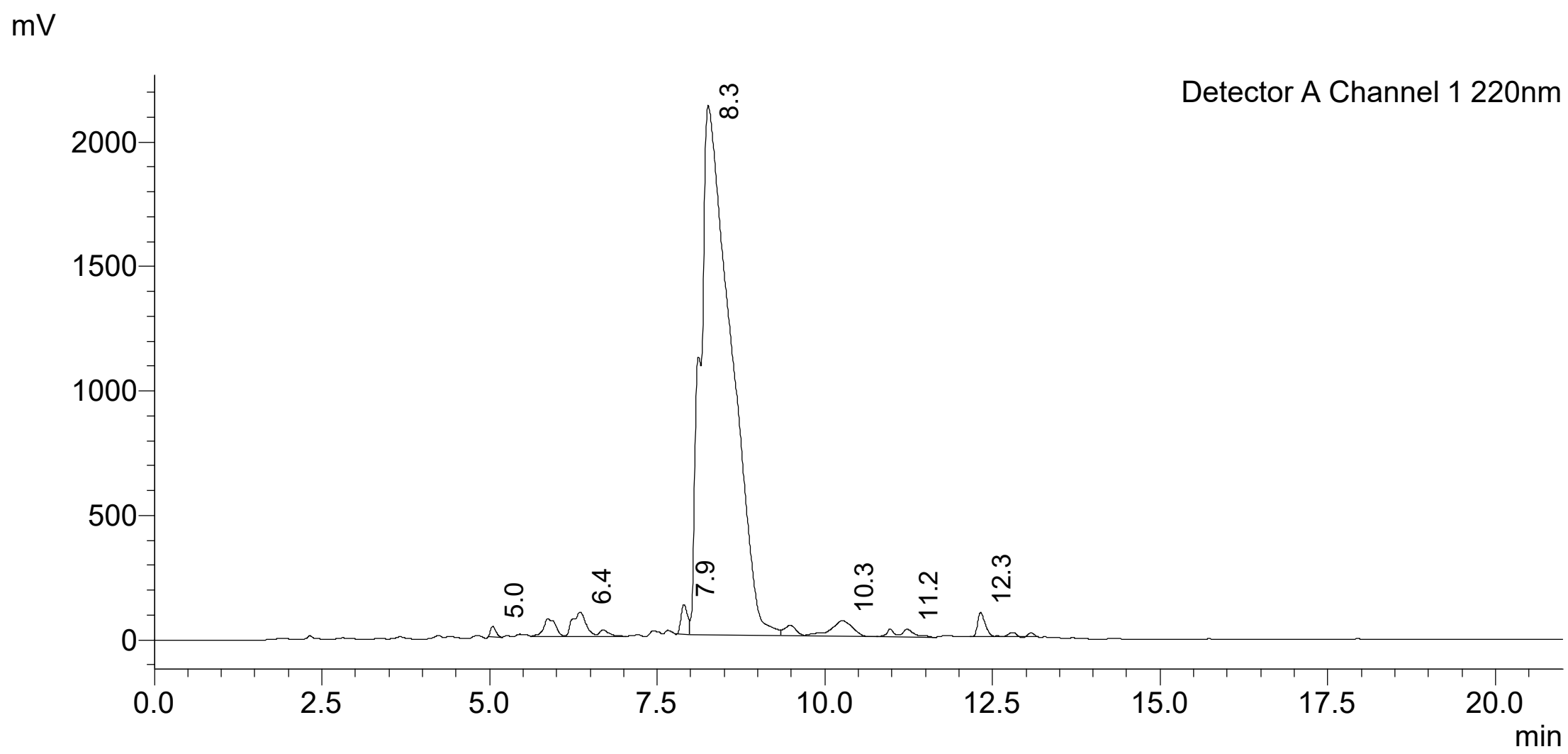

Figure S23: Peptide **17** preparative HPLC purification trace at 220 nm (8.3 min elution; 90% crude purity; 25-55% MeCN over 20 minutes).

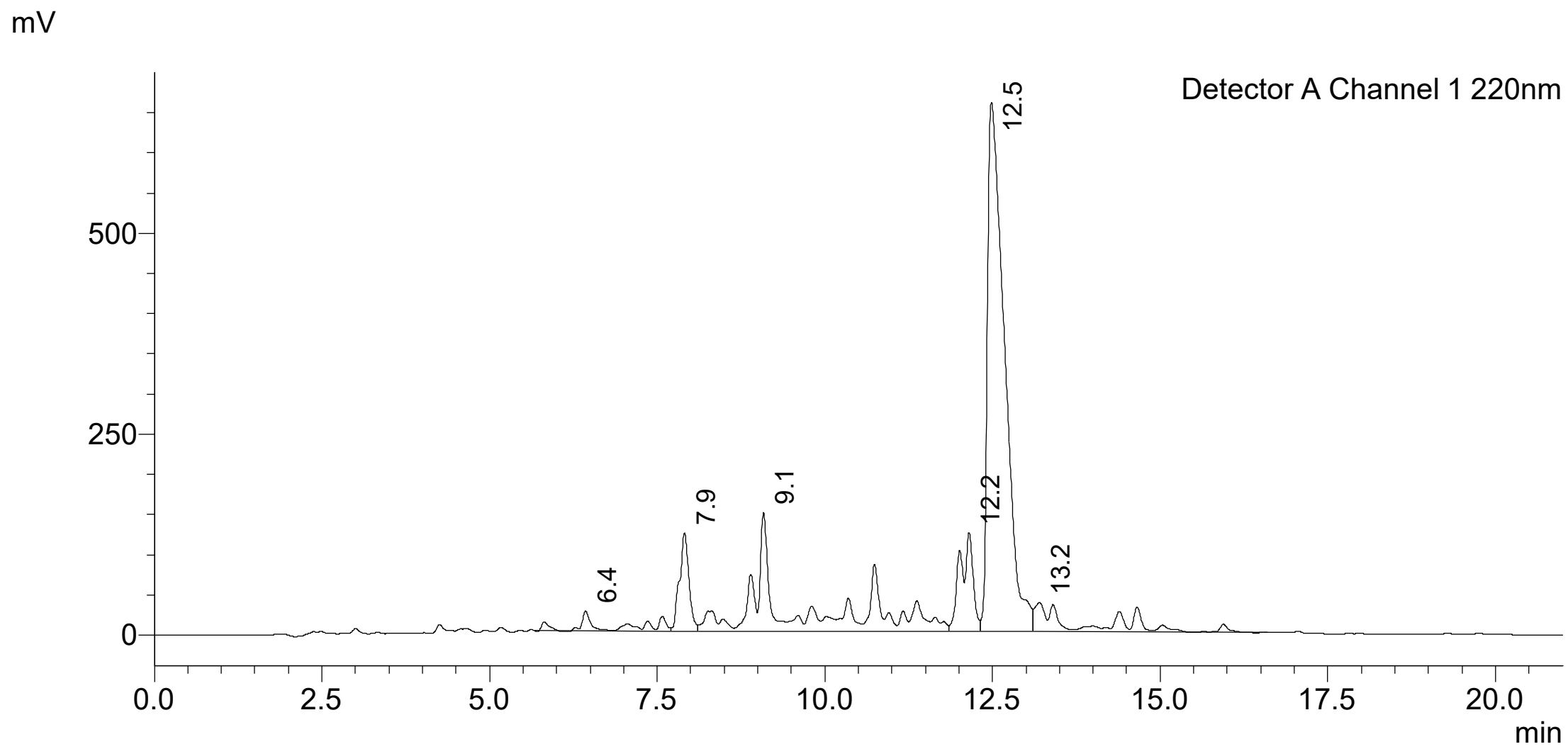

Figure S24: Peptide **19** preparative HPLC purification trace at 220 nm (12.5 min elution; 53% crude purity; 25-55% MeCN over 20 minutes).

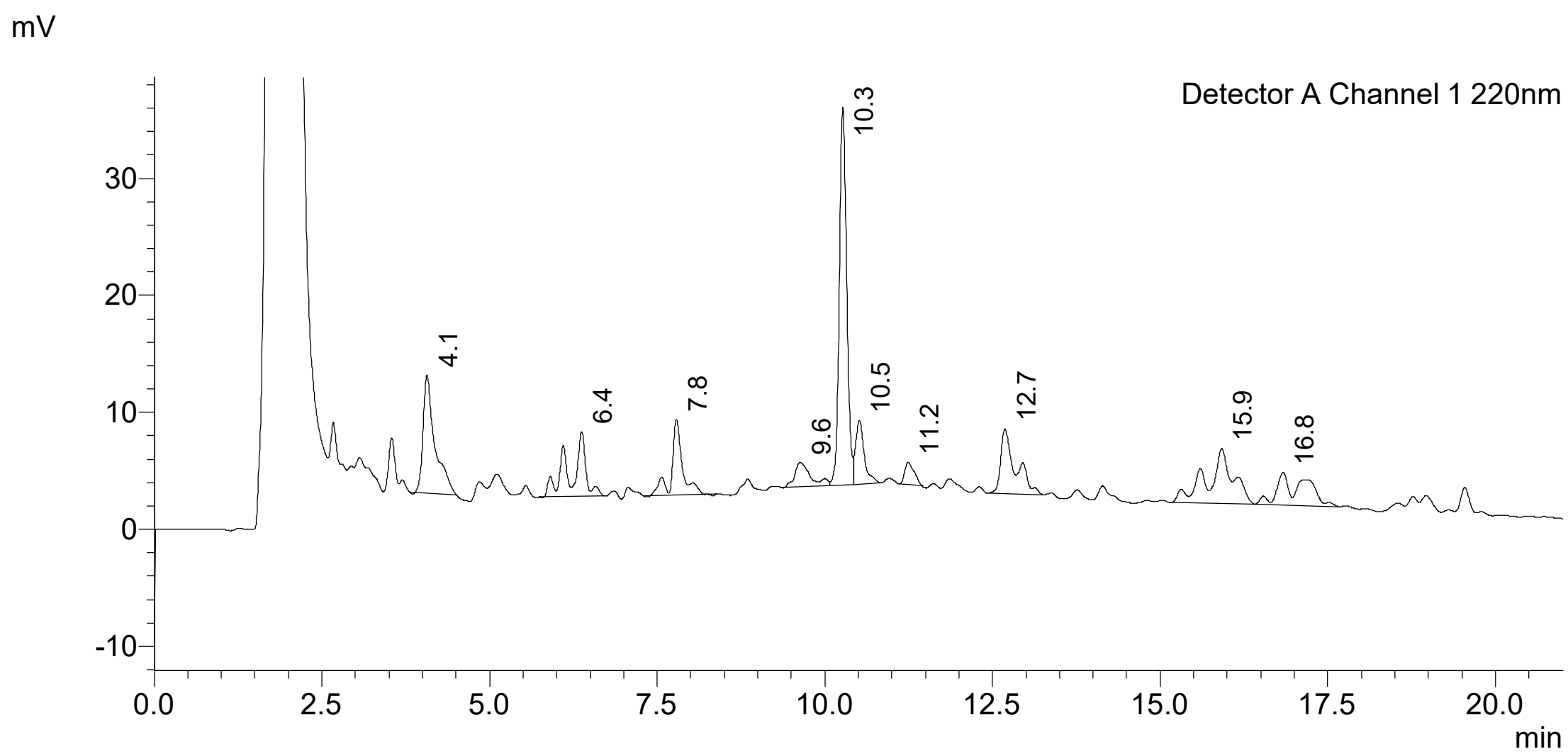

Figure S25: Peptide **20** preparative HPLC purification trace at 220 nm (10.3 min elution; 27% crude purity; 35-65% MeCN over 20 minutes).

Peptide characterisation

Table S2: Purity analysis of synthesised Bak and p53 peptides determined using a Shimadzu LC-2030C 3D analytical HPLC system. A reversed-phase Phenomenex Aeris Peptide XB-C18 column (150 mm x 4.6 mm, 3.6 µM, 100 Å) with a flow rate of 1.5 mL/min. A linear gradient of eluent B (0.08 % (v/v) TFA in MeCN) in eluent A (0.1 % (v/v) TFA in water) over 7.5 or 15 min was used. t_R: Retention time; FAM: 5(6)-carboxyfluorescein; Flu: fluorescein-5-maleimide; Ac: Acetyl; * Stapled with new azobispyrazole cross-linker (**AzBisPy**); ° Stapled with reported azobenzene cross-linker (**AzB**); Purity determined at λ = 220 nm for unstapled peptides; Purity determined using λ = 220 nm and 365 nm for photoswitchable stapled peptides.

Compound	Sequence	t_R (min)	HPLC method (% gradient B, time)	Purity
**9** (Wildtype Bak$^{72-87}$)	Ac-GQVGRQLAIIGDDINR-NH$_2$	3.4	20-95, 15 min	94%
**FAM-9** (FAM-Bak$^{72-87}$)	FAM-GQVGRQLAIIGDDINR-NH$_2$	4.5	20-95, 15 min	95%
**10** ([A^{73},F^{81},C^{77,84}]-Bak$^{72-87}$)	Ac-GAVGR**C**LAI**F**GD**C**INR-NH$_2$	5.4	20-95, 15 min	>98%
**11** ([A^{73},F^{81},C^{77,84}]-Bak$^{72-87}$- **AzB**)	Ac-GAVGR**C°**LAI**F**GD**C°**INR-NH$_2$	3.9	20-95, 15 min	96%
**12** ([A^{73},F^{81},C^{77,84}]-Bak$^{72-87}$- **AzBisPy**)	Ac-GAVGR**C***LAI**F**GD**C***INR-NH$_2$	6.0	25-55, 7.5 min	98%
**13** ([A^{73},F^{81},C^{77,84}]-Bak$^{72-87}$-Arg tag)	Ac-GAVGR**C**LAI**F**GD**C**INR-(Ahx)$_2$-RRRR-NH$_2$	3.7	20-50, 7.5 min	>98%
**14** ([A^{73},F^{81},C^{77,84}]-Bak$^{72-87}$-Arg tag- **AzBisPy**)	Ac-GAVGR**C***LAI**F**GD**C***INR-(Ahx)$_2$-RRRR-NH$_2$	6.2	20-50, 7.5 min	>98%
**15** [A$^{73-74,78,81}$,C^{77,84}]-Bak$^{72-87}$- Arg tag	Ac-G**AA**GR**CA**AI**A**GD**C**INR-(Ahx)$_2$-RRRR-NH$_2$	2.5	20_50, 7.5 min	>98%
**16** [A$^{73-74,78,81}$,C^{77,84}]-Bak$^{72-87}$- Arg tag - **AzBisPy**	Ac-G**AA**GR**C*A**AI**A**GD**C***INR-(Ahx)$_2$-RRRR-NH$_2$	7.4	20-50, 15 min	>98%
**17** (Wildtype p53$^{17-28}$)	Ac-ETFSDLWKLLPE-NH$_2$	4.9	25-55, 7.5 min	>98%
**Flu-18** (Flu-[C^{32}]-p53$^{15-32}$)	Ac-SQETFSDLWKLLPENNVC^{Flu}-NH$_2$	3.9	30-95, 15 min	94%
**19** ([C^{21,28},S^{27}]-p53$^{17-28}$)	Ac-ETFS**C**LWKLLS**C**-NH$_2$	6.4	25-55, 7.5 min	>98%
**20** ([C^{21,28},S^{27}]-p53$^{17-28}$- **AzBisPy**)	Ac-ETFS**C***LWKLLS**C***-NH$_2$	8.9	35-65, 15 min	96%

Table S3: Analysis of the Bak and p53 peptides by LC-MS (ESI$^+$), and Bio LC/QTOF.

Compound	M_{exact} (calc) [Da]	M_W (calc) [Da]	m/z (experimental)	
			ESI$^+$ (LC-MS)	LC/Q-TOF
**9**	1764.9595	1765.9960	1767.4 (m/1), 884.4 (m/2)	883.4612 (m/2)
**FAM-9**	2080.9967	2082.2640	1042.1 (m/2), 695.2 (m/3)	1041.5137 (m/2)
**10**	1704.8552	1706.0200	853.8 (m/2)	853.4087 (m/2)
**11**	2156.8629	2158.4320	1080.2 (m/2)	1079.4475 (m/2)
**12**	2099.0458	2100.5020	701.1 (m/3)	700.6889 (m/3)
**13**	2555.4278	2557.0960	853.2 (m/3), 640.2 (m/4)	1278.7202 (m/2)

**14**	2949.6184	2951.5780	984.8 (m/3), 738.8 (m/4)	1475.8213 (m/2)
**15**	2409.3182	2410.8630	804.3 (m/3), 603.6 (m/4)	804.1354 (m/3)
**16**	2803.5088	2805.3450	935.9 (m/3), 702.3 (m/4)	935.5148 (m/3)
**17**	1517.7766	1518.7320	760.1 (m/2)	759.8733 (m/2)
**Flu-18**	2590.0999	2591.7890	-	864.3848 (m/3)
**19**	1469.7047	1470.7680	736.0 (m/2)	734.8525 (m/2)
**20**	1863.8953	1865.2500	933.6 (m/2)	1864.8977 (m/1)

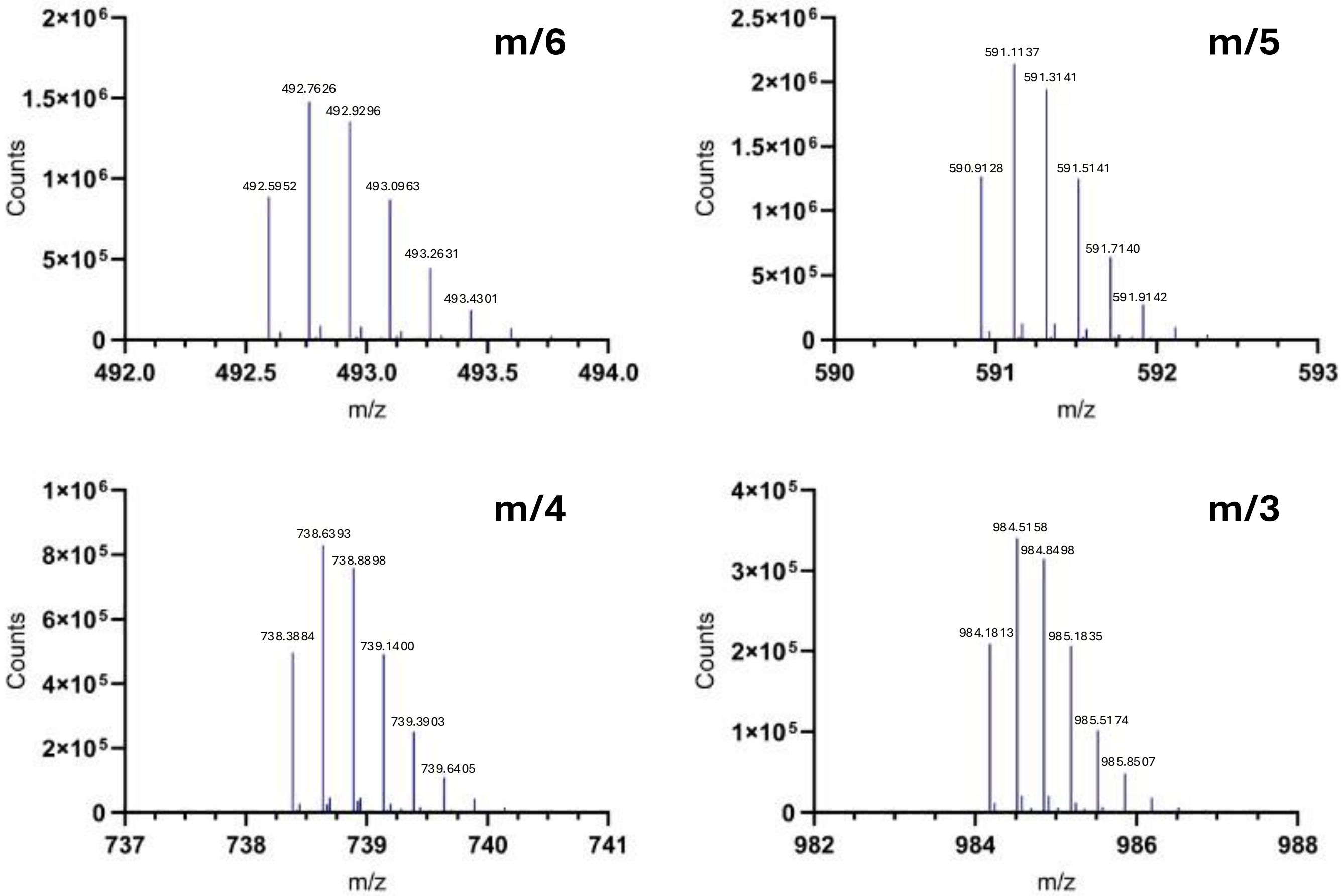

Figure S26: Bio LC/Q-TOF isotopic distribution of peptide **14** confirming the m/3, m/4, m/5 and m/6 charge states of the stapled peptide (one peptide connected via a single linker).

Analytical HPLC traces of purified Bak and p53 peptides:

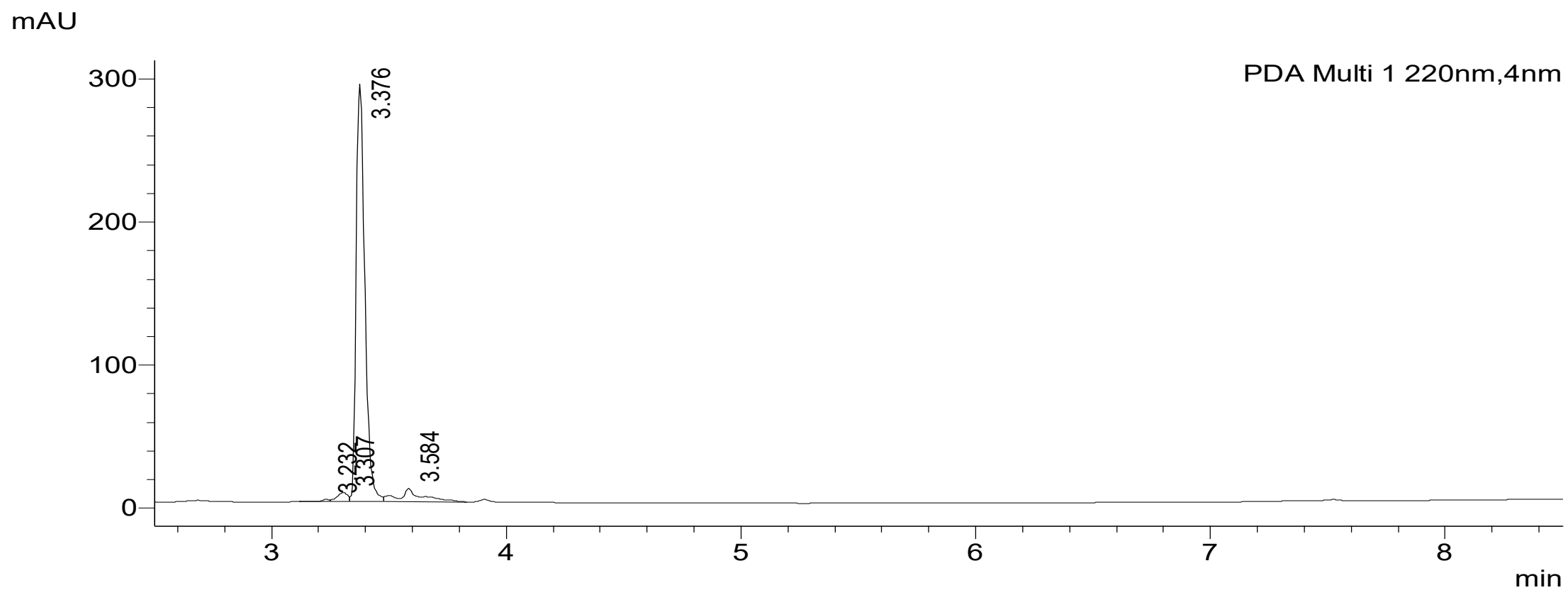

Figure S27: Peptide **9** (Wildtype Bak$^{72-87}$) at 220 nm.

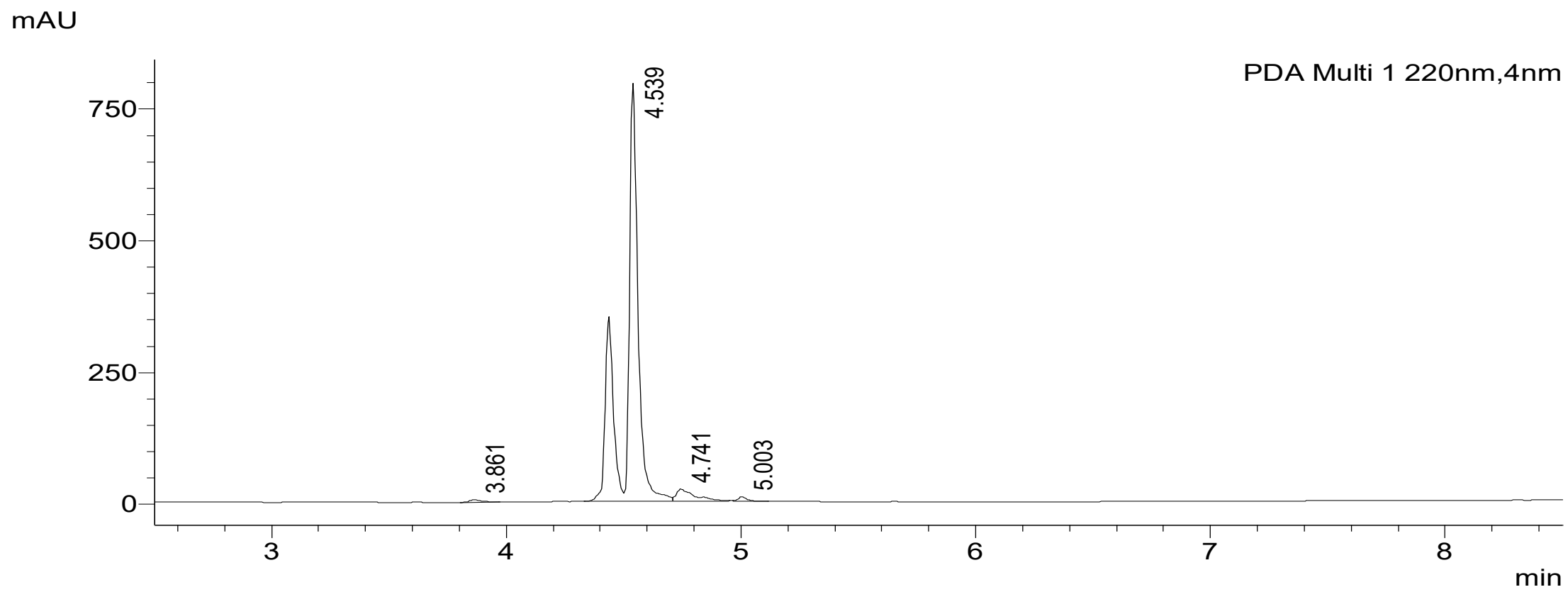

Figure S28: Peptide **FAM-9** (FAM-Bak$^{72-87}$) at 220 nm – Both FAM isomers present

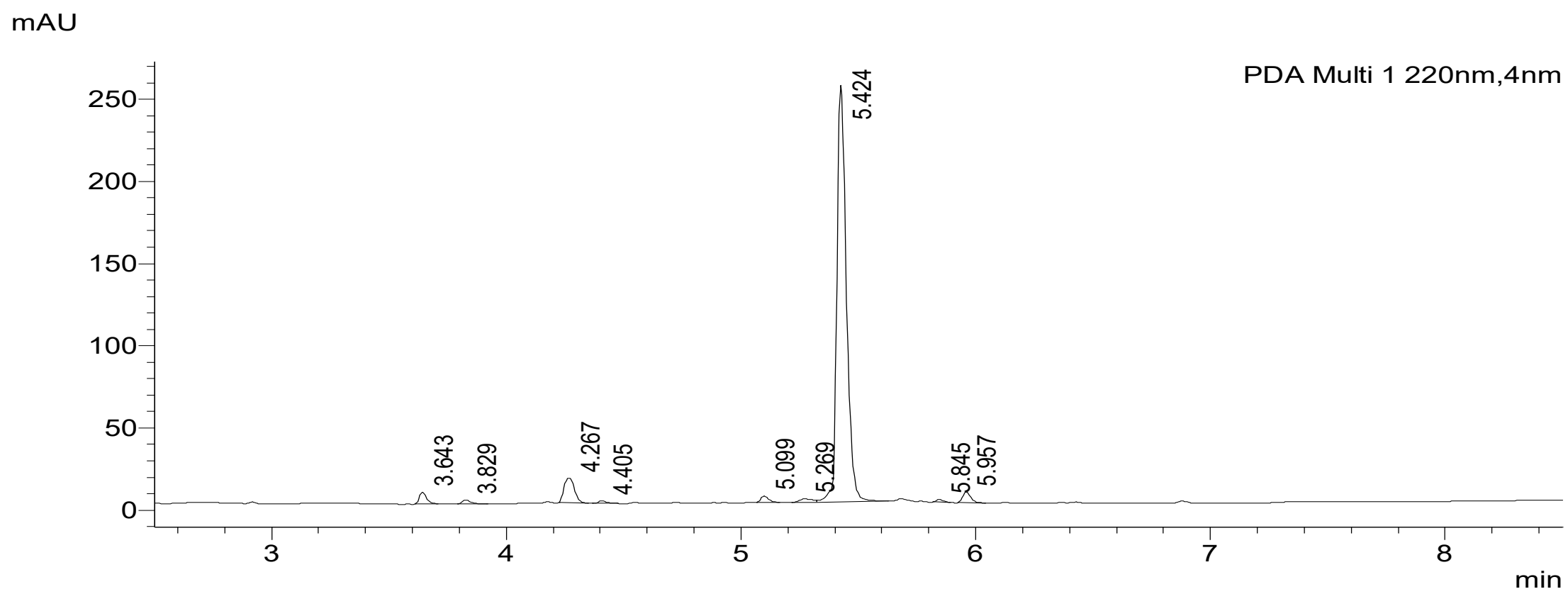

Figure S29: Peptide **10** ([A^{73},F^{81},C^{77,84}]-Bak$^{72-87}$) at 220 nm

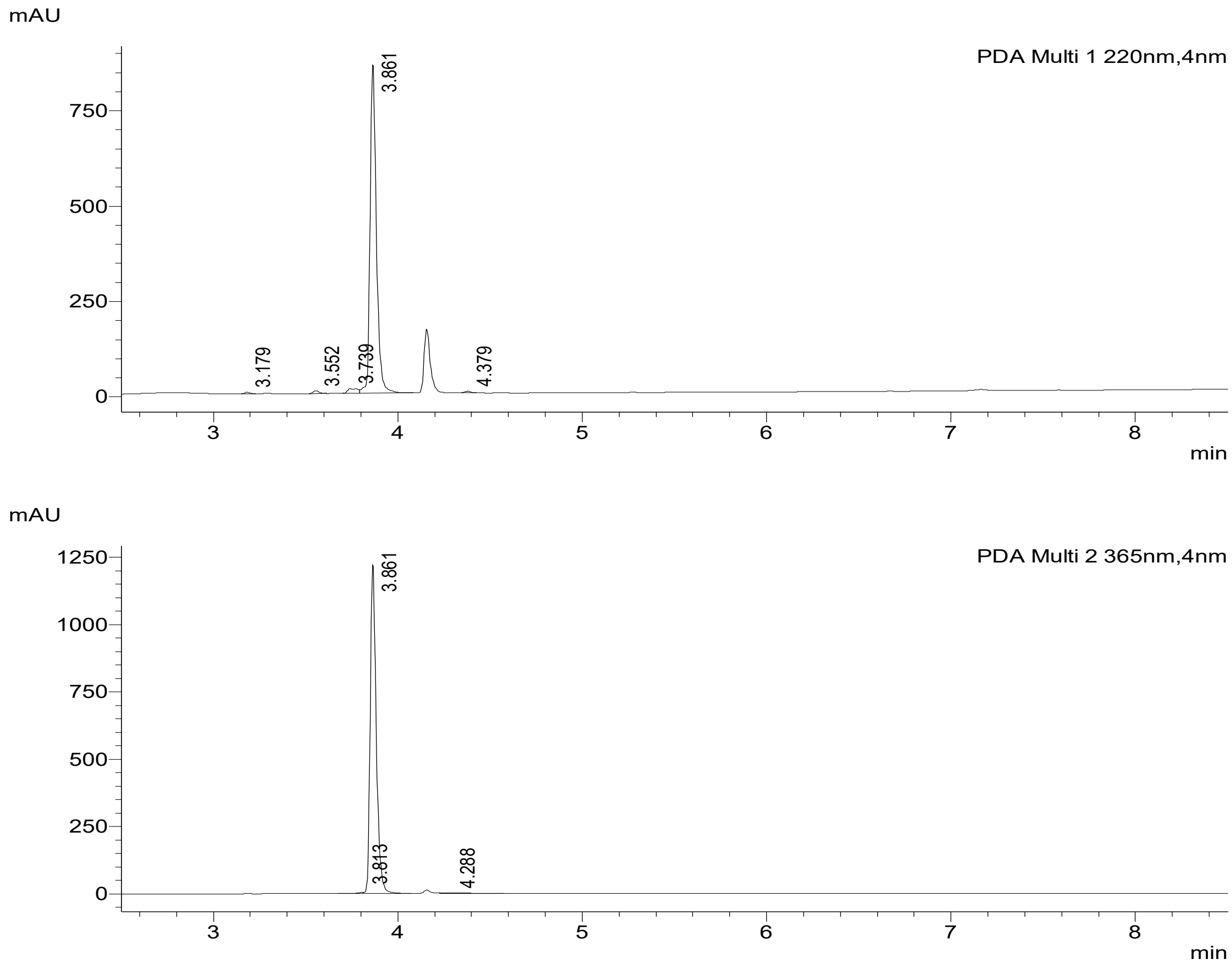

Figure S30: Peptide **11** ([A^{73},F^{81},$C^{77,84}$]-$Bak^{72\text{-}87}$-AzB) at 220 nm and 365 nm – Peak at 4.155 min was confirmed to be peptide **11** *Z* isomer

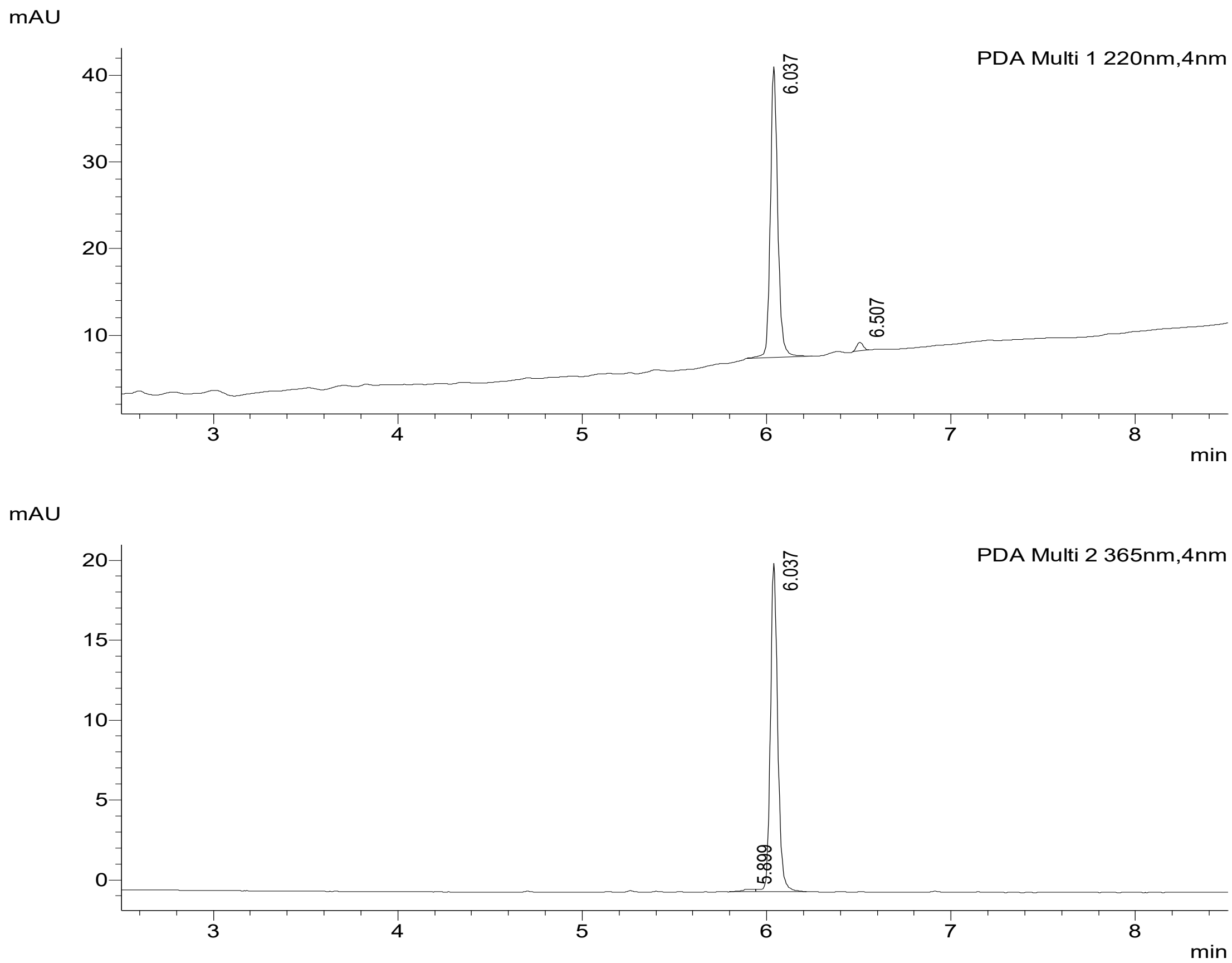

Figure S31: Peptide **12** ([A^{73},F^{81},$C^{77,84}$]-$Bak^{72\text{-}87}$-AzBisPy) at 220 nm and 365 nm

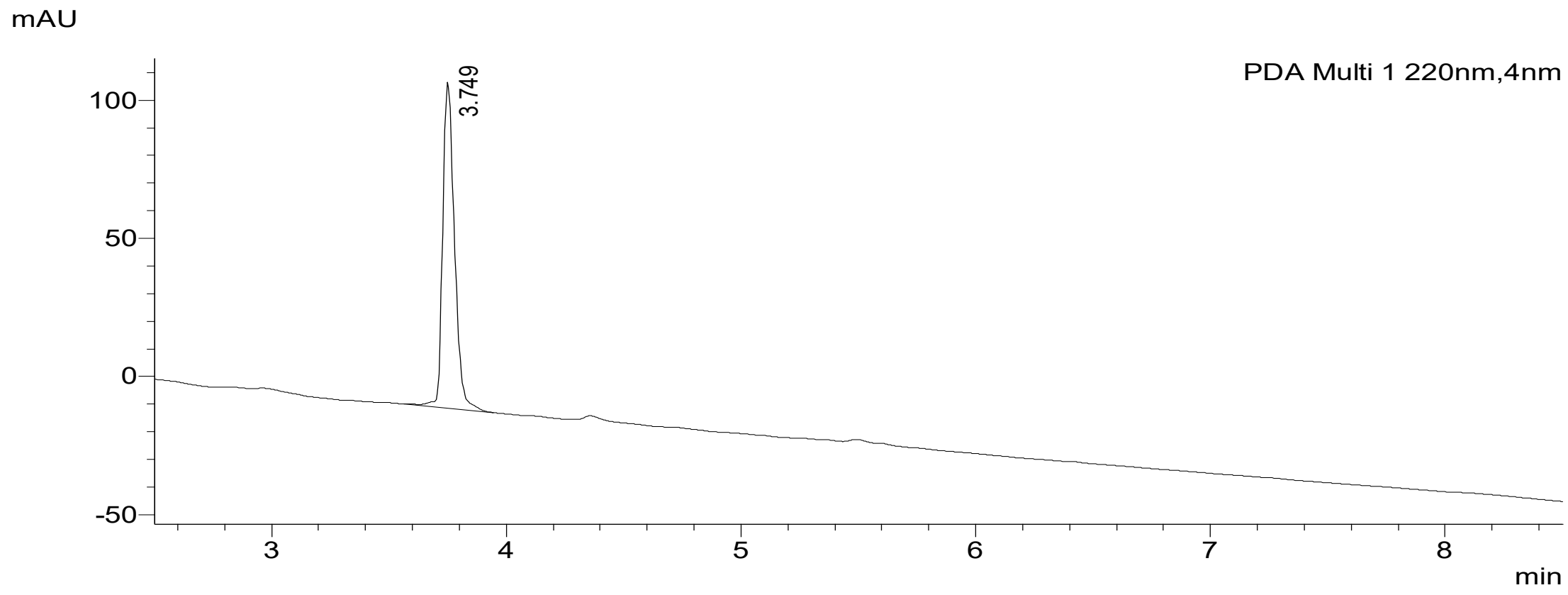

Figure S32: Peptide **13** ([A^{73},F^{81},C^{77,84}]-Bak$^{72\text{-}87}$-Arg tag) at 220 nm

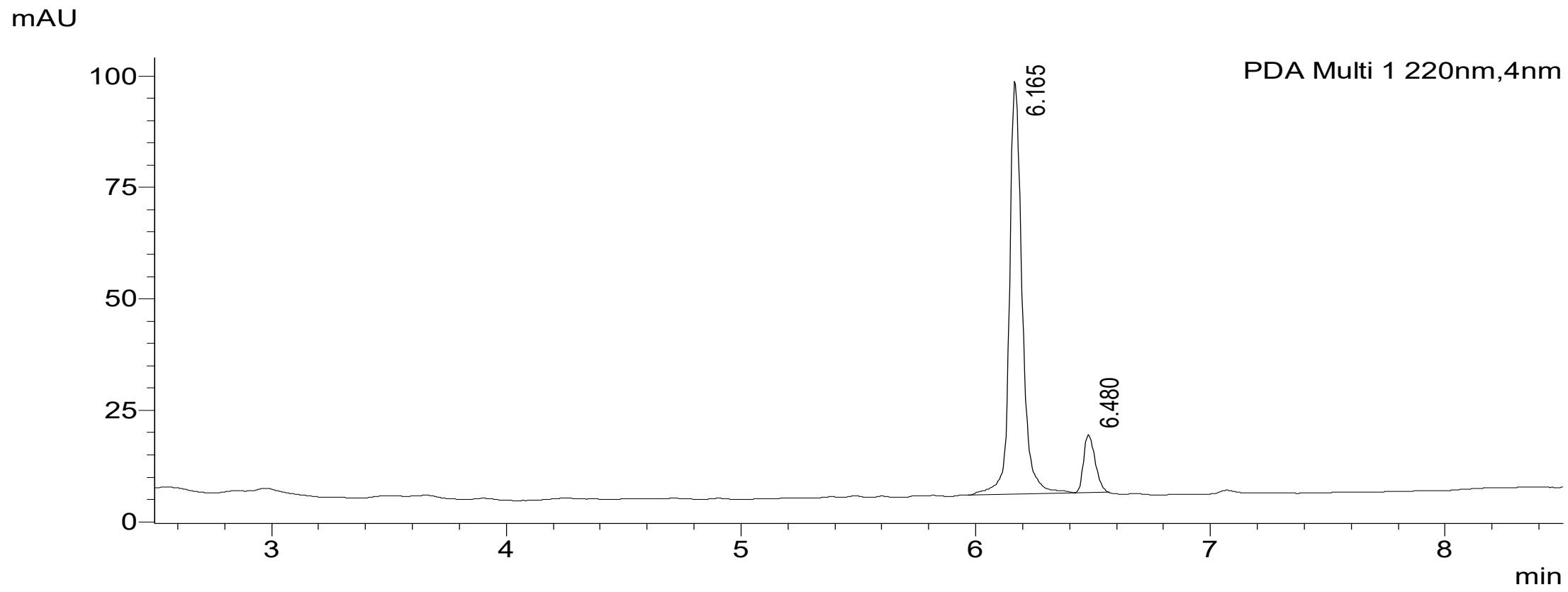

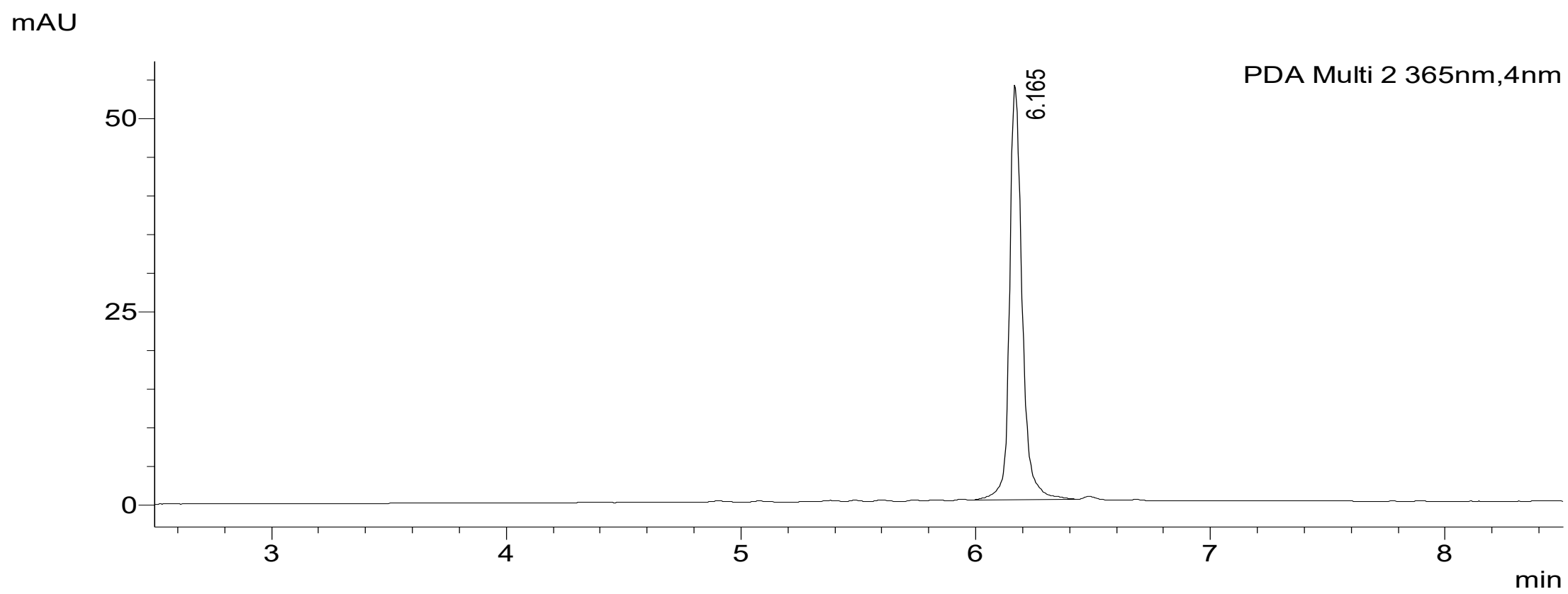

Figure S33: Peptide **14** ([A^{73},F^{81},C^{77,84}]-Bak$^{72\text{-}87}$-Arg tag-AzBisPy) at 220 nm and 365 nm – Peak at 6.480 min was confirmed to be peptide **14** *Z* isomer

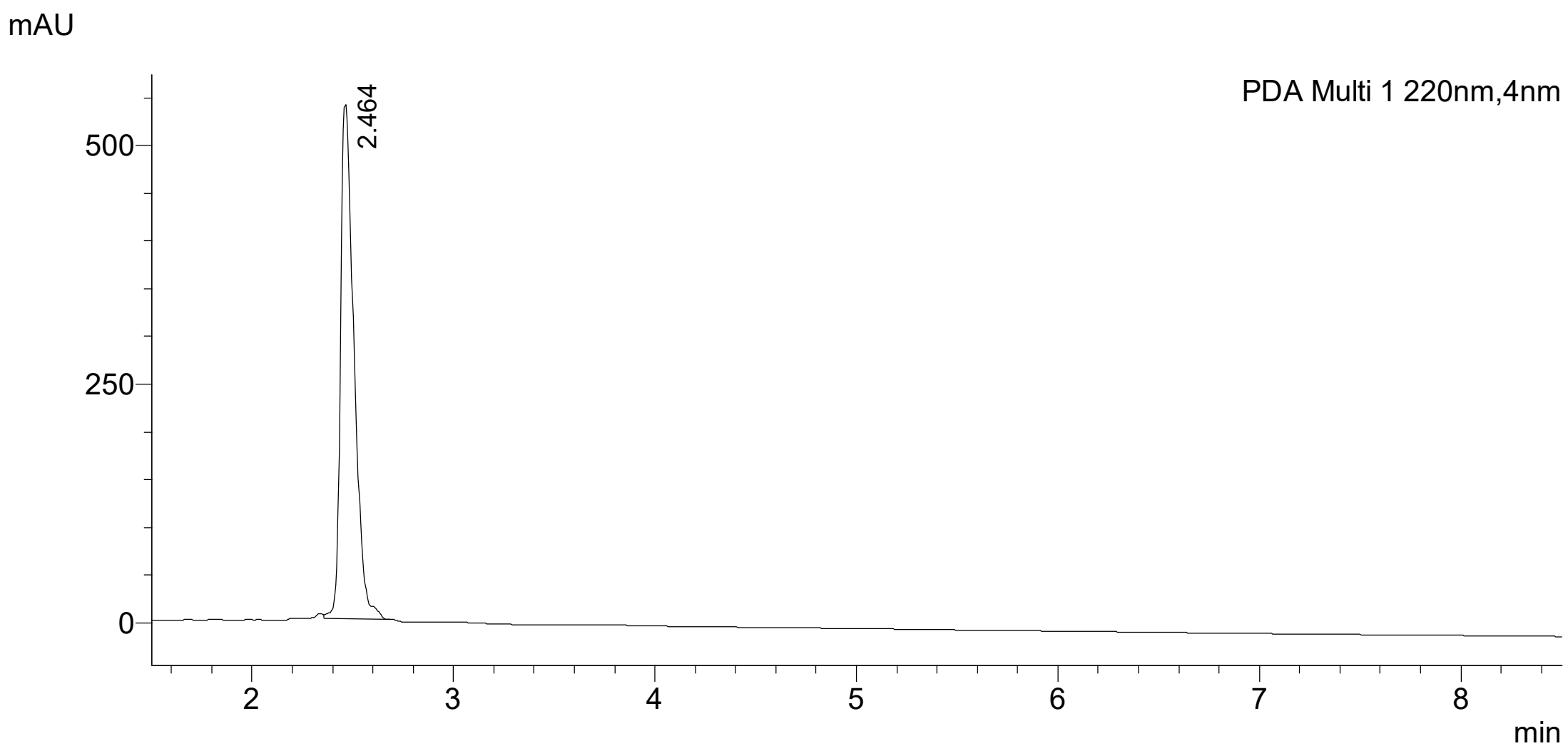

Figure S34: Peptide **15** ([A$^{73\text{-}74,78,81}$,C^{77,84}]-Bak$^{72\text{-}87}$-Arg tag) at 220 nm

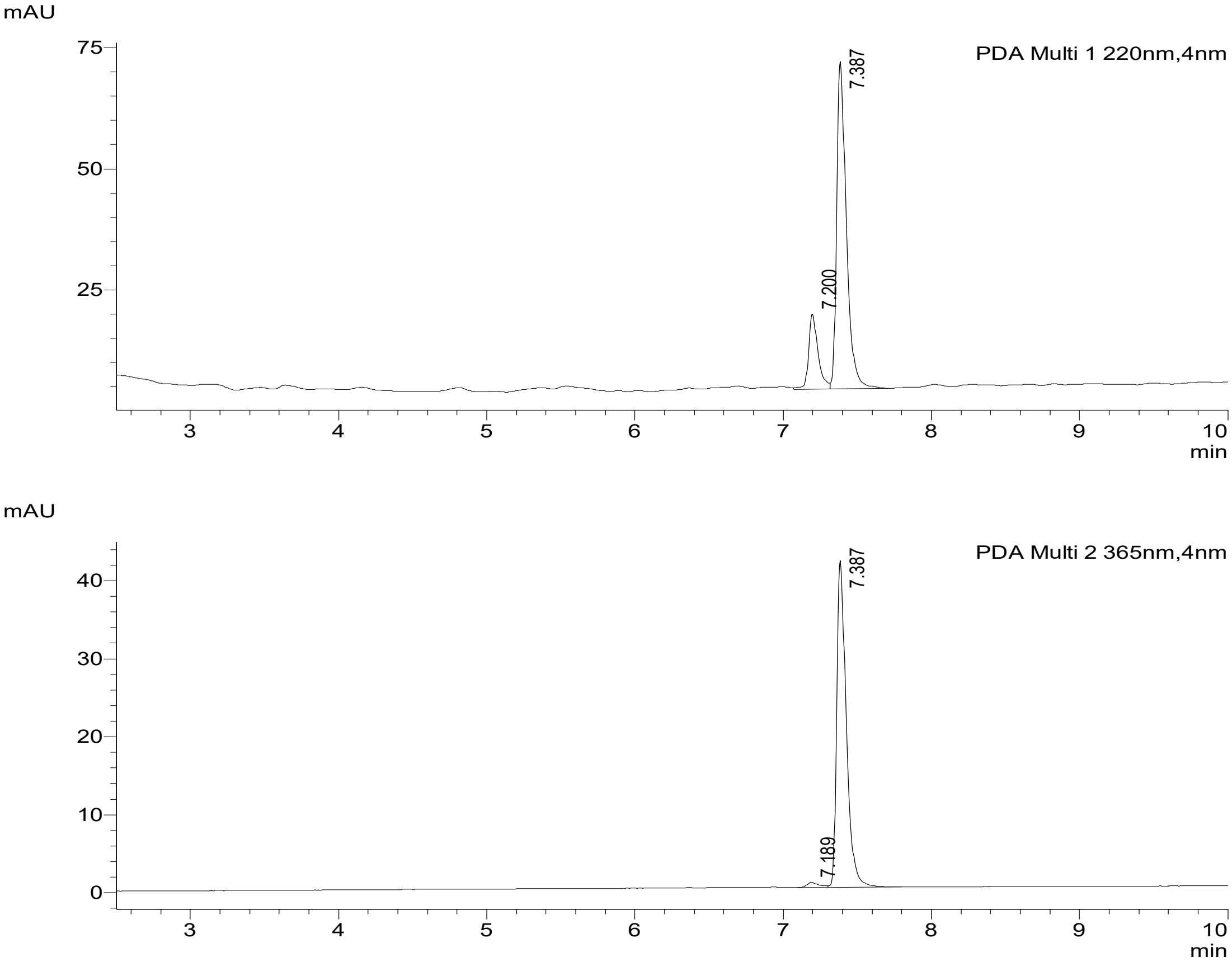

Figure S35: Peptide **16** ([A$^{73\text{-}74,78,81}$,C^{77,84}]-Bak$^{72\text{-}87}$-Arg tag-AzBisPy) at 220 nm and 365 nm – Peak at 7.200 min was confirmed to be peptide **16** *Z* isomer

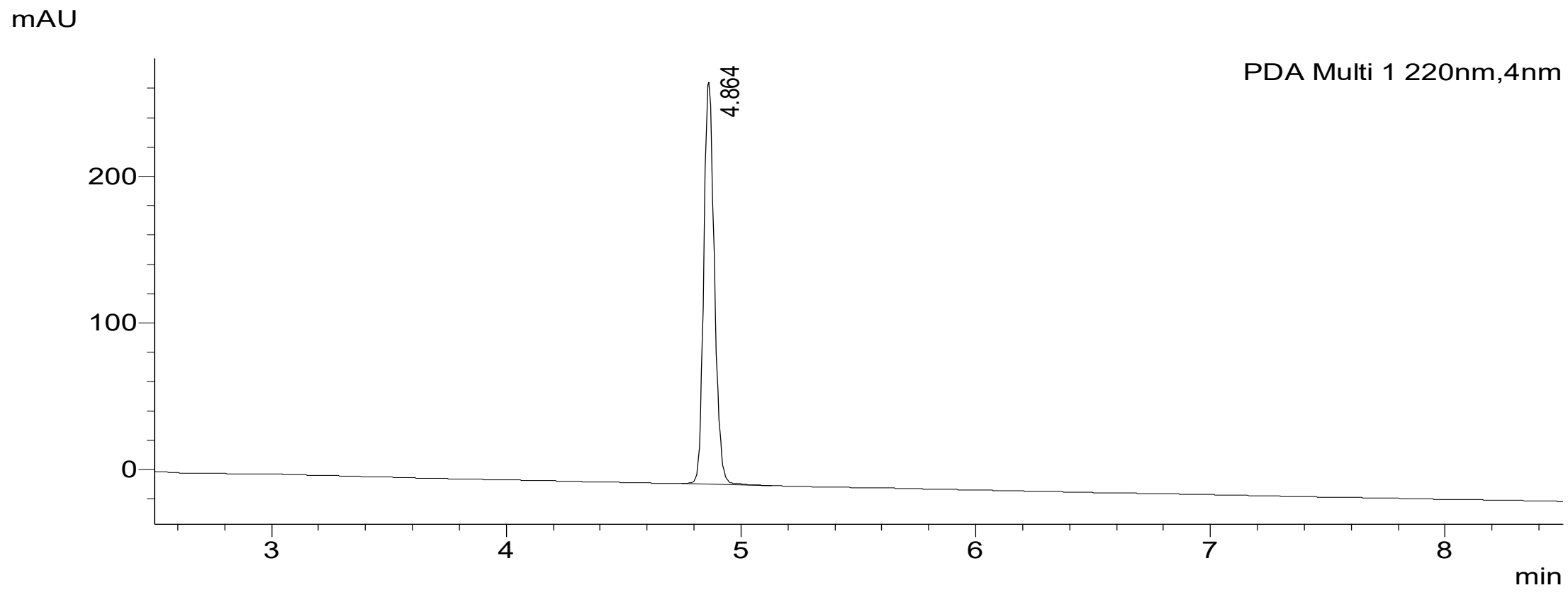

Figure S36: Peptide **17** (Wildtype p53$^{17-28}$) at 220 nm

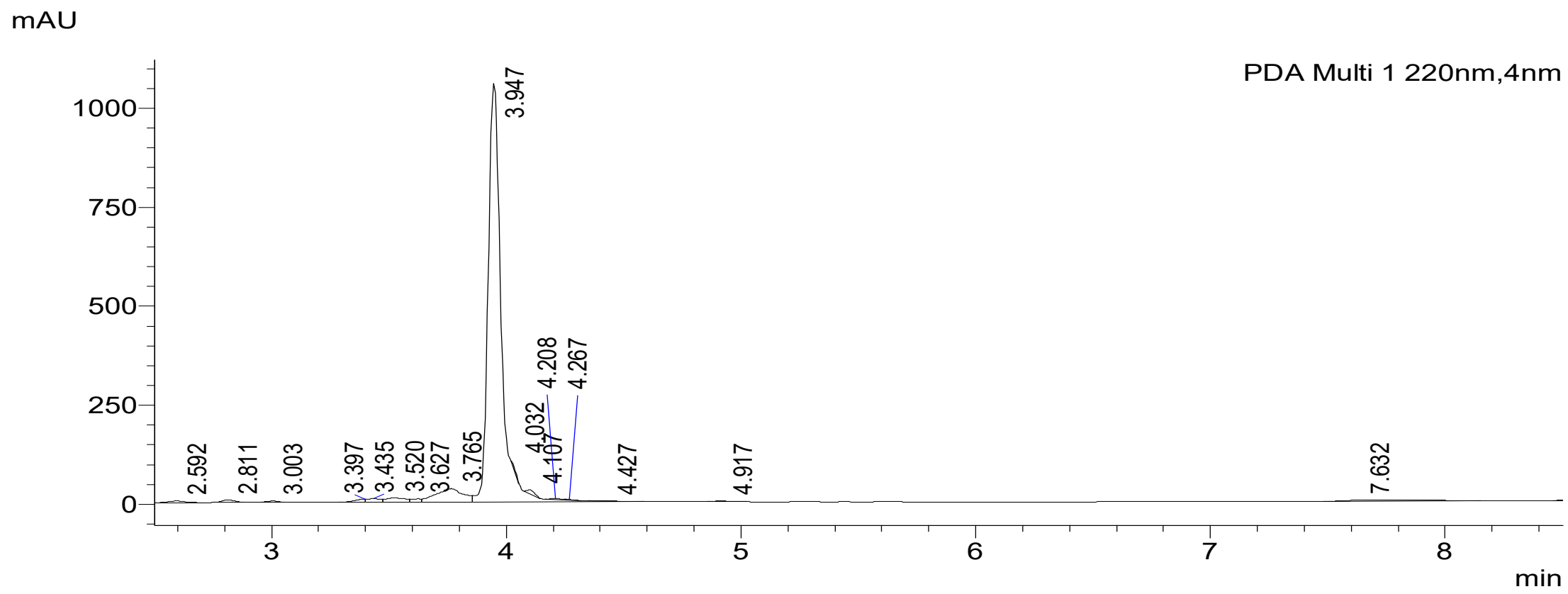

Figure S37: Peptide **Flu-18** (Flu-[C^{32}]-p53$^{15-32}$) at 220 nm

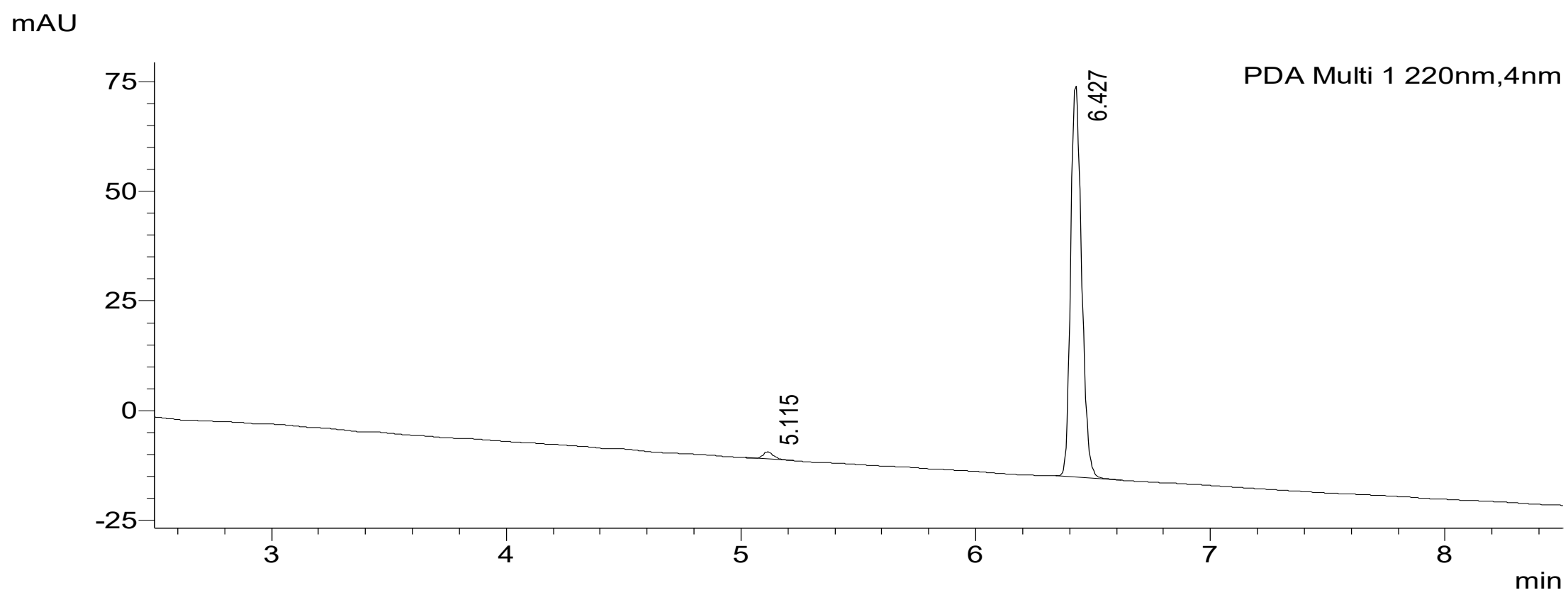

Figure S38: Peptide **19** ([C^{21,28},S^{27}]-p53$^{17-28}$) at 220 nm

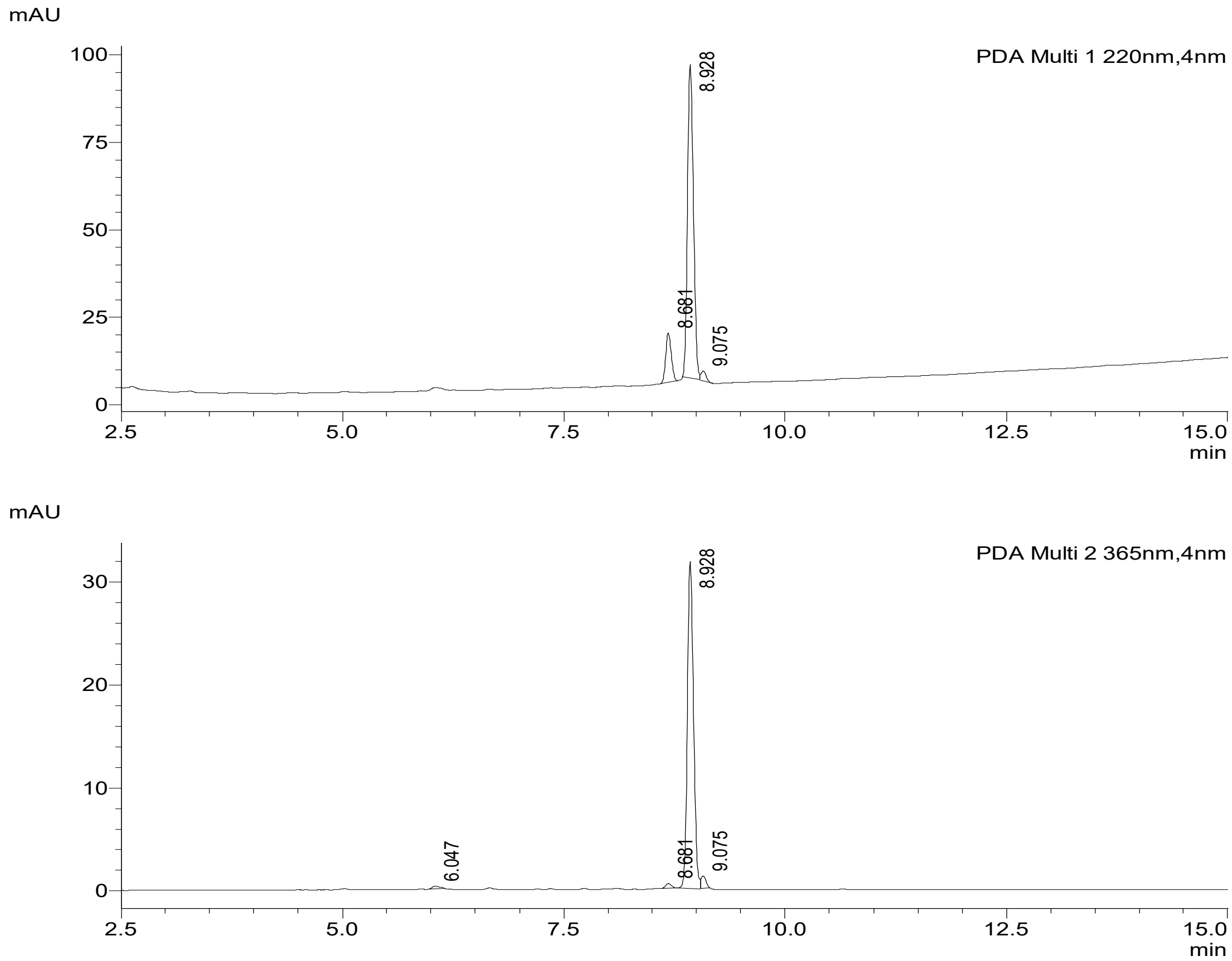

Figure S39: Peptide **20** ([$C^{21,28}$,S^{27}]-$p53^{17-28}$-AzBisPy) at 220 nm and 365 nm – Peak at 8.681 was confirmed to be peptide **20** *Z* isomer

Protein expression

Molecular Cloning of Bcl-x_L^{1-209}(Δ45-84) construct

The C-terminally truncated human Bcl-x_LΔC gene construct (residues 1-209, Uniprot ID of full-length Bcl-x_L: Q07817-1) was purchased from Integrated DNA Technologies (Coralville, IA, USA). To generate the construct Bcl-x_L^{1-209}(Δ45-84), the internal loop sequence (residues 45-84) was deleted from Bcl-x_L^{1-209} by overlap extension PCR. The sequences of the Bcl-x_L gene constructs and the used primer are shown in Tables S4 and S5, respectively. The PCR-amplified insert Bcl-x_L^{1-209}(Δ45-84) was cloned into a pET24a vector, coding for a C-terminal 6x His-tag, using Nde1 (5' end) and Xho1 (3' end) restriction sites and standard DNA ligation. The correct identity of the insert in the generated pET24a_Bcl-x_L^{1-209}(Δ45-84) plasmid was confirmed by DNA sequencing.

Expression and purification of Bcl-x_L^{1-209}(Δ45-84)

Competent *E. coli* BL21 cells were transformed with pET24a_Bcl-x_L^{1-209}(Δ45-84) plasmid using the standard heat shock method. Transformed BL21 cells (50 µL) were diluted in LB media (10 mL) supplemented with kanamycin (50 µg/mL) and chloramphenicol (34 µg/ml) and grown overnight at 37°C. The whole cell suspension was added into fresh LB media (1 L) supplemented with kanamycin (50 µg/mL) and chloramphenicol (34 µg/ml) and grown for 3 hours at 37°C until the bacterial culture reached OD_{600} of ~ 1.0. IPTG (1 mM) was added, the culture was grown overnight at 18°C and bacteria were subsequently harvested by centrifugation (4000 rpm, 30 min, 4°C). Bacterial pellets were resuspended in cold immobilized metal affinity chromatography (IMAC) binding buffer (50 mM Tris (pH 8.0), 300 mM NaCl, 20 mM imidazole) supplemented with EDTA-free protease inhibitor cocktail (Roche, 1 tablet per 50 mL of lysate)

and DNAse I (Sigma-Aldrich, 1 mg/ml) and cells were lysed by sonication on ice for 3 min using 1 s bursts. The lysate was cleared by centrifugation (20,000 rpm, 45 min, 4°C) and the supernatant was syringe-filtered (0.45 µm filter). The filtered protein solution was loaded onto a Ni^{2+}-NTA resin affinity column (1 ml bead loading) that was pre-equilibrated with IMAC binding buffer. The loaded column was first washed with IMAC binding buffer, followed by 10% (v/v) IMAC elution buffer (50 mM Tris (pH 8.0), 300 mM NaCl, 250 mM imidazole) in IMAC binding buffer. Bound proteins were eluted with IMAC elution buffer until negligible amounts of protein (0.01 mg/mL) were detected in the eluent. Fractions containing Bcl-$x_L^{1\text{-}209}$(Δ45-84) were combined and further purified by size-exclusion chromatography (SEC) on a HiLoad 16/600 Superdex 75pg column (GE Healthcare) using an ÄKTA prime plus system. Proteins were separated by isocratic elution with SEC running buffer (50 mM Tris (pH 8.0), 200 mM NaCl, 5% glycerol) and a flow rate of 1.5 mL/min. SEC fractions containing Bcl-$x_L^{1\text{-}209}$(Δ45-84) were combined, concentrated to around 200 µM, flash frozen in liquid nitrogen and stored at -80 °C. The purity of Bcl-$x_L^{1\text{-}209}$(Δ45-84) was analysed by SDS-PAGE (> 95%) and the yield of the purification was approximately 0.7 mg protein per litre of culture.

Table S4: DNA sequences of Bcl-x_L gene constructs (5’ to 3’ direction). The internal loop sequence (45-84) is marked in dark red.

**Bcl-$x_L^{1\text{-}209}$**
ATGTCCCAATCCAATCGCGAACTTGTAGTTGATTTCTTGTCTTACAAATTATCTCAAAAGGGATATTCTTGGTCCCAATTCAGCG ACGTCGAGGAAAACCGTACAGAAGCGCCAGAAGGGACTGAATCGGAGATGGAAACACCGTCCGCGATCAACGGGAATCCCT CTTGGCACCTTGCCGACAGCCCGGCGGTTAACGGTGCTACTGGACACTCTAGTAGCTTAGATGCTCGCGAGGTAATCCCAATG GCAGCGGTCAAACAAGCACTGCGCGAGGCAGGGGATGAATTTGAGCTGCGCTACCGTCGCGCCTTTTCCGACCTTACCTCCC AACTTCACATTACTCCCGGCACTGCGTACCAGTCTTTTGAGCAGGTTGTGAATGAACTTTTCCGTGATGGAGTTAATTGGGGG CGCATTGTAGCCTTCTTCTCATTTGGGGGAGCCCTGTGCGTTGAGTCGGTTGACAAGGAGATGCAGGTCCTGGTCTCGCGTAT CGCAGCGTGGATGGCAACGTACCTTAATGACCATTTAGAACCGTGGATCCAGGAGAATGGTGGCTGGGATACGTTCGTCGAG CTGTACGGGAATAACGCTGCAGCAGAGTCTCGTAAGGGCCAAGAACGC
**Bcl-$x_L^{1\text{-}209}$(Δ45-84)**
ATGTCCCAATCCAATCGCGAACTTGTAGTTGATTTCTTGTCTTACAAATTATCTCAAAAGGGATATTCTTGGTCCCAATTCAGCG ACGTCGAGGAAAACCGTACAGAAGCGCCAGAAGGGACTGAATCGGAGGCGGTCAAACAAGCACTGCGCGAGGCAGGGGAT GAATTTGAGCTGCGCTACCGTCGCGCCTTTTCCGACCTTACCTCCCAACTTCACATTACTCCCGGCACTGCGTACCAGTCTTTTG AGCAGGTTGTGAATGAACTTTTCCGTGATGGAGTTAATTGGGGGCGCATTGTAGCCTTCTTCTCATTTGGGGGAGCCCTGTGC GTTGAGTCGGTTGACAAGGAGATGCAGGTCCTGGTCTCGCGTATCGCAGCGTGGATGGCAACGTACCTTAATGACCATTTAG AACCGTGGATCCAGGAGAATGGTGGCTGGGATACGTTCGTCGAGCTGTACGGGAATAACGCTGCAGCAGAGTCTCGTAAGG GCCAAGAACGC

Table S5: Primer sequences used for molecular cloning of the Bcl-$x_L^{1\text{-}209}$(Δ45-84) construct. Restrictions sites are underlined.

Primer	Sequence
Bcl-x_L_Nde1_fw	GGTGGTCATATGTCCCAATCCAATCGCG
Bcl-x_L_Xho1_rv	CCATTACTCGAGGCGTTCTTGGCCC
Del45-84_fw	GGGACTGAATCGGAGGCGGTCAAACAAGC
Del45-84_rv	GCTTGTTTGACCGCCTCCGATTCAGTCCC

Protein expression of hDM2 (17-125)

A pet28a His_6–hDM2 (17-125) L33E construct (provided by Professor Andy Wilson) was used for transformation, with protein expression carried out as previously described[2, 3] by Dr. Amrita Date and Dr. Archie Wall.

Compound preparation and characterisation

3,5-dimethyl-1*H*-pyrazol-4-amine (2)

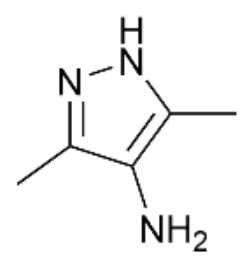

3,5-dimethyl-4-nitro-1*H*-pyrazole (1.00 g, 7.09 mmol, 1.0 eq.) was dissolved in methanol (90 mL) under nitrogen. The solution was added dropwise to a separate flask containing Pd/C (0.100 g, 10% w/w) under nitrogen. The reaction mixture was degassed with hydrogen for 20 minutes and then stirred under a positive pressure of hydrogen for 44 hours at room temperature. Once complete, the reaction mixture was filtered over celite and washed with DCM. The filtrate was concentrated *in vacuo* to afford **2** as a pale yellow powder (754 mg, 6.78 mmol, 96%). Rf (TLC) 0.30 (5% MeOH/DCM); ^{1}H NMR (400 MHz, DMSO-d_6) δH 11.42 (s, 1H), 3.23 (s, 2H), 1.99 (s, 6H) ppm; ^{13}C NMR (101 MHz, DMSO-d_6) δC 123.9, 9.8 ppm. ^{1}H NMR shifts match those reported in literature.[4]

3-(2-(3,5-dimethyl-1*H*-pyrazol-4-yl)hydrazineylidene)pentane-2,4-dione (3)

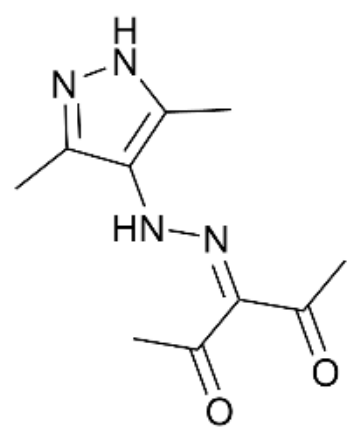

3,5-dimethyl-1*H*-pyrazol-4-amine **(2)** (700 mg, 6.30 mmol, 1.0 eq.) was dissolved in a mixture of water (8.9 mL) and MeOH (7.2 mL). Concentrated HCl (1.5 mL) was added dropwise at 0°C. A solution of sodium nitrite (522 mg, 7.56 mmol, 1.2 eq.) in water (4.5 mL) was added dropwise to the reaction mixture and then stirred at 0 °C for 2 hours.

In a separate flask, potassium acetate (5.56 g, 56.7 mmol, 9.0 eq.) and acetyl acetone (776 µL, 7.56 mmol, 1.2 eq.) were dissolved in water (18 mL) and cooled to 0 °C. The initial reaction mixture was added dropwise to the second flask while stirring at 0°C and was left to stir at room temperature for 2 hours. The precipitate was filtered, washed with water and dried *in vacuo* to afford **3** as a bright yellow solid (825 mg, 3.71 mmol, 86%). Rf (TLC) 0.45

(EtOAc/hexane 8:2); ^{1}H NMR (400 MHz, DMSO-d_6) δH 15.01 (s, 1H), 12.54 (s, 1H), 2.44 (s, 3H), 2.27 (pseudo-s, 9H) ppm; ^{13}C NMR (101 MHz, DMSO-d_6) δC 195.7, 132.4, 120.8, 30.9, 26.8, 10.8 ppm. 1H NMR shifts match those reported in literature.[5]

(*E*)-1,2-bis(3,5-dimethyl-1*H*-pyrazol-4-yl)diazene (4)

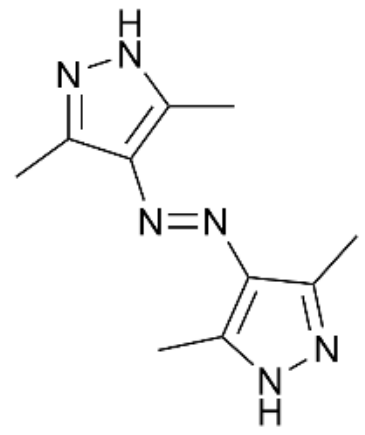

3-(2-(3,5-dimethyl-1*H*-pyrazol-4-yl)hydrazineylidene)-pentane-2,4-dione (**3**) (600 mg, 2.70 mmol, 1.0 eq.) was added to acetic acid (8.0 mL). Hydrazine monohydrate (168 µL, 3.24 mmol, 1.2 eq.) was added dropwise then heated to 90°C overnight. Following reaction completion, the mixture was cooled to room temperature and filtered and the precipitate was washed with water. The crude product was recrystallised from ethanol and water and the product was dried *in vacuo* to afford **4** as a bright yellow solid (400 mg, 1.83 mmol, 68%). Rf (TLC) 0.50 (100% EtOAc); ^{1}H NMR (400 MHz, DMSO-d_6) δH 12.55 (s, 2H), 2.41 (s, 6H), 2.32 (s, 6H) ppm; ^{13}C NMR (101 MHz, DMSO-d_6) δC 141.5, 135.5, 134.4, 13.6, 9.9 ppm. ^{1}H NMR shifts match those reported in literature.[6]

(4-(((*tert*-butyldimethylsilyl)oxy)methyl)phenyl)boronic acid (5)

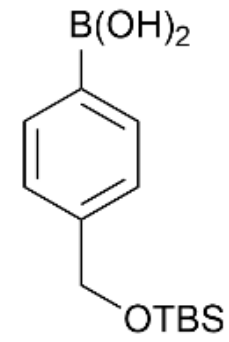

Tert-butyldimethylsilyl chloride (TBSCl) (6.94 g, 46.1 mmol, 3.5 eq.) was added dropwise to imidazole (4.48 g, 65.8 mmol, 5.0 eq.) and (4-(hydroxymethyl)phenyl)boronic acid (2.00 g, 13.2 mmol, 1.0 eq.) in anhydrous DMF (100 mL). The reaction mixture was stirred at room temperature for 21 hours and then was diluted with EtOAc (320 mL) and water (160 mL). The organic phase was separated and washed with water (3 x 100 mL). The aqueous phases were combined and washed with EtOAc (2 x 200 mL). The combined organic phases were washed with brine (2 x 200 mL), dried over $MgSO_4$ and concentrated *in vacuo*. The crude product was purified by column chromatography (0-50% ethyl acetate in hexane) to afford **5** as a white solid (2.19 g, 8.22 mmol, 62%). Rf (TLC) 0.54 (EtOAc/hexane 1:1); ^{1}H NMR (400 MHz, $CDCl_3$) δH 8.21 (d, 2H, *J* = 7.8 Hz), 7.47 (d, 2H, *J* = 7.8 Hz), 4.84 (s, 2H), 0.97 (s, 9H), 0.13 (s, 6H) ppm; ^{13}C NMR (101 MHz, $CDCl_3$) δC 146.4, 135.8, 125.6, 65.1, 26.1, 18.6, -5.1 ppm. ^{1}H NMR shifts match those reported in literature.[7]

(*E*)-1,2-bis(1-(4-(((tert-butyldimethylsilyl)oxy)methyl)phenyl)-3,5-dimethyl-1*H*-pyrazol-4-yl)diazene (6)

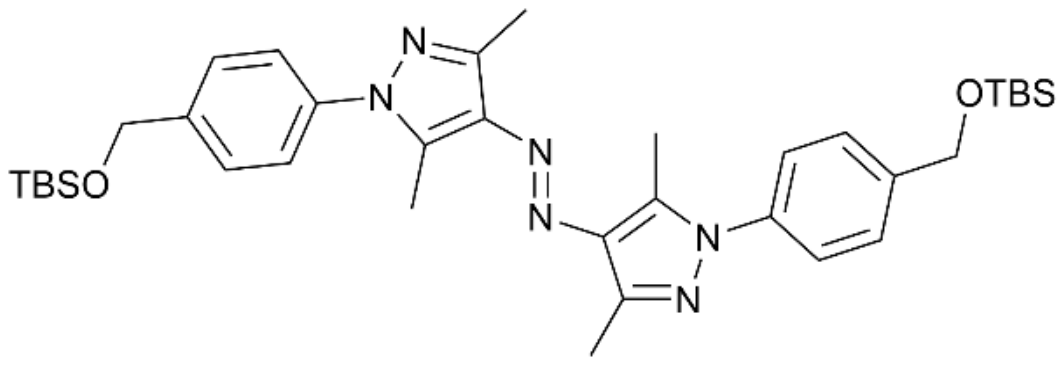

To a flask with 4Å molecular sieves was added: (4-(((*tert*butyldimethylsilyl)oxy)methyl)phenyl)boronic acid (**5**) (1.22 g, 4.58 mmol, 4.0 eq.), 1,2-bis(3,5-dimethyl-1*H*-pyrazol-4-yl)diazine (**4**) (250 mg, 1.15 mmol, 1.0 eq.), copper (II) acetate (624 mg, 3.44 mmol, 3.0 eq.) in anhydrous DMF (37 mL). Pyridine (367 µL, 4.58 mmol, 4.0 eq.) was added and the reaction was stirred at room temperature for 23 hours. The reaction mixture was diluted with DCM and filtered

through celite. The resulting DCM filtrate was washed with water (3 x 70 mL). The organic phase was washed with 5% LiCl (2 x 100 mL) and brine (2 x 100 mL), dried over $MgSO_4$ and concentrated *in vacuo*. The crude product was purified by flash column chromatography (0-20% ethyl acetate in hexane) to afford **6** as a bright yellow powder. An unknown impurity remained following column chromatography – the crude product was taken into the next step without further purification.

(*E*)-((diazene-1,2-diylbis(3,5-dimethyl-1*H*-pyrazole-4,1-diyl))bis(4,1-phenylene))dimethanol (7)

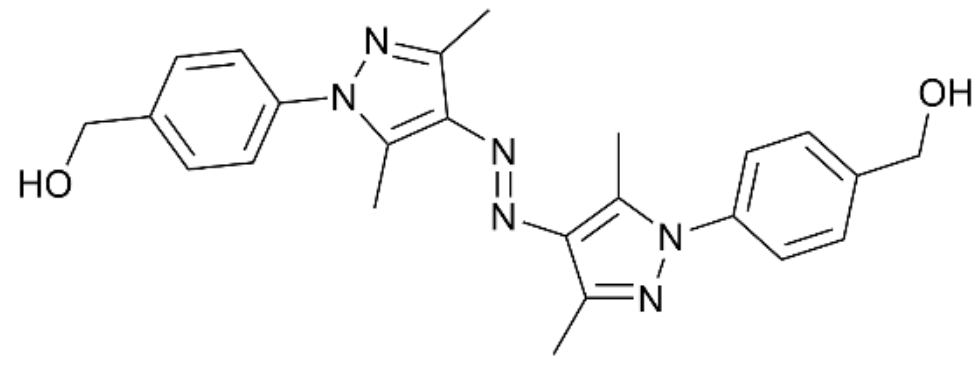

The crude product **6** (800 mg, 1.06 mmol assumed, 1.0 eq.) was dissolved in anhydrous THF (40 mL) under nitrogen. A 1M solution of tetrabutylammonium fluoride (TBAF) in THF (2.33 mL, 2.34 mmol, 2.2 eq.) was added dropwise. The reaction mixture was stirred under nitrogen at room temperature for 1 hour. The solvent was removed *in vacuo.* The crude product was resuspended in methanol (20 mL), filtered and washed with methanol (3 x 8 mL) and hexane (3 x 8 mL) to afford **7** as a bright yellow solid (225 mg, 0.523 mmol, 46%). Rf (TLC) 0.34 (100% EtOAc); IR (ATR, neat) vmax/cm^{-1} 3248 (br, OH), 3038 (w, CH(ar)), 2980w, 2963m, 2916m, 2885m, 2838m, 1552s, 1515s, 1416s, 1392s, 1361m, 1062s, 1015m, 824s, 712s, 567s, 509s; ^{1}H NMR (400 MHz, DMSO-d_6) δH 7.55 (d, 4H, J = 8.4), 7.48 (d, 4H, J = 8.4), 5.35 (s, 2H), 4.58 (s, 4H), 2.58 (s, 6H), 2.45 (s, 6H) ppm; ^{13}C NMR (101 MHz, DMSO-d_6) δC 142.3, 141.2, 137.7, 137.4, 136.1, 127.1, 124.2, 62.4, 14.2, 10.9 ppm; HRMS (ESI-TOF) m/z [M+H]+ Calcd for $C_{24}H_{26}N_6O_2$ 431.2190; Found 431.2182.

(*E*)-1,2-bis(1-(4-(bromomethyl)phenyl)-3,5-dimethyl-1*H*-pyrazol-4-yl)diazene (8)

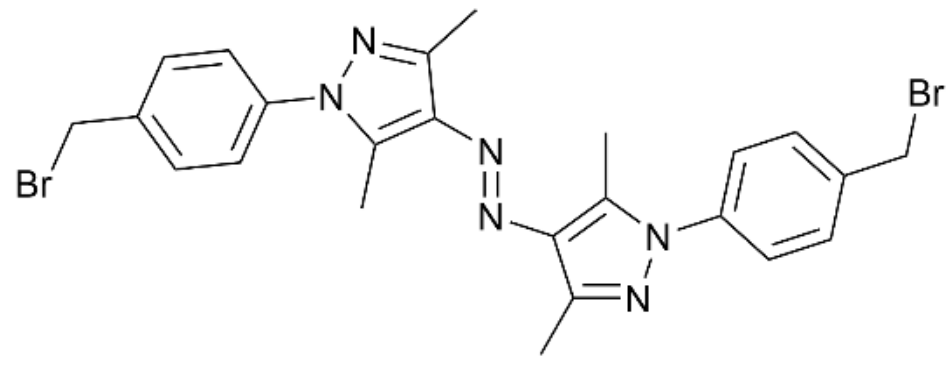

7 (180 mg, 0.418 mmol, 1.0 eq.) was dissolved in aqueous HBr solution (48%, 15 mL) and then the reaction mixture was heated to 50 °C for 27 hours. Following reaction completion, the mixture was cooled to room temperature and added dropwise to 60 mL of water at 0 °C. The precipitate was filtered under vacuum and washed with water, then dried *in vacuo* to afford **8** as an orange solid (181 mg, 0.325 mmol, 78%). Rf (TLC) 0.55 (EtOAc/hexane 1:1); IR (ATR, neat) vmax /cm^{-1} 3289br, 2981w, 2960w, 2920w, 1560m, 1515s, 1413vs, 1390m, 1226m, 1044w, 826w, 725m, 626m, 598vs, 505m; ^{1}H NMR (400 MHz, DMSO-d_6) δH 7.66-7.58 (m, 8H), 4.81 (s, 4H), 2.61 (s, 6H), 2.45 (s, 6H) ppm; ^{13}C NMR (101 MHz, DMSO-d_6) δC 141.5, 138.6, 138.0, 137.6, 136.3, 132.8, 130.3, 127.1, 124.4, 124.2, 62.4, 33.7, 14.2, 10.9 ppm (Degradation observed during measurement of the ^{13}C NMR – suspected hydrolysis to the alcohol product due to the presence of water in DMSO-d6); HRMS (ESI-TOF) m/z [M+H]+ Calcd for $C_{24}H_{24}Br_2N_6$ 555.0502; Found 555.0490.

Molecular Dynamics

Preparation of structures

The linear form of the mutated Bak peptide containing a poly-Arg tag (^{72}GAVGRCLAIFGDCINR^{87}-Ahx-Ahx-RRRR) was generated using PyMOL.[8] Its backbone torsions were seeded with values for a parallel beta sheet. The helical form of

the Bak peptide was taken from the Protein Data Bank (PDB) structure 1BXL[9] (Bcl-xL in complex with Bak peptide) and the mutations Q73A, Q77C, I81F, and D84C and the C-terminal poly-Arg tag were introduced using PyMOL[8] to generate peptide **13**. The AzBisPy linker was subsequently modelled onto these two structures at residues 77 and 84 to generate two stapled peptides: linear Bak stapled with the *E* isomer of the AzBisPy linker and helical Bak stapled with the *Z* isomer of the AzBisPy linker. The N- and C-termini of these peptides were capped with acetyl and amide groups, respectively. Each peptide was then solvated with OPC[10] water molecules in a periodic truncated octahedron box, such that the solvent box walls were at least 10 Å away from the peptide.

Molecular dynamics

Four independent molecular dynamics (MD) simulations using different initial atomic velocities were carried out on the two isomers of AzBisPy-stapled peptide **14** and the initially helical peptide **13**. Energy minimisations and MD simulations were performed by the PMEMD module of AMBER 22[11] using the ff19SB[12] and the generalised AMBER force fields (GAFF).[13] Atomic charges for the stapled residues were derived using the R.E.D. Server,[14] by fitting restrained electrostatic potential (RESP) charges[15] to a molecular electrostatic potential (MEP) computed by the Gaussian 16[16] program at the HF/6-31G* level of theory. To maintain the planar geometry of the pyrazole rings about the azo N-N bond in the *E* isomer of the AzBisPy linker, the X-N-C-X (N denotes the azo nitrogen while C denotes the adjacent pyrazole carbon) dihedral potentials were modified to V_{dih} = 2.576 kcal/mol × [1+ cos(2φ – 180°)].[17] These dihedral potentials were set to be zero, which is the same as that for X-ca-ne-X and X-ca-nf-X in GAFF, for the *Z* isomer. For both isomers, the azo X-N-N-X dihedral potential was set to V_{dih} = 16.94 kcal/mol × [1+ cos(2φ – 180°)] + 3.79 kcal/mol × [1+ cos(φ)] + 0.34 kcal/mol × [1+ cos(3φ)].[11]

The SHAKE[18] algorithm was applied to constrain all bonds to hydrogen atoms, thus allowing for a time step of 2 fs. A cutoff distance of 9 Å was implemented for nonbonded interactions. The particle mesh Ewald method[19] was used to treat long-range electrostatic interactions under periodic boundary conditions. Each system underwent 1000 cycles of steepest-descent energy minimisation followed by 1000 cycles of conjugate-gradient energy minimisation. The system was then gradually heated to 300 K over 50 ps at constant volume before equilibration at a constant pressure of 1 atm for another 50 ps. Harmonic positional restraints with a force constant of 2.0 kcal mol^{-1} $Å^{-2}$ were placed on the non-hydrogen atoms of the peptide during these energy minimisation and equilibration steps. Subsequent unrestrained equilibration (2 ns) and production (500 ns) runs were carried out at 300 K and 1 atm, using a Langevin thermostat[20] with a collision frequency of 2 ps^{-1} to maintain the temperature and a Berendsen barostat[21] with a pressure relaxation time of 2 ps to maintain the pressure.

Conformational clustering

The last 300 ns of the four individual runs for each system were combined into a single trajectory for clustering analysis. Backbone atoms of the peptide residues were clustered using the root-mean-square-deviation-based kclust clustering method in the MMTSB toolset.[22] Cluster radii were empirically chosen to produce well-separated clusters for each of the systems.

NMR Spectra

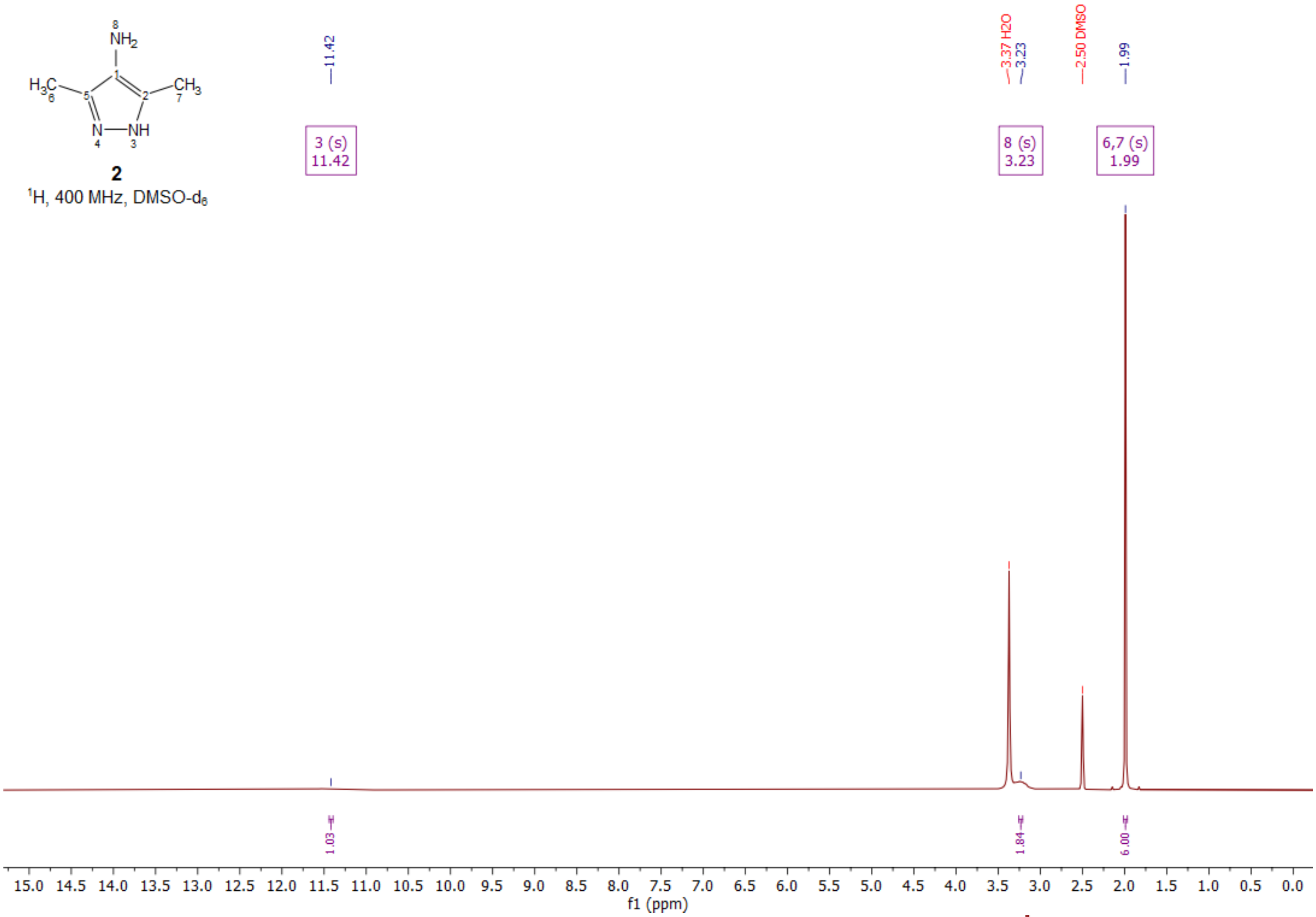

2

^{1}H, 400 MHz, DMSO-d_6

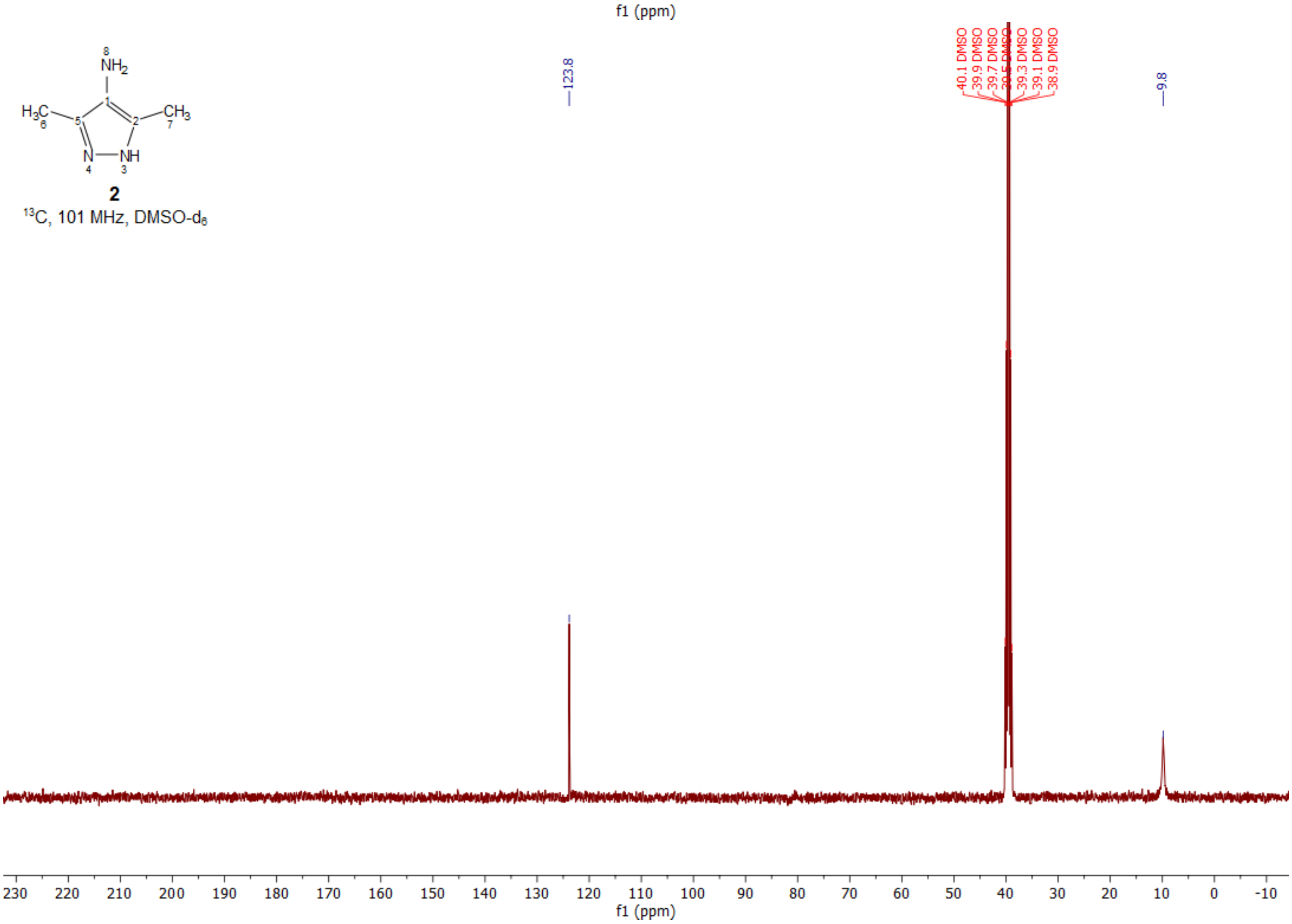

2

^{13}C, 101 MHz, DMSO-d_6

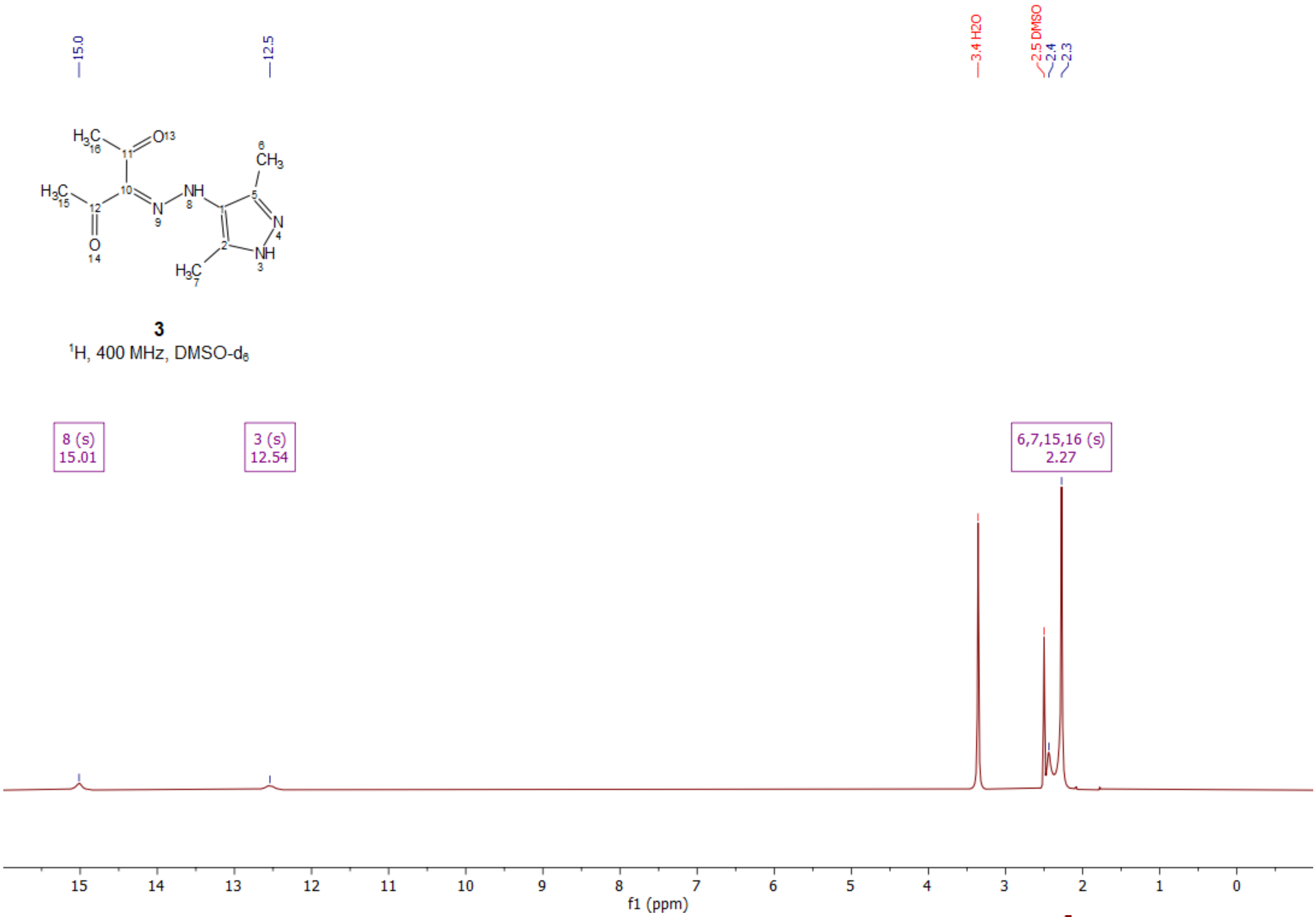

3

^{1}H, 400 MHz, DMSO-d_6

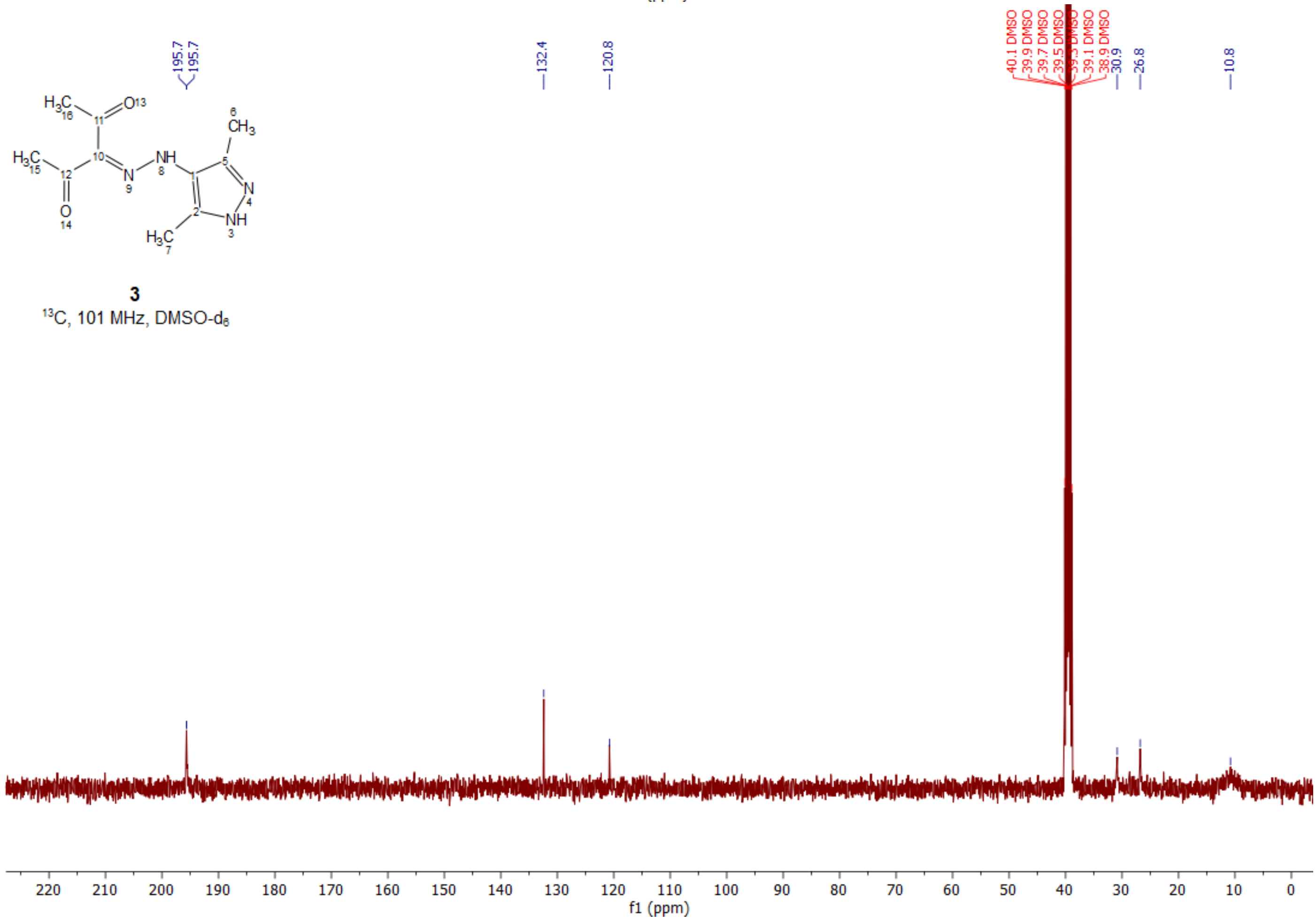

3

^{13}C, 101 MHz, DMSO-d_6

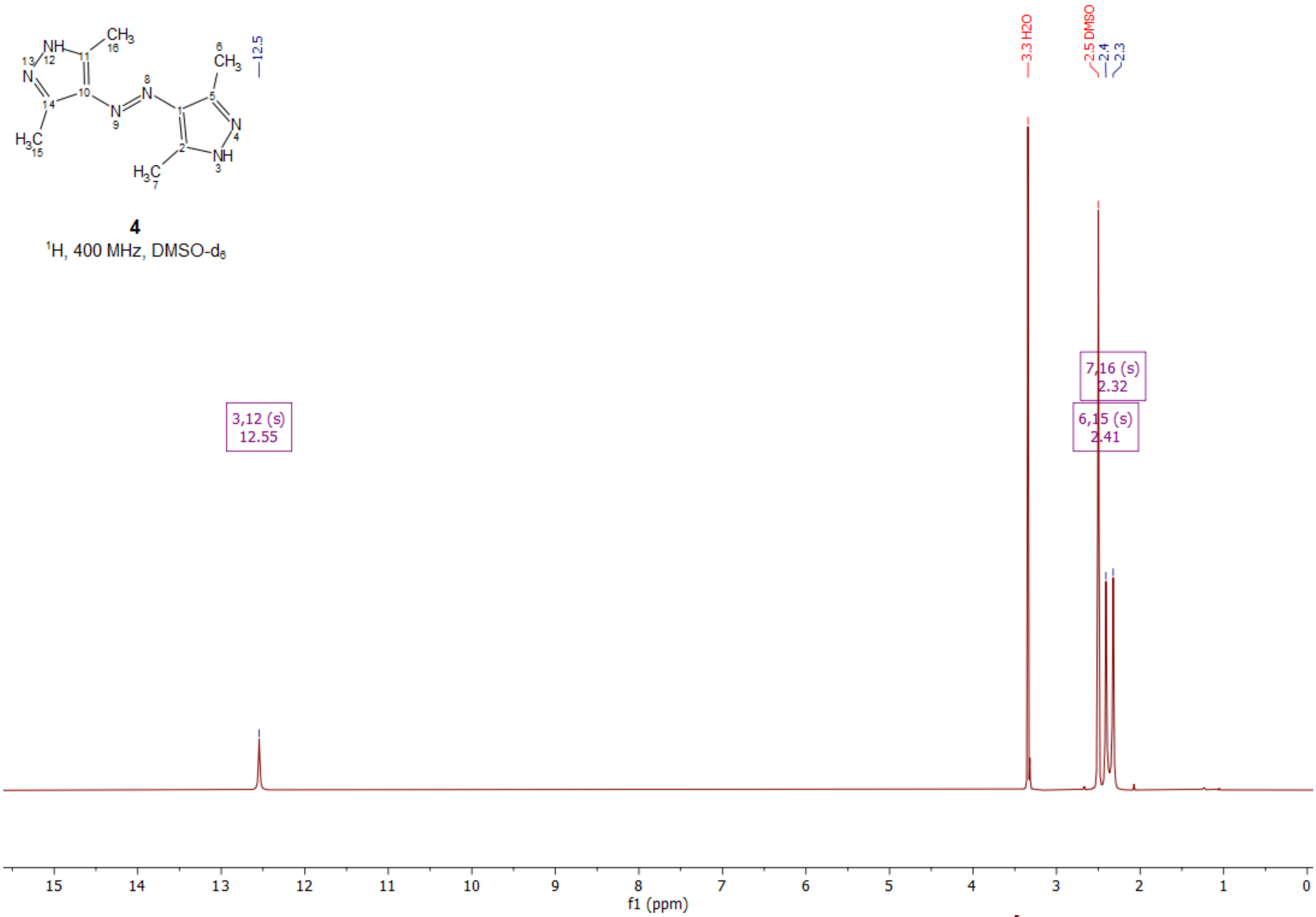
4
¹H, 400 MHz, DMSO-d6
12.5
3.3 H2O
2.5 DMSO
2.4
2.3
3,12 (s)
12.55
7,16 (s)
2.32
6,15 (s)
2.41
f1 (ppm)

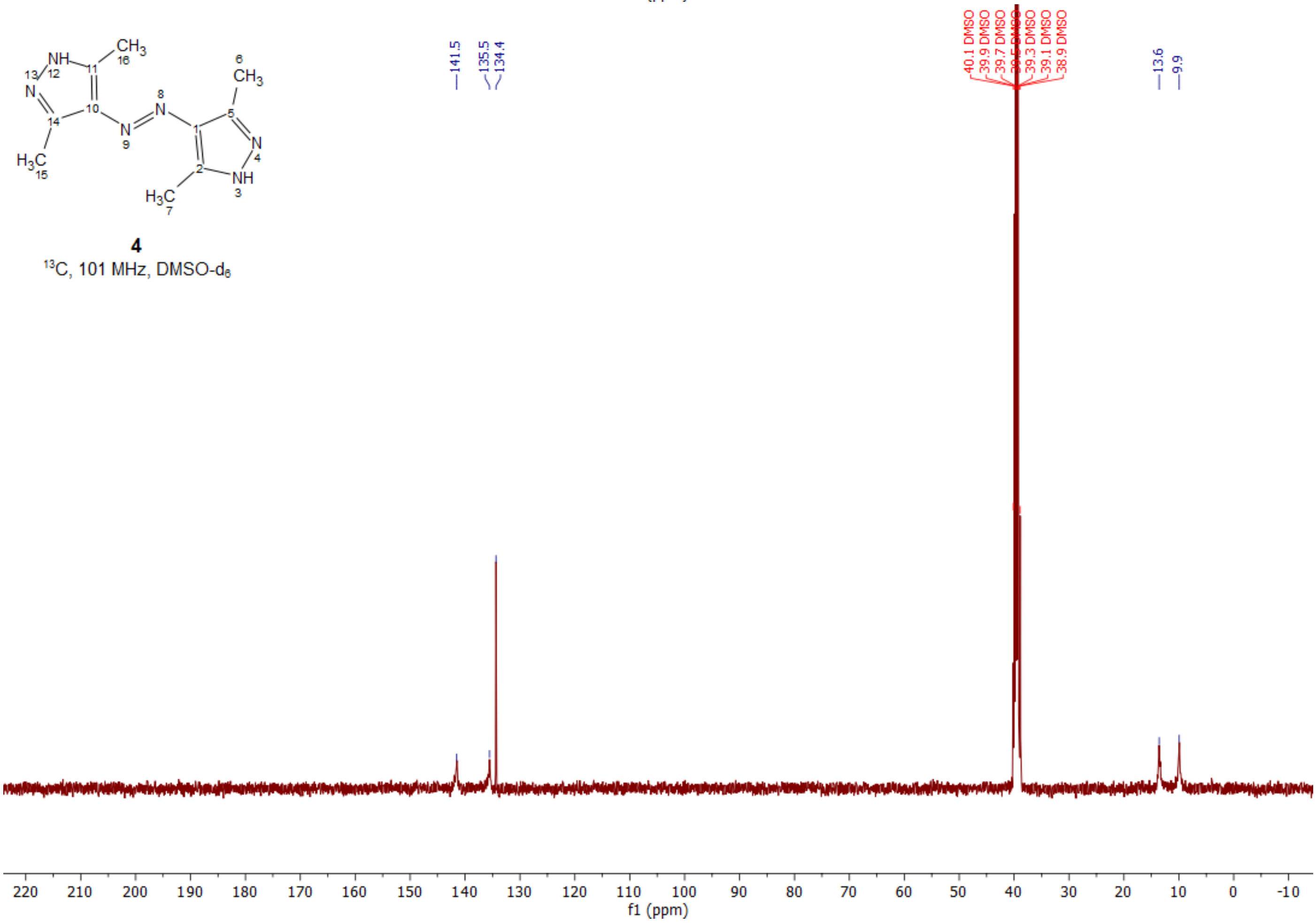
4
¹³C, 101 MHz, DMSO-d6
141.5
135.5
134.4
40.1 DMSO
39.9 DMSO
39.7 DMSO
39.3 DMSO
39.1 DMSO
38.9 DMSO
13.6
9.9
f1 (ppm)

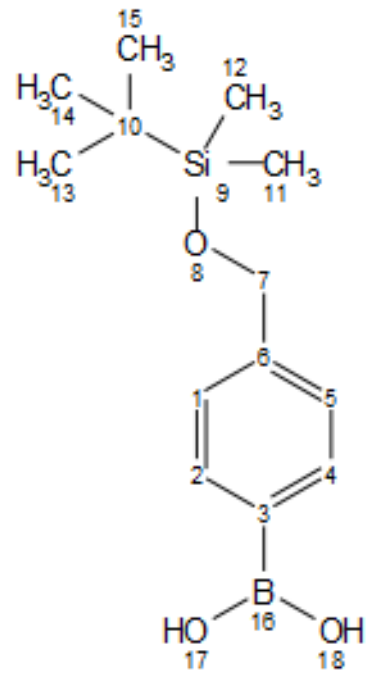

5

^{1}H, 400 MHz, $CDCl_3$

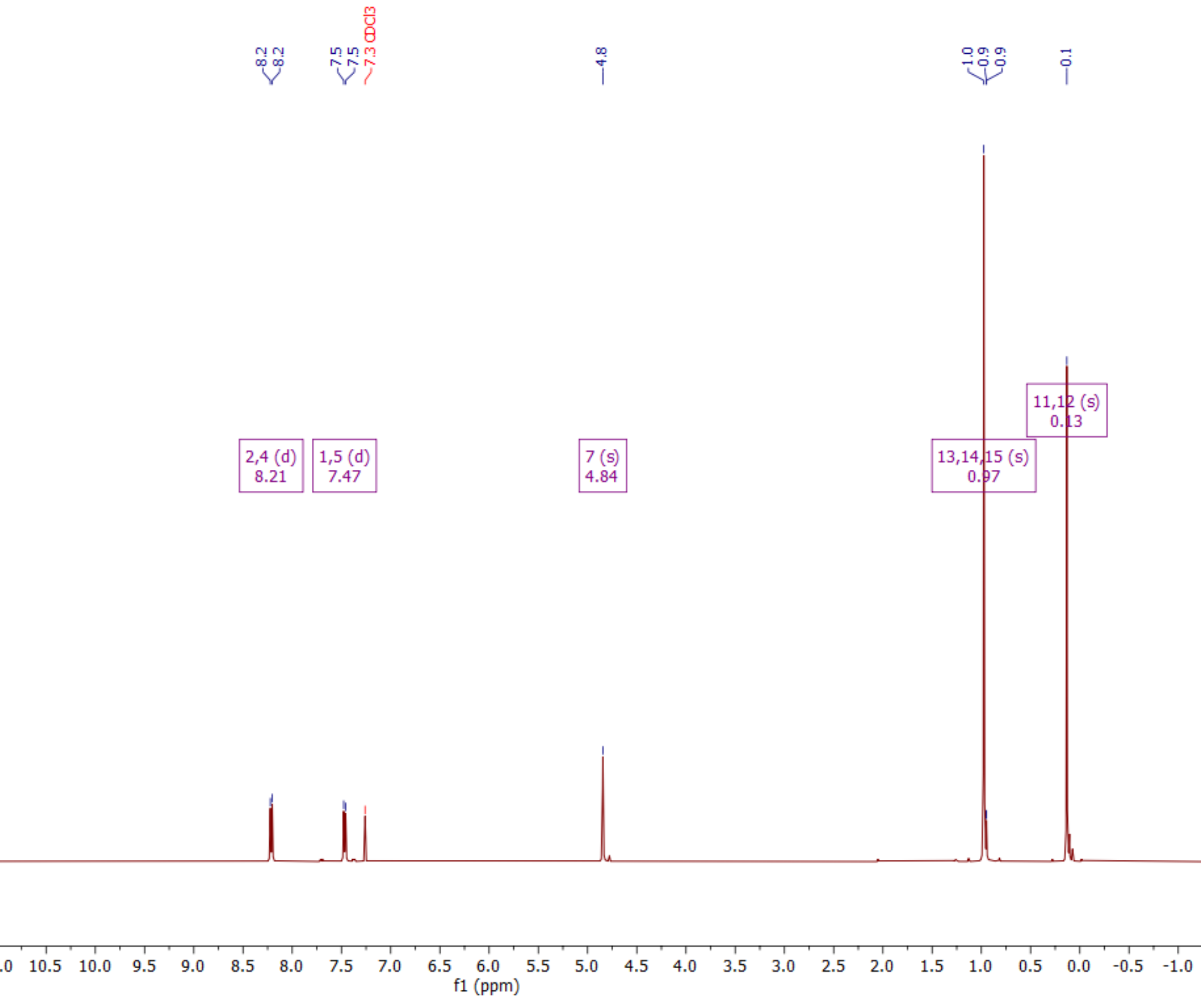

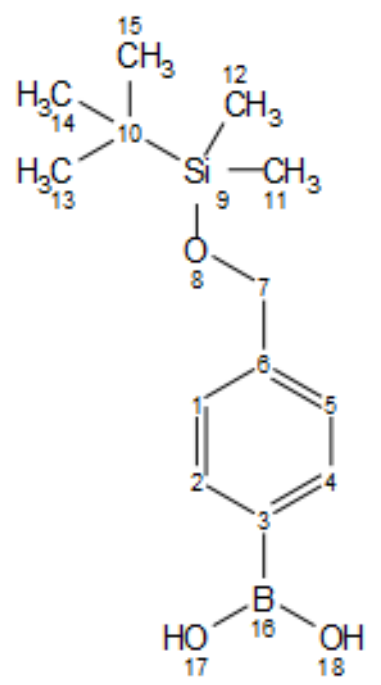

5

^{13}C, 101 MHz, $CDCl_3$

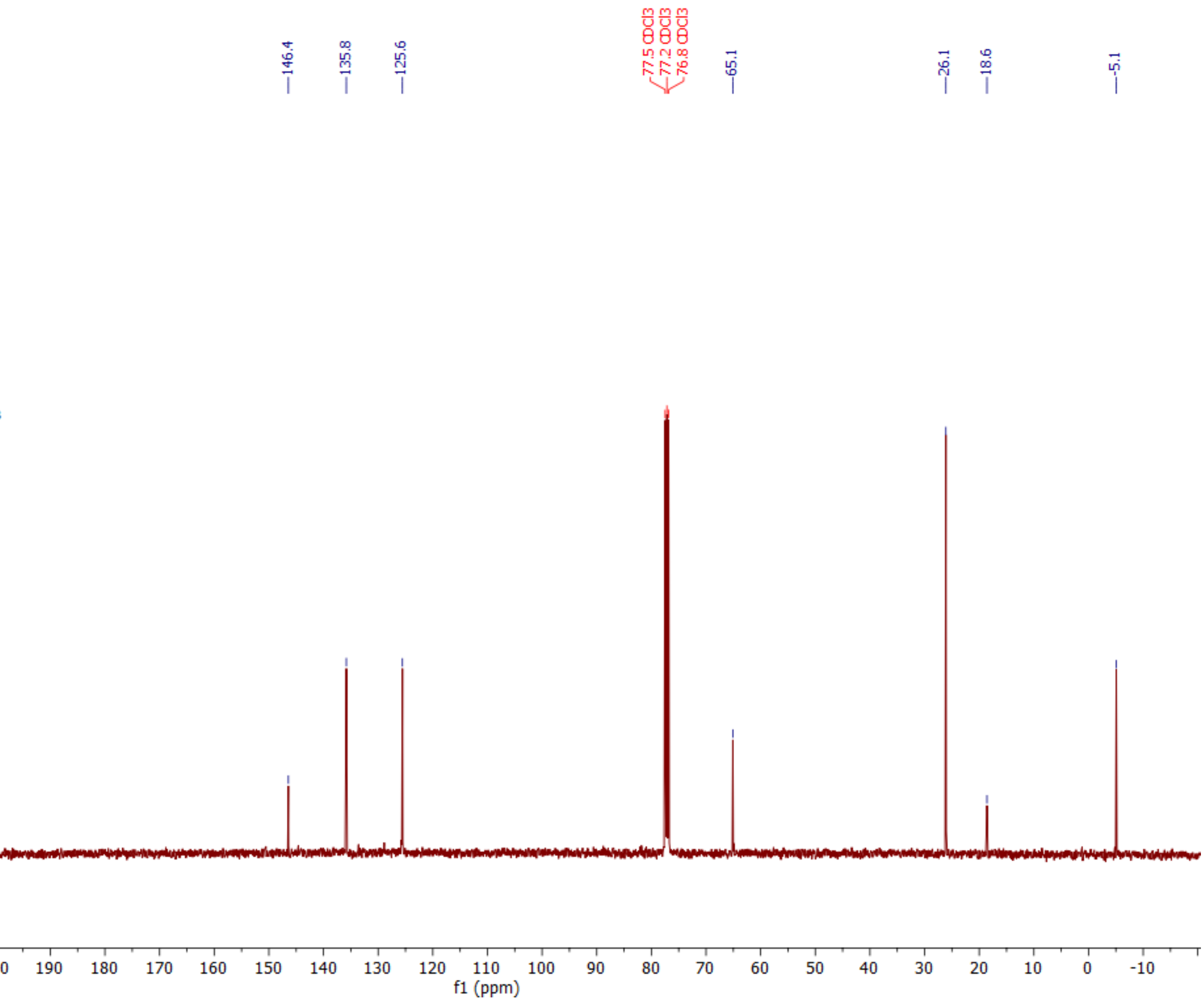

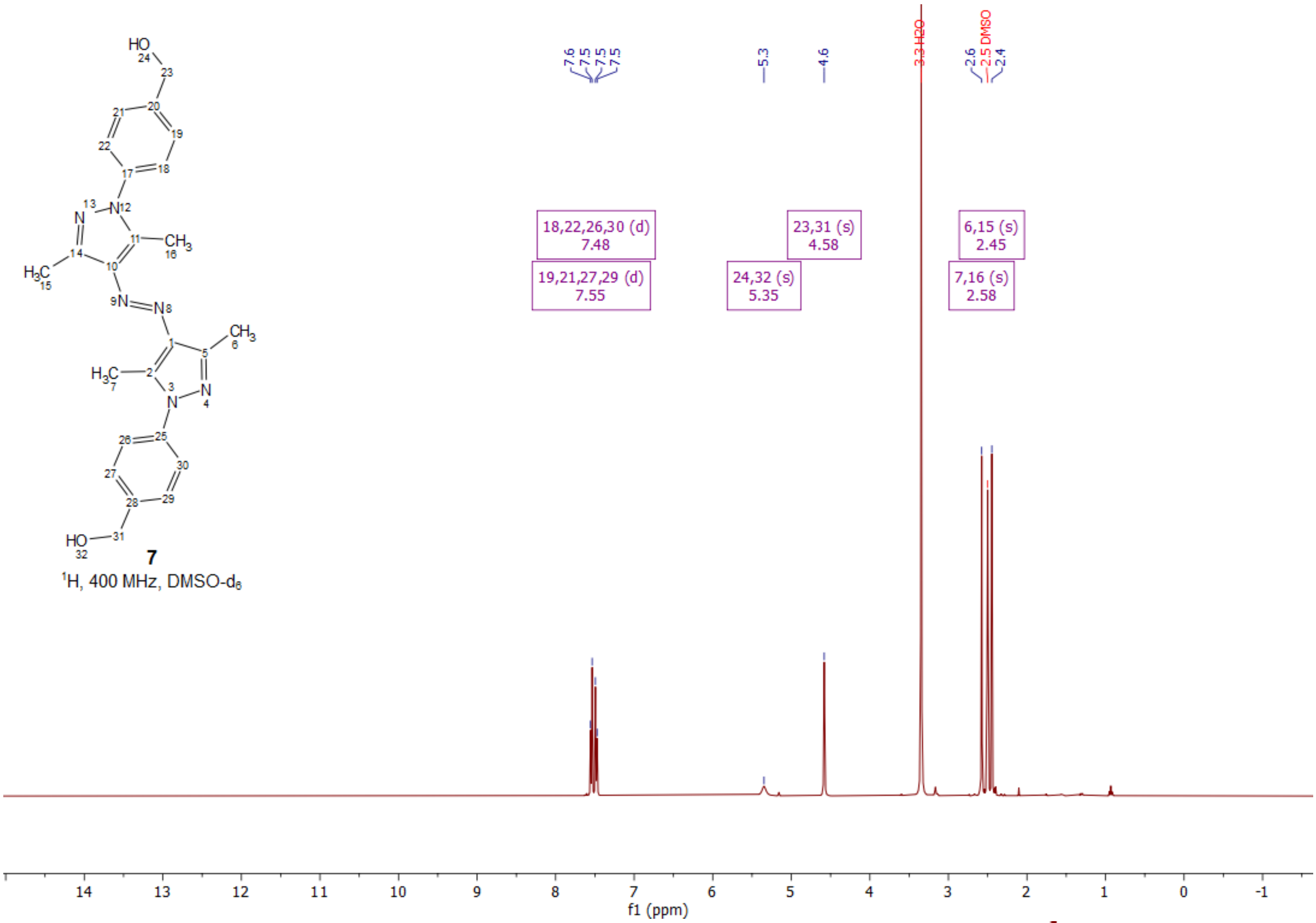

7
^{1}H, 400 MHz, DMSO-d$_6$
7.6 7.5 7.5 7.5
5.3
4.6
3.3 H2O
2.6 2.5 DMSO 2.4
18,22,26,30 (d) 7.48
19,21,27,29 (d) 7.55
23,31 (s) 4.58
24,32 (s) 5.35
6,15 (s) 2.45
7,16 (s) 2.58
f1 (ppm)

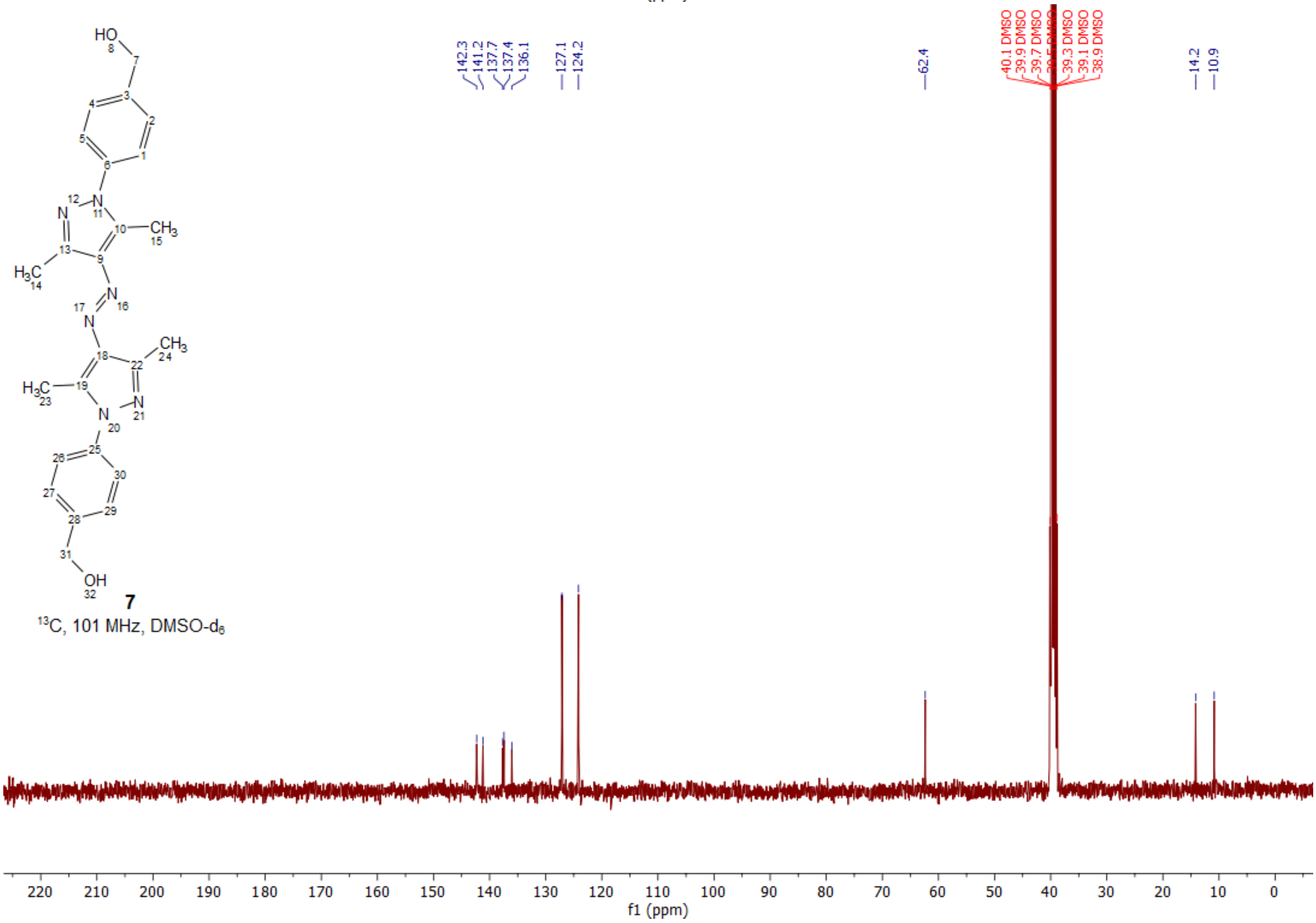

7
^{13}C, 101 MHz, DMSO-d$_6$
142.3 141.2 137.7 137.4 136.1
127.1 124.2
62.4
40.1 DMSO 39.9 DMSO 39.7 DMSO 39.5 DMSO 39.3 DMSO 39.1 DMSO 38.9 DMSO
14.2 10.9
f1 (ppm)

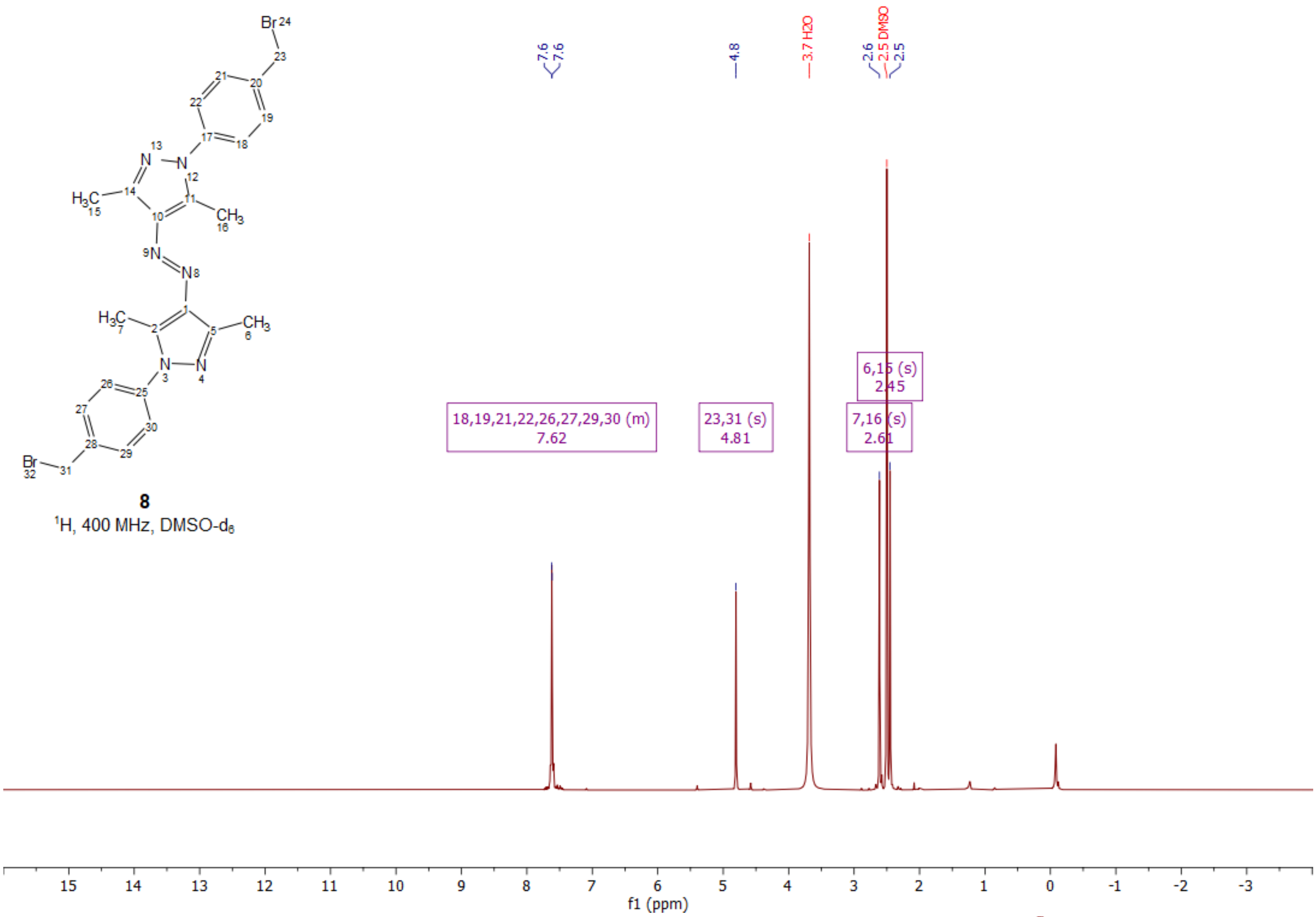
8
^{1}H, 400 MHz, DMSO-d$_6$
18,19,21,22,26,27,29,30 (m) 7.62
23,31 (s) 4.81
7,16 (s) 2.61
6,15 (s) 2.45
3.7 H2O
2.5 DMSO
f1 (ppm)

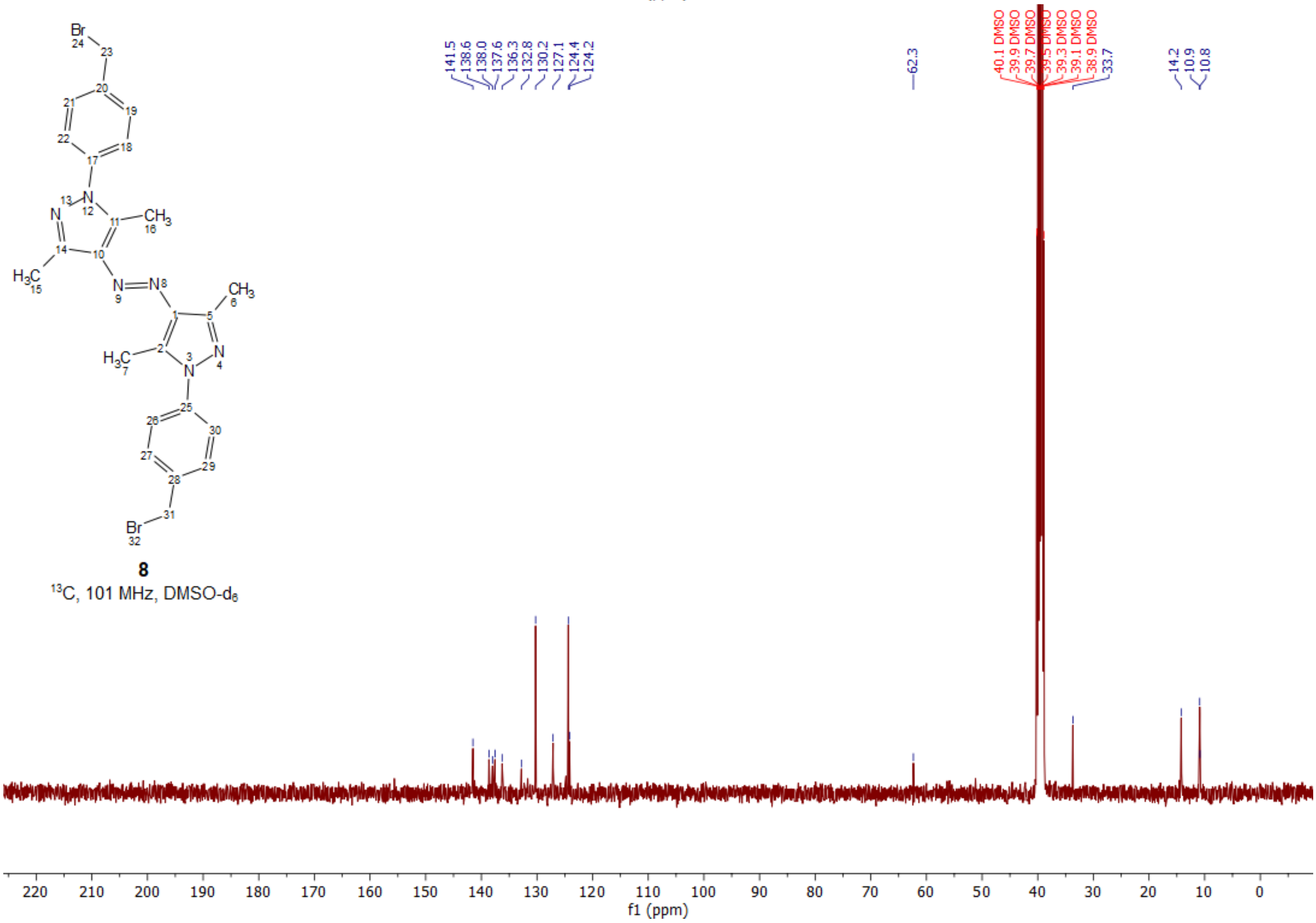
8
^{13}C, 101 MHz, DMSO-d$_6$
141.5
138.6
138.0
137.6
136.3
132.8
130.2
127.1
124.4
124.2
62.3
40.1 DMSO
39.9 DMSO
39.7 DMSO
39.5 DMSO
39.3 DMSO
39.1 DMSO
38.9 DMSO
33.7
14.2
10.9
10.8
f1 (ppm)

References

1. A. Müller, H. Kobarg, V. Chandrasekaran, J. Gronow, F. D. Sönnichsen and T. K. Lindhorst, *Chem. – Eur. J.*, 2015, **21**, 13723-13731.
2. A. I. Green, F. Hobor, C. P. Tinworth, S. Warriner, A. J. Wilson and A. Nelson, *Chemistry*, 2020, **26**, 10682-10689.
3. J. P. Plante, T. Burnley, B. Malkova, M. E. Webb, S. L. Warriner, T. A. Edwards and A. J. Wilson, *Chem. Commun.*, 2009, https://doi.org/10.1039/B908207G5091-5093.
4. X. Zhu, H. Miao, Y. Shan, G. Gao, Q. Gu, Q. Xiao and X. He, *Inorg. Chem.*, 2022, **61**, 13591-13599.
5. S. Millan, J. Nasir, B. Gil-Hernández, T.-O. Knedel, B. Moll, I. Boldog, O. Weingart, J. Schmedt auf der Günne and C. Janiak, *Cryst. Growth Des.*, 2020, **20**, 2721-2733.
6. S. Millan, B. Gil-Hernández, E. Hastürk, A. Schmitz and C. Janiak, *Z. Anorg. Allg. Chem.*, 2018, **644**, 1311-1316.
7. P. R. Ashton, R. Ballardini, V. Balzani, A. Credi, K. R. Dress, E. Ishow, C. J. Kleverlaan, O. Kocian, J. A. Preece, N. Spencer, J. F. Stoddart, M. Venturi and S. Wenger, *Chem. – Eur. J.*, 2000, **6**, 3558-3574.
8. The PyMOL Molecular Graphics System, Schrödinger, LLC.
9. M. Sattler, H. Liang, D. Nettesheim, R. P. Meadows, J. E. Harlan, M. Eberstadt, H. S. Yoon, S. B. Shuker, B. S. Chang, A. J. Minn, C. B. Thompson and S. W. Fesik, *Science*, 1997, **275**, 983-986.
10. S. Izadi, R. Anandakrishnan and A. V. Onufriev, *The journal of physical chemistry letters*, 2014, **5**, 3863-3871.
11. D. A. Case, H. M. Aktulga, K. Belfon, I. Y. Ben-Shalom, J. T. Berryman, S. R. Brozell, D. S. Cerutti, T. E. Cheatham, III, G. A. Cisneros, V. W. D. Cruzeiro, T. A. Darden, R. E. Duke, G. Giambasu, M. K. Gilson, H. Gohlke, A. W. Goetz, R. Harris, S. Izadi, S. A. Izmailov, K. Kasavajhala, M. C. Kaymak, E. King, A. Kovalenko, T. Kurtzman, T. S. Lee, S. LeGrand, P. Li, C. Lin, J. Liu, T. Luchko, R. Luo, M. Machado, V. Man, M. Manathunga, K. M. Merz, Y. Miao, O. Mikhailovskii, G. Monard, H. Nguygen, K. A. O'Hearn, A. Onufriev, F. Pan, S. Pantano, R. Qi, A. Rahnamoun, D. R. Roe, A. Roitberg, C. Sagui, S. Schott-Verdugo, A. Shajan, J. Shen, C. L. Simmerling, N. R. Skrynnikov, J. Smith, J. Swails, R. C. Walker, J. Wang, J. Wang, H. Wei, R. M. Wolf, X. Wu, Y. Xiong, Y. Xue, D. M. York, S. Zhao and P. A. Kollman, 2022, AMBER 22, University of California, San Francisco.
12. C. Tian, K. Kasavajhala, K. A. Belfon, L. Raguette, H. Huang, A. N. Migues, J. Bickel, Y. Wang, J. Pincay and Q. Wu, *J. Chem. Theory Comput.*, 2019, **16**, 528-552.
13. J. M. Wang, R. M. Wolf, J. W. Caldwell, P. A. Kollman and D. A. Case, *J. Comput. Chem.*, 2004, **25**, 1157-1174.
14. E. Vanquelef, S. Simon, G. Marquant, E. Garcia, G. Klimerak, J. C. Delepine, P. Cieplak and F.-Y. Dupradeau, *Nucleic Acids Res.*, 2011, **39**, W511-W517.
15. W. D. Cornell, P. Cieplak, C. I. Bayly and P. A. Kollman, *J. Am. Chem. Soc.*, 1993, **115**, 9620-9631.
16. M. J. Frisch, G. W. Trucks, H. B. Schlegel, G. E. Scuseria, M. A. Robb, J. R. Cheeseman, G. Scalmani, V. Barone, G. A. Petersson, H. Nakatsuji, X. Li, M. Caricato, A. V. Marenich, J. Bloino, B. G. Janesko, R. Gomperts, B. Mennucci, H. P. Hratchian, J. V. Ortiz, A. F. Izmaylov, J. L. Sonnenberg, Williams, F. Ding, F. Lipparini, F. Egidi, J. Goings, B. Peng, A. Petrone, T. Henderson, D. Ranasinghe, V. G. Zakrzewski, J. Gao, N. Rega, G. Zheng, W. Liang, M. Hada, M. Ehara, K. Toyota, R. Fukuda, J. Hasegawa, M. Ishida, T. Nakajima, Y. Honda, O. Kitao, H. Nakai, T. Vreven, K. Throssell, J. A. Montgomery Jr., J. E. Peralta, F. Ogliaro, M. J. Bearpark, J. J. Heyd, E. N. Brothers, K. N. Kudin, V. N. Staroverov, T. A. Keith, R. Kobayashi, J. Normand, K. Raghavachari, A. P. Rendell, J. C. Burant, S. S. Iyengar, J. Tomasi, M. Cossi, J. M. Millam, M. Klene, C. Adamo, R. Cammi, J. W. Ochterski, R. L. Martin, K. Morokuma, O. Farkas, J. B. Foresman and D. J. Fox, 2016, Gaussian 16 Rev. C.01, Wallingford, CT.
17. P. Duchstein, C. Neiss, A. Görling and D. Zahn, *J. Mol. Model.*, 2012, **18**, 2479-2482.
18. J. P. Ryckaert, G. Ciccotti and H. J. C. Berendsen, *Journal of Computational Physics*, 1977, **23**, 327-341.
19. T. Darden, D. York and L. Pedersen, *J. Chem. Phys.*, 1993, **98**, 10089-10092.
20. J. A. Izaguirre, D. P. Catarello, J. M. Wozniak and R. D. Skeel, *J. Chem. Phys.*, 2001, **114**, 2090-2098.
21. H. J. C. Berendsen, J. P. M. Postma, W. F. Vangunsteren, A. Dinola and J. R. Haak, *J. Chem. Phys.*, 1984, **81**, 3684-3690.
22. M. Feig, J. Karanicolas and C. L. Brooks, *J. Mol. Graphics Modell.*, 2004, **22**, 377-395.
